# Cassava genome from a wild ancestor to cultivated varieties

**DOI:** 10.1038/ncomms6110

**Published:** 2014-10-10

**Authors:** Wenquan Wang, Binxiao Feng, Jingfa Xiao, Zhiqiang Xia, Xincheng Zhou, Pinghua Li, Weixiong Zhang, Ying Wang, Birger Lindberg Møller, Peng Zhang, Ming-Cheng Luo, Gong Xiao, Jingxing Liu, Jun Yang, Songbi Chen, Pablo D. Rabinowicz, Xin Chen, Hong-Bin Zhang, Henan Ceballos, Qunfeng Lou, Meiling Zou, Luiz J.C.B. Carvalho, Changying Zeng, Jing Xia, Shixiang Sun, Yuhua Fu, Haiyan Wang, Cheng Lu, Mengbin Ruan, Shuigeng Zhou, Zhicheng Wu, Hui Liu, Rubini Maya Kannangara, Kirsten Jørgensen, Rebecca Louise Neale, Maya Bonde, Nanna Heinz, Wenli Zhu, Shujuan Wang, Yang Zhang, Kun Pan, Mingfu Wen, Ping-An Ma, Zhengxu Li, Meizhen Hu, Wenbin Liao, Wenbin Hu, Shengkui Zhang, Jinli Pei, Anping Guo, Jianchun Guo, Jiaming Zhang, Zhengwen Zhang, Jianqiu Ye, Wenjun Ou, Yaqin Ma, Xinyue Liu, Luke J. Tallon, Kevin Galens, Sandra Ott, Jie Huang, Jingjing Xue, Feifei An, Qingqun Yao, Xiaojing Lu, Martin Fregene, L. Augusto Becerra López-Lavalle, Jiajie Wu, Frank M. You, Meili Chen, Songnian Hu, Guojiang Wu, Silin Zhong, Peng Ling, Yeyuan Chen, Qinghuang Wang, Guodao Liu, Bin Liu, Kaimian Li, Ming Peng

**Affiliations:** 1 Institute of Tropical Biosciences and Biotechnology, Chinese Academy of Tropical Agricultural Sciences (CATAS), Haikou 571101, China; 2 Tropical Crop Genetic Resources Institute, CATAS, Danzhou 571700, China; 3 Beijing Institute of Genomics, Chinese Academy of Sciences (CAS), Beijing 100101, China; 4 Department of Computer Science and Engineering and Department of Genetics, Washington University, Saint Louis, Missouri 63130, USA; 5 Institute for Systems Biology, Jianghan University, Wuhan 430056, China; 6 South China Botanical Garden, CAS, Guangzhou 510650, China; 7 Plant Biochemistry Laboratory, Department of Plant and Environmental Sciences, University of Copenhagen, Copenhagen 1165, Denmark; 8 Institute of Plant Physiology and Ecology, Shanghai Institutes for Biological Sciences of CAS, Shanghai 200032, China; 9 Department of Plant Sciences, University of California, Davis, California 95616, USA; 10 Institute for Genome Sciences, University of Maryland School of Medicine, Baltimore, Maryland 21201, USA; 11 Department of Soil and Crop Sciences, Texas A&M University, College Station, Texas 77843, USA; 12 International Center for Tropical Agriculture (CIAT), Cali 6713, Colombia; 13 State Key Laboratory of Crop Genetics and Germplasm Enhancement, College of Horticulture, Nanjing Agricultural University, Nanjing 210095, China; 14 Brazilian Enterprise for Agricultural Research (EMBRAPA), Genetic Resources and Biotechnology, Brasilia 70770, Brazil; 15 Shanghai Key Lab of Intelligent Information Processing, and School of Computer Science, Fudan University, Shanghai 200433, China; 16 State Key Laboratory of Agrobiotechnology, School of Life Sciences, Chinese University of Hong Kong, Hong Kong, China; 17 Citrus Research and Education Center (CREC), University of Florida, Gainesville, Florida 32611, USA; 18 State Key Laboratory of Desert and Oasis Ecology, Key Laboratory of Biogeography and Bioresources in Arid Land, Center of Systematic Genomics, Xinjiang Institute of Ecology and Geography, Urumqi 830011, China

## Abstract

Cassava is a major tropical food crop in the Euphorbiaceae family that has high carbohydrate production potential and adaptability to diverse environments. Here we present the draft genome sequences of a wild ancestor and a domesticated variety of cassava and comparative analyses with a partial inbred line. We identify 1,584 and 1,678 gene models specific to the wild and domesticated varieties, respectively, and discover high heterozygosity and millions of single-nucleotide variations. Our analyses reveal that genes involved in photosynthesis, starch accumulation and abiotic stresses have been positively selected, whereas those involved in cell wall biosynthesis and secondary metabolism, including cyanogenic glucoside formation, have been negatively selected in the cultivated varieties, reflecting the result of natural selection and domestication. Differences in microRNA genes and retrotransposon regulation could partly explain an increased carbon flux towards starch accumulation and reduced cyanogenic glucoside accumulation in domesticated cassava. These results may contribute to genetic improvement of cassava through better understanding of its biology.

## Abstract (web summary)

Cassava is a major source of food in tropical and subtropical regions. Here the authors sequence the genomes of wild and domesticated cassava varieties and identify genes that have been selected for and against during the evolution and domestication of this economically important crop.

## Graphical abstract

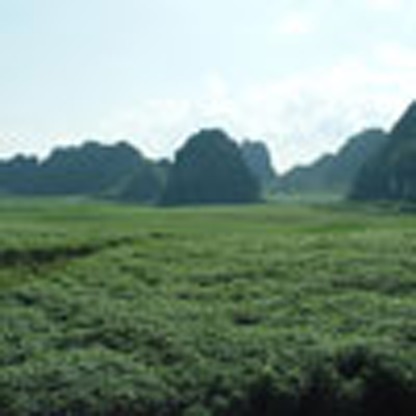

Cultivated cassava, *Manihot esculenta* Crantz, originated from its wild progenitor, *Manihot esculenta* ssp*. Flabellifolia*, in tropical lowlands along the southern rim of the Amazon basin, where sunlight, heat and rainfall are plentiful, and intervals of drought are common^1^,^2^,^3^. Domestication of cassava occurred during the period of approximately 12,000 to 7,000 years ago by indigenous South Americans, as supported by DNA sequence analysis of a single locus^3^, and by archaeological and fossil records^4^,^5^, resulting in the modern cassava cultivars with extraordinary characteristics including high biomass and high starch yield in near optimum environments, and tolerance to drought and barren soil. Cassava is the most important root crop worldwide and provides staple food for over 700 million people in Africa (51%), Asia (29%) and South America (20%; http://faostat.fao.org). As it is highly tolerant to drought and its storage roots can be preserved in soil for a few years, cassava is considered to be an important reserve of carbohydrates to relieve global famine^6^. It is also an ideal feedstock crop for bioenergy, biomaterials and animal feeds because of its favourable agricultural characteristics and high-quantity and -quality starch^7^,^8^.

The cassava genome (2*n*=36)^9^ is highly heterozygous because of its outcrossing nature and broad tropical distribution^10^,^11^. Conventional breeding and marker-assisted selection^12^,^13^,^14^ have so far proved ineffective in achieving its potential regarding desirable traits, such as high-quality starch, storage root yield, avoidance to postharvest biological deterioration and resistance to diseases. For instance, cassava storage root yield is approximately 13.6 t ha^−1^ globally, which is two- to fourfold below its potential productivity. The lack of a reference genome sequence and other genomic and transcriptomic resources has limited progress in basic biological research and breeding in cassava. Therefore, the draft genome sequence of a partial inbred cassava line, AM560, has been generated and publicly released relatively recently^15^ (http://www.phytozome.net/cassava.php/). The sequence integrated 26- and 0.9-fold coverage of Roche 454 and Sanger reads, resulting in 530-Mb assembled scaffolds (including 410-Mb of contigs with no gaps), that cover approximately 70% of the cassava genome.

In the present study, we sequence the genomes of two cassava genotypes: W14 (*Manihot esculenta* ssp*. flabellifolia*), a wild subspecies that shows low storage root yield and low root starch content; and KU50, a variety commonly cultivated in Southeast Asia that has six to eight times higher storage root yield potential and five to six times higher starch content than W14 as described in Supplementary Information. Using an integrated assembly strategy combining shotgun Illumina and Roche 454 reads, and a bacterial artificial chromosome (BAC)-based physical map with BAC-end Sanger sequences, we generate a high-quality draft genome sequence of cassava using established protocols^16^,^17^,^18^,^19^,^20^. In addition, the genome of a self-pollinated clone (S1.600) derived from the *sugary* cassava landrace CAS36 (ref. 21) is sequenced to 20-fold coverage, and the transcriptomes of W14, KU50 and another cultivated variety Arg7 are profiled during plant ontogeny. A comparative analysis of the three genome sequences and annotated transcriptomes enables us to better understanding genomic features underlying the evolution and domestication of cassava^22^,^23^,^24^, particularly in relation to carbon flux, starch synthesis and biosynthesis of cyanogenic compounds. These genomic resources and findings provide a platform for advancing basic biology research, gene discovery and genomic selection-assisted breeding in cassava^25^,^26^.

## Results

### Genome assembly and annotation

The genomes of cassava lines, W14 and KU50 (Supplementary Fig. 1, Supplementary Table 1, Supplementary Note 1) were sequenced and *de novo* assembled using next-generation sequencing technologies and hybrid assembly approaches^27^ (Supplementary Note 5). For W14, a 432-Mb assembly with an N50 of 43 kb was obtained. The assembly spanned 58.2% of the 742-Mb cassava genome, estimated by the kmer-spectrum (Supplementary Note 3, Supplementary Figure 2), and 92% of the sequence were non-gapped contigs. For KU50, the assembly spanned 495 Mb representing 66.7% of the cassava genome. The N50 was 19 kb and 81% sequences were non-gapped contigs (Supplementary Note 5–8, Supplementary Figs 3–5, Supplementary Tables 3 and 6). The quality of the draft genome sequence of W14 was evaluated by aligning the Roche 454 sequences of five BAC clones (Supplementary Fig. 6). In this way, we determined that the average error rate between the BAC and the draft genome sequence was less than 0.61% (Supplementary Table 5). The detected errors were single-nucleotide mismatches and insertions/deletions. These results show that the W14 draft genome sequence assembly is of high quality in spite of its high heterozygosity (Supplementary Note 11; Supplementary Table 9) and complexity. The genome coverage and quality of these assemblies were further confirmed by 201,392 available transcript sequences of W14 and KU50. Specifically, 94.9% and 92.8% of the transcripts could be aligned to the genome assemblies of W14 and KU50, respectively (Supplementary Figs 7, 8 and 9a,b). Using transcriptomics data and the *ab initio* gene prediction, 34,483 and 38,845 genes were predicted in the W14 and KU50 genomes, respectively. Comparison to protein databases, predicted 33,310 (96.6%) protein-coding genes in W14 and 37,592 (96.8%) in KU50 (using *E*-value cutoff of 10^−5^) and tentative functions could be assigned (Supplementary Note 9 and 10, Supplementary Figs 10, 12, Supplementary Table 7).

**Figure 1:**
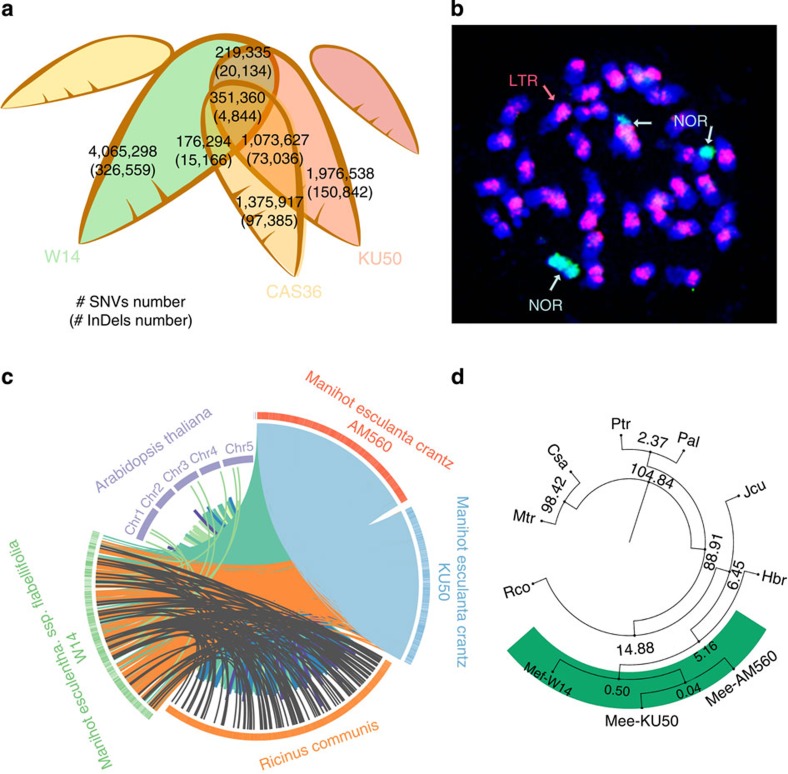
Cassava comparative genomes. (**a**) Venn diagram of SNVs/InDels diversity of the cassava genomes of W14, KU50 and CAS36 sequenced in this study with comparison to the AM560 genome sequences previously released. The number of SNVs is listed and the number of InDels is shown in parentheses. (**b**) Chromosome *in situ* hybridization showing the repeated occurrence of 45S (Nucleolus organizer, NOR), LTR and chromosome numbers (2*n*=36) of cultivar KU50. (**c**) A CirCOS (http://circos.ca/) figure showing synteny between three paralogous cassava genomic regions and their putative orthologues present in *R. communis* and *A. thaliana* genomes. Coloured lines connect the cassava scaffolds to the *A. thaliana* chromosomes and *R. communis* scaffolds. The line distances across different scaffolds denote the similarities of the segments, with a longer line indicating a higher similarity. (**d**) Gene tree showing the divergence time of the wild ancestor subspecies to cultivars, referenced to neighbour species in the Euphorbiaceae family inferred from sequence comparison to 71 chloroplast genes from eight different plant species. Mtr: *Medicago truncatula*, Csa: *Cucumis sativus*, Ptr: *Populus trichocarpa*, Pni: *Populus nigra*, Ptd: *Populus trichocarpa* x *Populus deltoids*, Rco: *Ricinus communis*, Ees: *Euphorbia esula*, Jcu: *Jatropha curcas*, Mef-W14: *Manihot esculenta* ssp. *flabellifolia* (W14), Mes-KU50: *Manihot esculenta* ssp. *esculenta* (KU50), Mes-AM560: *Manihot esculenta* ssp. *esculenta* (cultivar AM560).

The genome sequence assembly was searched for repetitive DNA using *de novo* approaches that identified 36.9% and 25.7% of the W14 and KU50 genomes as repetitive sequences, respectively. The majority of the repetitive elements were long interspersed nuclear elements and long-terminal repeat elements (LTRs, Supplementary Table 8). These results, in addition to the fact that around 35% of the genome could not be assembled, suggest that the cassava genome is highly heterochromatic. This was confirmed by chromosome *in-situ* hybridization using an LTR probe (Fig. 1b).

The level of heterozygosity in cassava is among the highest found in sequenced plant genomes, as determined by the frequency of single-nucleotide variations (SNVs) and insertions and deletions (InDels) in its genome. We identified 3.8 and 3.4 SNVs per kilo-base (kb) in the W14 and the KU50 genomes, respectively (Supplementary Table 9), which are much higher levels than those found in bamboo (1.0 per kb)^28^, peach (1.5 per kb)^29^ and poplar (2.6 per kb)^30^, while they are comparable to those of grape (4.2 per kb)^31^ and potato (4.3 per kb)^18^. Cassava’s high heterozygosity may have important implications for the severe inbreeding depression observed in this crop.

Comparative genomics analysis revealed a considerable amount of genome diversity (SNVs and InDels) in W14, KU50 and CAS36 when compared with the reference genome of AM560. We identified 6.9 SNVs and 0.8 InDels per kb in W14, whereas 0.7 SNVs and 0.08 InDels per kb in the KU50. The number of SNVs discovered by comparison between the W14, KU50, CAS36 and AM560 genomes ranged from 2.84 to 4.81 millions (Supplementary Note 15, Supplementary Tables 4, 10, 11 and 12). Of these, 570,695 were shared by the genomes of wild and cultivated varieties, and 200,908 were found in genic regions. These SNVs constitute valuable markers for genotyping, genetic analysis and genomics-based breeding in cassava (Fig. 1a, Supplementary Note 12).

A total of 63 microRNA (miRNA) families consisting of 147 miRNAs were identified in the cassava genome, including 22 previously reported^32^ (Supplementary Note 21 and Supplementary Data 7). Other noncoding RNAs, including transfer RNAs (tRNAs, 861 in W14 and 707 in KU50), ribosomal RNAs (rRNAs, including 18S, 26S, 5.8S and 5S; 337 in W14 and 192 in KU50), small nuclear RNAs, small nucleolar RNAs, signal recognition particle RNAs and long noncoding RNAs, were also found in the wild and cultivated cassava genomes (Supplementary Note 21, Supplementary Table 21).

### Genome variation

Alignment of the larger scaffolds revealed that there is a significant similarity among the three cassava genomes, as expected, more substantial syntenic blocks are found between *M. esculenta* and *Ricinus communis* than between *M. esculenta* and *Arabidopsis thaliana* (Fig. 1c). Of the 15,636 gene families identified in *M. esculenta*, 2,043 were present in cassava but absent in other sequenced Euphorbiaceae genomes (*R. communis* and *Jatropha curcas*) or the outgroup species *(Vitis vinifera*; Supplementary Note 13, Supplementary Fig. 13). Further gene model comparisons among cassava and 12 more distantly related genomes revealed that 8,414 gene models were unique to cassava and 3,710 were specific to Euphorbiaceae (Supplementary Note 13, Supplementary Figs 14 and 15). Using 71 chloroplast genes from eight different plant species (Fig. 1d), we estimated that cassava diverged from rubber tree (*Hevea braziliensis*) 5.1 million years ago (MYA), 6.4 MYA from physic nut (*J. curcas*) and 14.8 MYA from castor bean (*R. communis*). Therefore, the cassava lineage from which cultivated cassava was originated diverged from a common ancestor approximately 0.5 MYA. These results are in agreement with the reported divergence time between *Manihot* and other genera in the Euphorbiaceae family^33^,^34^ (Supplementary Note 13).

We compared all the predicted genes from the genomes of W14, KU50 and AM560. A total of 28,302 independent gene models were confirmed, although copy number variations existed and were more frequently observed in the cultivated varieties than in W14 (Supplementary Data 1, Supplementary 16). Among the gene models, 1,584 were unique to W14 or lost in KU50 and AM560, whereas another 1,678 genes were specific to the cultivated varieties, and 20,133 homologous genes (including 16,219 high-confidence orthologues) were shared among the three draft genome sequences (Supplementary Note 14). The majority of the present and absent variation genes could be assigned to six Gene Ontology (GO) functional categories, including ‘catalytic activity’, ‘binding’, ‘metabolic process’, ‘cellular process’, ‘cell’ and ‘cell part’, and those genes with significant copy number variation were mainly ascribed into the first three functional categories (Supplementary Figs 17–19 and 41–43). Significant differences in the average SNVs were detected between W14 and the cultivated varieties (1.7%) and between the two cultivars (0.5%; Supplementary Note 16, Supplementary Fig. 24).

**Figure 2:**
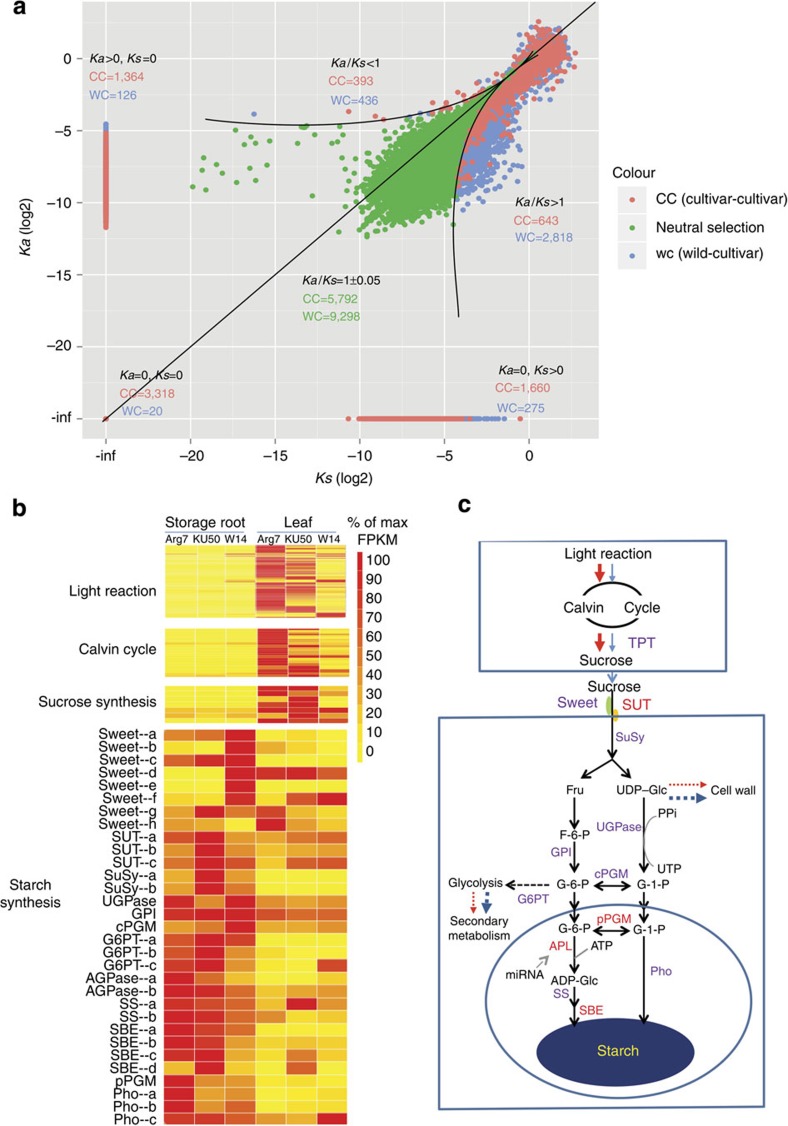
Selection pressure and carbon flux diversification in cassava. (**a**) Chart for synonymous substitution (*Ks*) and nonsynonymous substitution rate (*Ka*) and selection pressure (*Ka/Ks*) between wild W14 and cultivated variety (WC) and between cultivated varieties (CC). *Ka/Ks*=1 indicates genes with neutral selection, *Ka/Ks*>1 indicates positive selection and *Ka/Ks*<1 indicates negative selection. (**b**) The differential expression patterns of genes involved in photosynthesis, Calvin cycle, sugar transport and starch synthesis in storage roots and leaves between cultivated varieties (KU50 and Arg7) and wild ancestor (W14) revealed by digital transcriptome sequencing. (**c**) A model of high-efficient starch accumulation in the tuber roots of domesticated cassava. Red arrows present the carbon flux directions in cultivar and blue arrows indicate the carbon flux directions in wild W14. The width of the arrow indicates the strength of carbon flux. The gene symbol marked in red shows genes with copy number expansion in cultivars. cPGM, cytoplasmic phosphor-glucomutase; GPI, glucose-6-phosphate isomerase; G6PT, glucose-6-phosphate/phosphate translocator; pPGM, phospho-glucomutase; SBE, starch branching enzyme; SS, starch synthase; SUT, sucrose transporter; TPT, triosephosphate translocator; UTP, uridine triphosphate.

The synonymous (*Ks*) and nonsynonymous substitution rate (*Ka*) and selection pressure (*Ka/Ks*) of the gene set were used to describe evolutionary signatures of the cassava genome^35^,^36^ (Supplementary Note 16, Supplementary Fig. 26). Approximately 2,818 genes were strictly positively selected (Fig. 2a, *Ka*/*Ks*>1), 436 genes were negatively selected (Fig. 2a, *Ka*/*Ks*<1) and 9,298 genes were selection-neutral (Fig. 2a, *Ka*/*Ks*=1) during evolution of cultivated varieties, whereas 6,342 genes exhibited lack of neutral or selected divergence between cultivars (Fig. 2a, *Ka*=*Ks*=0, *Ka*=0, *Ks*>0 and *Ka*>0, *Ks*=0) (Supplementary Tables 13, 14 and 15). By comparison, we found that 1,133 genes have been heavily selected in the domesticated cultivar, indicating a selective sweep. Analyses of GO functional categories indicated that those genes were mainly enriched in four categories: (i) ‘developmental process’ including cell differentiation and organ development such as leaf, stem, storage root and fruit; (ii) ‘metabolic process’ centred around cell wall polysaccharide synthesis, secondary metabolites and fatty acid metabolism; (iii) ‘biological regulation’ involved in regulation of cell size, cellular metabolism, immune and transcription; (iv) ‘response to stimulus’ including abiotic stresses such as light, temperature, water and oxygen, and biotic stresses caused by viral, bacterial and fungal, and response to hormones such as abscisic acid, ethylene, jasmonic acid and brassinosteroids (Supplementary Fig. 27). The enrichments in such GO categories suggested that those genes that underwent selection cover nearly every aspect of phenotypic variations necessary for cassava cultivation.

Comparative transcriptome analysis between W14 and cultivated varieties in developing leaf and storage root revealed additional specific features related to the evolution of the cassava varieties. From the total of 31,396 genes expressed in W14 or cultivated varieties, 749 show significantly differential expression in leaves and 2,732 in storage roots (Supplementary Note 17, Supplementary Fig. 28, Supplementary Table 16). GO analysis of those genes revealed an enrichment in genes involved in specific metabolic pathways in the wild and cultivated varieties. In leaves, the cultivated varieties show a particular transcript enrichment in genes involved in ‘photosynthesis’ and shaping the photosynthetic organelles. Interestingly, genes belonging to the GO category of ‘response to stimulus’, including abiotic and biotic stresses are also enriched in the cultivated varieties (Supplementary Note 17, Supplementary Fig. 30). In contrast, genes involved in ‘transporter activity’, including a potassium symporter and a calcium transporting ATPase, are enriched in the wild W14. The category ‘positive regulation of flower’ was specifically enriched in W14, which is consistent with the fact that sexual reproduction is more frequent in wild than in cultivated cassava. In storage roots, genes included in the categories ‘cell part’ (specially subcategories of ‘cytoplast’ and ‘plasmid organelle’) and ‘response to stimulus’ (particularly abscisic acid, oxidative stress and temperature) were only enriched in cultivated varieties. However, genes within the categories ‘cell wall polysaccharide biosynthesis process’, ‘secondary metabolic process’ and ‘response to stimulus’ (such as water stress and jasmonic acid) were enriched in the wild species (Supplementary Note 23, Supplementary Fig. 29, Supplementary Table 17). These enriched GO categories in the transcriptomes of wild and cultivated varieties were consistent with the functions of the genes found to be under higher selection pressure (*Ka/Ks*). Further statistical analysis revealed that the Ka/Ks ratios of genes belonging to GO categories enriched in the cultivated varieties were higher than those from categories in the W14 wild species (Supplementary Fig. 31). Those genes have been restrictedly selected and differentially expressed between wild and cultivars, probably geared the ecological changes resulting a shift in growth environment from rainforest to cerrados. This is consistent with the observed variation of the phenotypes of domesticated cassava.

### Carbon flux diversification

The high carbon accumulation in the form of starch in the storage root is an extraordinary feature of cultivated cassava. The transcriptome annotation showed that a considerable number of genes involved in photosynthesis and the Calvin cycle in leaves, and sucrose transport and starch synthesis in storage roots were preferentially expressed in the two domesticated varieties when compared with the wild W14 (Fig. 2b). This is consistent with the higher vigour and yield potential showed by KU50 and Arg7 relative to W14 (Supplementary Table 1, Supplementary Fig. 1). These results were confirmed by reverse transcriptase–quantitative PCR of selected genes (Supplementary Note 20, Supplementary Fig. 34). Also, we found an alternative starch synthesis pathway relying on plastid phosphorylase (*Pho1*), which was expressed at a higher level in cultivated varieties than in W14 in the storage roots. This pathway allows glucose 1-phosphate to be directly transferred into amyloplasts, as shown in rice grain^37^ and potato tubers^38^. The expression level of genes involved in cell wall synthesis and secondary metabolism are significantly decreased in leaves and storage roots of cultivated varieties in comparison to the wild subspecies (Supplementary Note 18, Supplementary Figs 32–33). These results agree with the observation that SWEET genes controlling sucrose efflux into the cell wall show reduced expression in KU50 and Arg7, but not in W14. At the genome level, copy number expansion and alternative splicing were found in several key genes in the cultivars, such as aldolase, phosphoglycerate kinase and ribulose bisphosphate carboxylase, which are involved in photosynthesis. Genes involved in starch synthesis and accumulation in amyloplasts of storage roots such as sucrose transporters, sucrose synthases, ADP glucose pyrophosphorylase (APL), starch branching enzymes and phospho-glucomutase showed similar copy number and alternative splicing differences (Supplementary Data 5). These genes have been identified as the key genes strongly associated with cassava storage root development^39^. Furthermore, our result suggests that miRNAs may play a role in regulating storage root formation and growth as well as starch synthesis. At least nine miRNAs could target genes that were highly expressed in the storage roots of cultivars and were involved in the photosynthesis and carbon metabolism pathways (Supplementary Note 21, Supplementary Table 19 and Supplementary Fig. 36) as observed in other plants. For example, miR394 directly downregulates APL2, a key gene in starch synthesis at the late stage of storage root development in cultivated varieties; likewise miR319, miR159, miR160, miR166 and miR396 negatively regulate their targets, such as MYB33 and ARF10, which control starch synthesis through ABA signalling^40^,^41^ (Supplementary Data 9). MiR167, miR169 and miR156 positively regulate transcription factors RD19, NF-YA3 and SPL13B, respectively, which are involved in storage root and leaf development^42^,^43^,^44^. Remarkably, target genes such as MYB33 ARF10 and NF-YA3 are known to bind to *cis*-elements in the upstream regions of genes related to starch metabolism, such as SuSy, APL and genes involved in photosynthesis (Supplementary Note 22, Supplementary Figs 35 and 37–40). Taken together, the observed gene expression patterns, enhanced agronomic phenotypes, copy number expansions and miRNA- and *cis*-element-mediated regulation of key genes suggest that carbon flux could have been shifted as a result of domestication in cassava. Based on these findings, we suggest a model of the efficient accumulation of starch in cassava (Fig. 2c)^45^.

### Cyanogenesis differentiation

**Figure 3:**
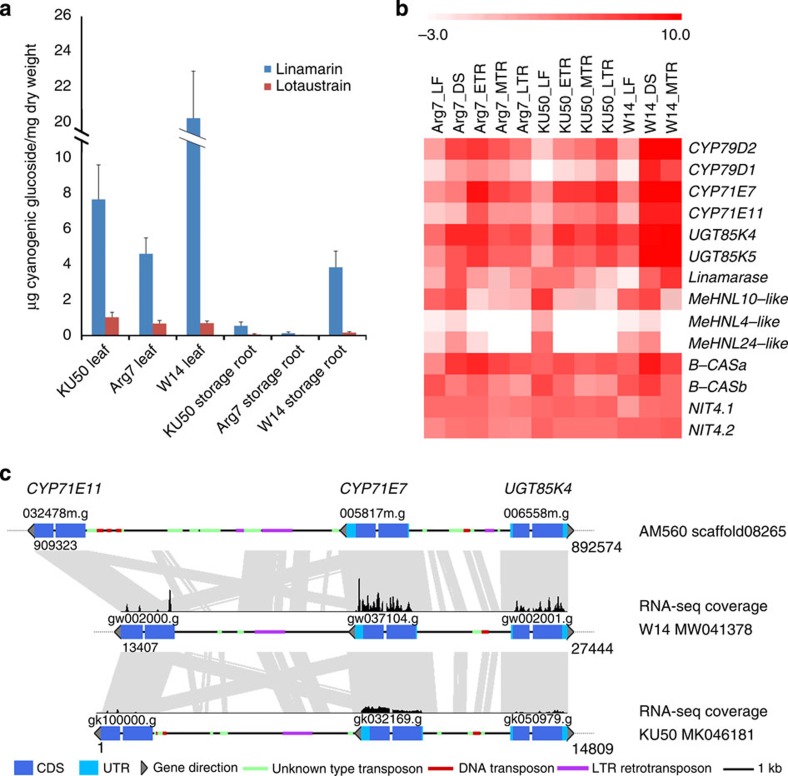
Cyanogenesis differentiation between wild and cultivated cassava. (**a**) Minimizing of cyanogenic glucoside content in cultivar KU50 and Arg7 relative to wild W14: over twofold in leaves and fivefold in storage root with five repeat plants. (**b**) Differential expression of genes in the cyanogenic glucoside synthesis pathway between cultivar KU50, Arg7 and wild W14 identified by RNA-seq. DS, developing stem; ETR, early storage root; LF, leaf; LTR, late storage root; MTR, medium tuber root. (**c**) A transposon regulation model of cyanogenesis in cassava: among the interval regions of three genes in a linear array as *CYP71E11*, *CYP71E7* and *UGT85K4*, there were more transposable or retrotransposable elements in the gene 1-kb upstream regions of cultivated species KU50 and AM560 than wild subspecies W14. CDS, Coding sequence.

The latent toxicity caused by cyanogenesis in cassava is clearly a potential health hazard when it is consumed as food. The pathway for cyanogenic glucoside biosynthesis in cassava and the genes encoding the enzymes involved have been elucidated in recent years^46^,^47^,^48^. We determined the linamarin and lotaustralin content in cultivated KU50, Arg7 and wild W14, and found that the linamarin content was reduced six- to tenfold in storage roots and three- to fourfold in leaves of KU50 and Arg7 relative to W14 (Fig. 3a, Supplementary Note 19, Supplementary Table 18). Remarkably, the expression of the genes *CYP79D1, CYP79D2*, *CYP71E7, CYP71E11, UGT85K4* and *UGT85K5* that encode the enzymes catalysing linamarin and lotaustralin formation, all exhibited five- to tenfold lower expression levels in the storage roots and leaves of KU50 relative to W14, further suggesting a potential outcome of domestication. Different classes of DNA retrotransposons, like miniature inverted-repeat transposable elements (MITEs) and LTR transposable elements, have been shown to influence the expression of proximal genes, especially if simultaneously situated downstream and upstream of the same gene. In general, gene expression is suppressed by the presence of these elements^48^. To investigate potential effects of transposons on gene expression in cassava, the 1-kb upstream regions of orthologous genes present in the W14, KU50 and AM560 genomes were analysed for the presence of MITEs. A total of 553 MITEs were found, of which 310 and 243 were uniquely present within the genomes of AM560 and W14, respectively. Among the 310 AM560-specific MITE insertions, 96 (34.5%) showed significantly lower expression and 32 (11.5%) had significantly higher expressions in storage roots or leaves of cultivated varieties when compared with W14 (Supplementary Data 6). We compared the genomic regions containing *CYP71E11, CYP71E7* and *UGT85K4*, and found that these three genes were positioned in a linear array within homologous scaffolds in the three genomes. Two distinct larger insertions containing MITE and LTR transposons were identified to be present in the 5′UTR and 3′UTR regions of those genes in KU50 and AM560, but not in the wild ancestor W14 (Fig. 3c). Taken together, these results suggest that transposon activity may have played a role in the reduction of cyanogenic glucoside content in the domesticated cassava. It remains to be seen how the distribution patterns of transposable elements affect cyanogenic compound biosynthesis in cassava, although transposable elements have been shown to alter the expression patterns of adjacent genes in plant genomes^49^.

## Discussion

We produced and annotated two draft genomes of cassava, a cultivated variety and a wild ancestor. Comparative analysis provided new insights into cassava genome evolution and genetic events that may have occurred during domestication. Gene models specific to either wild or cultivated cassava were elucidated. We found a high degree of heterozygosity between the analysed cassava genomes and gene sets that have been strictly selected during the process of evolution and, potentially, domestication. Genes responding to stimulus such as light, high temperature, water stress and oxidative stress were highly expressed in domesticated cassava, most likely reflecting their adaptation to tropical and dryer growth conditions. On the other hand, some genes involved in ion membrane transport were lost, suggesting that wild cassava ancestors were more tolerant to extreme environments than the current cultivated varieties. Particularly, two parallel but contrasting selection trends were identified in the domesticated cultivars, one leading to an increase in major carbon metabolism pathways, photosynthesis, sugar transport and starch metabolism that could enhance starch yield potential, and the other leading to a dwindling of cell wall and secondary metabolism, including cyanogenic compounds. This carbon flux shift towards starch accumulation would be desirable in cultivated varieties, whereas stress tolerance may not be so critical in cultivation conditions, as biotic and abiotic stress can be milder than in natural environments. Therefore, we propose that a pathway that prioritizes starch accumulation versus cyanogenesis has been selected in cultivated cassava. In addition, the development of substantial new genomic resources, including millions of SNVs, which are available in a public database (http://www.cassava-genome.cn/), will promote development of toolkits for enhanced cassava breeding.

## Methods

### Genomic DNA isolation

To reduce organelle contamination in genomic DNA, nuclei were isolated from fresh young leaves of W14 and KU50, as described by Zhang *et al*.^50^ Briefly, approximately 100 g of tissues were ground into a fine powder in liquid nitrogen and transferred to a beaker containing ice-cold 1 × homogenization buffer plus 0.5% Triton X-100 and 0.15% β-mercaptoethanol. After filtering the homogenate through cheesecloth and Miracloth, the nuclei were washed with the same buffer and centrifuged. This step was repeated until the nuclei pellet became white. DNA was extracted from the nuclei as described by Kidwell and Osborn^51^. The purified DNA was dissolved in 1 ml of TE-buffer for Illumina and 454 sequencing.

### Genome sequencing

Genome sequence data of W14 and KU50 were produced with the Illumina HiSeq2000 (Illumina) and Roche/454 GS FLX platforms (Roche) at the Beijing Institute of Genomics and Qingdao Bioenergy and Process Institute of the Chinese Academy of Sciences. A total of 76.32 Gb high-quality DNA sequence for W14 and 34.43 Gb for KU50 representing 103- and 46-fold coverage of the 742-Mb cassava genome, respectively, were generated.

### BAC library and physic mapping

The BAC libraries of wild W14 and cultivated cassava inbred line AM560-2 were constructed used for integrated assembly of genome W14 and KU50, respectively. For W14, *Eco*RI- and *Hin*dIII-digested DNA fragments were cloned into pCC1BAC vector and the BAC libraries were constructed by Amplicon Express Inc., using the method of Tao *et al*.^52^ Total of 59,904 clones with the average insert size of 115 kb for *Eco*RI and 129 kb for *Hin*dIII, respectively, were acquired and represent approximately ten genome equivalents. For AM560-2, the DNA fragments were cloned into the *Hin*dIII site of the vector pIndigoBAC536, and transformed into the *E. coli* host DH10B. A total of 72,192 clones were obtained with an average insert size of 115 kb and approximately 7% clones with no insert. The library represents about 11 × coverage of cassava genome size of 746 Mb estimated (Supplementary Note 2, Supplementary Table 2). BAC clones were fingerprinted with a SNaPshot high-information content fingerprinting method described by Luo *et al*.^53^,^54^, and modified by Gu *et al*.^55^ The GeneMapper software was used for fragment size-calling. Outputs of size-calling files were automatically edited with the FP Miner programme. A total of 72,192 BAC clones from the cassava AM560-2 library and 29,952 clones from W14 library were fingerprinted, 58,244 clones of AM560-2 and 24,784 clones of W14 were suitable for fingerprinted contigs (FPC) assembly, respectively, resulted in a total of 2,105 contigs and 5,054 singletons in AM560-2 and a total of 2,485 contigs and 2,909 singletons in W14 (Supplementary Table 2).

### Genome assembly and annotation

The long sequence of 454 libraries assembly was generated using Newbler version 2.5.3 with default parameters, and short sequence of illumina libraries assembly was generated using SOAP denovo version 1.05 with default parameters, artificial sequences and poor quality bases had been trimmed before input for assembly process. Then by using BLAST version 2.2.25 with *E*-value parameter as 1e-5 to filtered the repeat contigs, and using Phrap version 1.080812 to merge the contigs with overlap–layout–consensus relationship. Using Fan’s link scaffold build bridge from contigs to scaffolds with illumina hierarchical insert span paired-ends and mate-pairs. The mega scaffolds were integrated by the scaffolds and BAC paired-ends sequence^56^,^57^, the connection between scaffolds and BAC paired-ends were ordered by FPC map^58^ using GATE v1.0 (https://github.com/BENMFeng/GATE/). Scaffolds within one pseudomolecule were interacted by a stretch of 500 undefined bases (N's). Annotation was carried out following the pipeline of GACP v7.0 (http://bioinformatics.genomics.org.cn/bio/annotation.html/) that combines the approach of the *ab initio* gene prediction on the repeat-masked genome, via protein similarity and transcript reconstruction to build optimal gene models using the PASA and EVM pipelines^59^,^60^. All genome assembly and annotation data of W14 and KU50 could been found at http://www.cassava-genome.cn/data.html with visionV1.0.

### RNA-Seq and annotation

The RNA-Seq reads were *de novo* assembled by Trinity^61^ and Velvet-Oases^62^,^63^, and those RNA-Seq reads and their assembled transcripts were mapped to the W14, KU50 and AM560 draft sequences using Tophat and BLAT. And the *ab inito* alignment to itself assembly draft genome using TopHat v2.0.6 and cufflinks v2.0.2, statistical analysis was completed with cummeRbund v1.2.0 to assist. And the whole-gene and genome annotated to nucleotide collection in NCBI database (NT), non-reduntant (NR), Kyoto Encyclopedia of Genes and Genomes (KEGG), GO, Clusters of Orthologous Groups of proteins (COG), Trembl, SwissPort were using GACP v2.0 and GATE v1.0. The expression levels and differential expression profiling of representative transcripts were calculated using Cufflinks^64^ and cuffdiff2 (ref. 65; Supplementary Note 17).

### MicroRNA and noncoding RNA annotation

The small RNA-seq data of W14 and KU50 were analysed to identify novel miRNAs and profile miRNA expression following the method previously documented^66^. The qualified reads, the ones that carried the adaptor and were longer than 17-nt, were mapped to the known miRNAs to detect their expression and determine their expression abundance. The qualified reads not mapped to the known miRNAs were mapped to a cassava genome to identify novel miRNAs. The conservation and specificity of all newly identified and known cassava miRNAs were analysed across the cassava genotypes and eight diverse plant species. The sequences of these miRNAs were aligned to the genomes of the cassava cultivars and the other eight plants using BLAST with the *P*-value threshold set to 1e-10; the alignment results were further manually examined to determine homologue to a miRNA.

The other noncoding RNA genes were analysed using existing tools. In particular, tRNAs were analyzed using tRNASCAN-SE^67^ (Version 1.23); rRNAs were identified by RepeatMasker (Version open 3.3.0) with cloned 18S, 5.8S, 26S and 5S rDNA sequences of full-length KU50 as the library; and the other types of RNAs were detected by INFERNAL^68^ (version 1.1) with cm models downloaded from Rfam database (Version 11.0).

### Repeat analysis

Repetitive sequences were identified *de novo* from the genome assemblies of W14 and KU50 using RepeatModeler Open-1.0 pipeline (Version 1.0.5, http://www.repeatmasker.org). Parameters were used following the software pipeline by default. The identified consensus sequences were classified using Viridiplantae repeats from RepBase and used to mask the assembly sequences with RepeatMasker open-3.3.0 (http://www.repeatmasker.org). Divergence rate was measured by the percentage of substitutions in the well-aligned regions between annotated repeats in the cassava genome and the consensus repetitive sequences identified *de novo* as above.

### Gene family analysis in Euphorbiaceae

BlastP was used on all the protein sequences against a database containing a protein data set of *M. esculenta*, *Jatropha curcas* (Barbodos Nut), *Ricinus communis* (castor bean), *Arabidopsis* and *Vitis vinifera* (grape) under an *E*-value of 1E-5.The OrthoMCL method with mode 3 was applied to construct gene families.

### Cyanogen analysis

The cyanogenic glucoside contents of roots and leaves of the wild W14 and cultivated KU50 was determined by liquid chromatography–mass spectrometry. Five plants were analysed separately for each of them. A leaf disc was sampled from the first unfolded leaf of each plant by snap-closing the 2-ml-Eppendorf lid tubes. The plant samples were immersed into 300 μl and 500 μl of pre-warmed 85% (v/v) methanol for leaf and tuber, respectively. After closing the tube and securing the lid with a cap lock, the samples were boiled in a water bath at 100 °C for 3 min (leaf) or 5 min (tuber). Then, the MeOH extract was transferred into a new tube, lyophilized to dryness, re-suspended in water in a total volume of 200 μl and filtered through a 0.45-μm filter. Analytical liquid chromatography–mass spectrometry was carried out using an Agilent 1100 Series LC (Agilent Technologies).

## Author contributions

W.W., M.P. conceived the project, designed the studies and contributed to the original concept of the manuscript. J.Xiao, K. Li and B. Liu analyzed the data as a whole and wrote the manuscript. B. Feng developed the *de novo* assembly pipeline and performed *de novo* genome assembly. X. Chen, Y. Zhang, K. Pan and Q. Yao performed DNA preparation. Z. Xia, P. Li, S. Zhong, J. Liu and J. Zhang performed transcriptome (RNA-seq and cDNA) analyses. X. Zhou, G. Wu, P. Ling and J. Guo performed the repetitive sequence analysis and tRNA, rRNA, small nucleolar RNA annotation. Z. Li, M. Hu, S. Wang, W. Liao, W. Hu, S. Zhang, M. Zou, M. Wen, J. Pei, P. Ma and M. Ruan completed the Q-PCR validation of selected genes. M.C. Luo, P. Rabinowicz, J. Wu, Y. Ma, X. Liu, L. Tallon, K. Galens, S. Ott, F. You and Y. Fu performed construction of BAC libraries and physic map. H. Zhang and Y. Fu produced part of BAC clones used for BAC pooling sequencing and H. Zhang also contributed for edition of in language. Q. Lou, H. Wang, C. Lu and A. Guo performed cytogenetics studies. S. Zhou, S. Hu, Z. Wu, H. Liu, S. Sun provided IT support. G. Liu, Y. Chen and Q. Wang coordinated the project. Y. Wang, G. Xiao, L.J. Carvalho and S. Chen performed the comparative genomics analyses across the species. W. Zhang, J. Xia and C. Zeng completed the annotation of miRNAs. B. Møller, R. Kannangara, K. Jørgensen performed the analysis of cyanide metabolism and contributed to the writing of the manuscript. H. Ceballos, M. Fregene, L.A.B. López-Lavalle, R. Neale, N. Heinz, M. Bonde and P Zhang. gave the revision of manuscript.

## Additional information

**How to cite this article**: Wang, W. *et al*. Cassava genome from a wild ancestor to cultivated varieties. *Nat. Commun.* 5:5110 doi: 10.1038/ncomms6110 (2014).

**Accession numbers**: Cassava genome sequence data have been deposited at DDBJ/EMBL/GenBank under the accession code JPQE00000000 for W14 and JPQF00000000 for KU50. RNA-seq reads have been deposited in GenBank/EMBL/DDBJ sequence read archive under the accession codes SRX551093, SRX553797, SRX553798, SRX553799, SRX553800, SRX553801, SRX553802, SRX553803, SRX553804, SRX553805, SRX553806 and SRX553807.

## Supplementary Material

Supplementary Figures, Supplementary Tables, Supplementary Notes and Supplementary References Supplementary Figures 1-43, Supplementary Tables 1-21, Supplementary Notes 1-23 and Supplementary References

Supplementary Data 1 Integrated gene models and their copy number variation (CNV) among AM560, KU50 and W14.

Supplementary Data 2 Structural variation and expression profiling of genes for light reaction pathway.

Supplementary Data 3 Structural variation and expression profiling of genes involved in Calvin cycle.

Supplementary Data 4 Structural variation and expression profiling of genes involved in Sucrose synthesis.

Supplementary Data 5 Structural variation and expression profiling of genes involved in starch biosynthesis.

Supplementary Data 6 Comparative expression profiling of genes with MITEs insertion in promoter region of cultivar.

Supplementary Data 7 Genomic loci of 147 miRNAs in the three Cassava genomes.

Supplementary Data 8 Normalized digital read counts for each miRNA in different tissues and cultivars.

Supplementary Data 9 Significantly different expression of 9 specific microRNAs and their corresponding targets in root of cultivars and wild subspecies.

Supplementary Data 10 FPKM values of target genes of all microRNAs in leaf and tuber root of KU50, Arg7 and W14.
