# Supplementary material for: Cassava genome from a wild ancestor to cultivated varieties
Source: Nat Commun. 2014 Oct 10;5:5110. doi: 10.1038/ncomms6110 (PMC4214410; doi:10.1038/ncomms6110)
Supplement: Supplementary Figures, Supplementary Tables, Supplementary Notes and Supplementary References — Supplementary Figures 1-43, Supplementary Tables 1-21, Supplementary Notes 1-23 and Supplementary References [file ncomms6110-s1.pdf]

Supplementary Information

1) Supplementary Figures

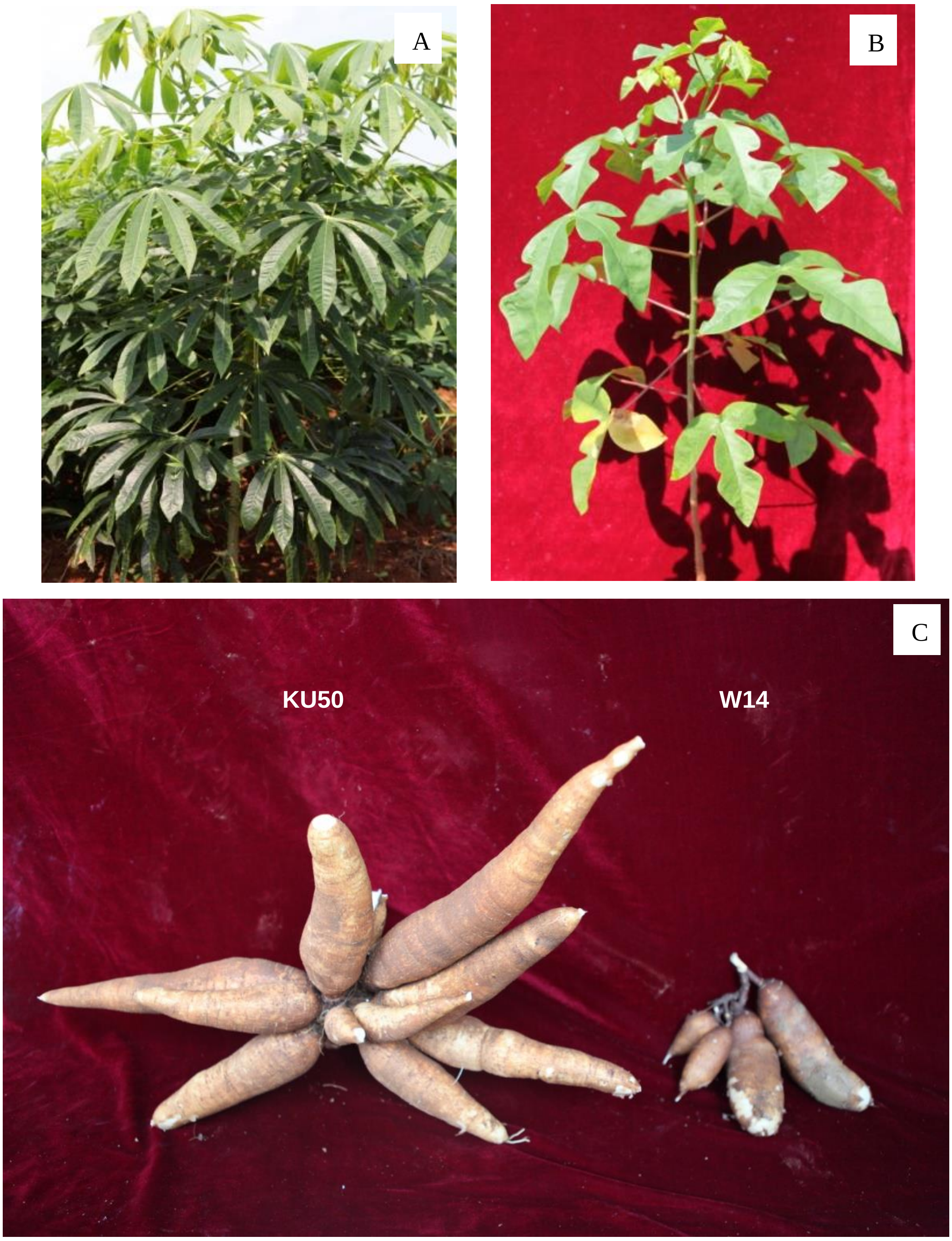

Supplementary Figure 1 Plants and tuber roots of cultivar KU50 and wild W14 growing for six months under field condition

(A) Plant of KU50. (B) Plant of W14. (C) Comparison of tuber roots between KU50 and W14. The tuber root of KU50 is much larger than that of W14, with an average yield of 5.8 kg/plant and 0.8 kg/plant, respectively, after growing for six months.

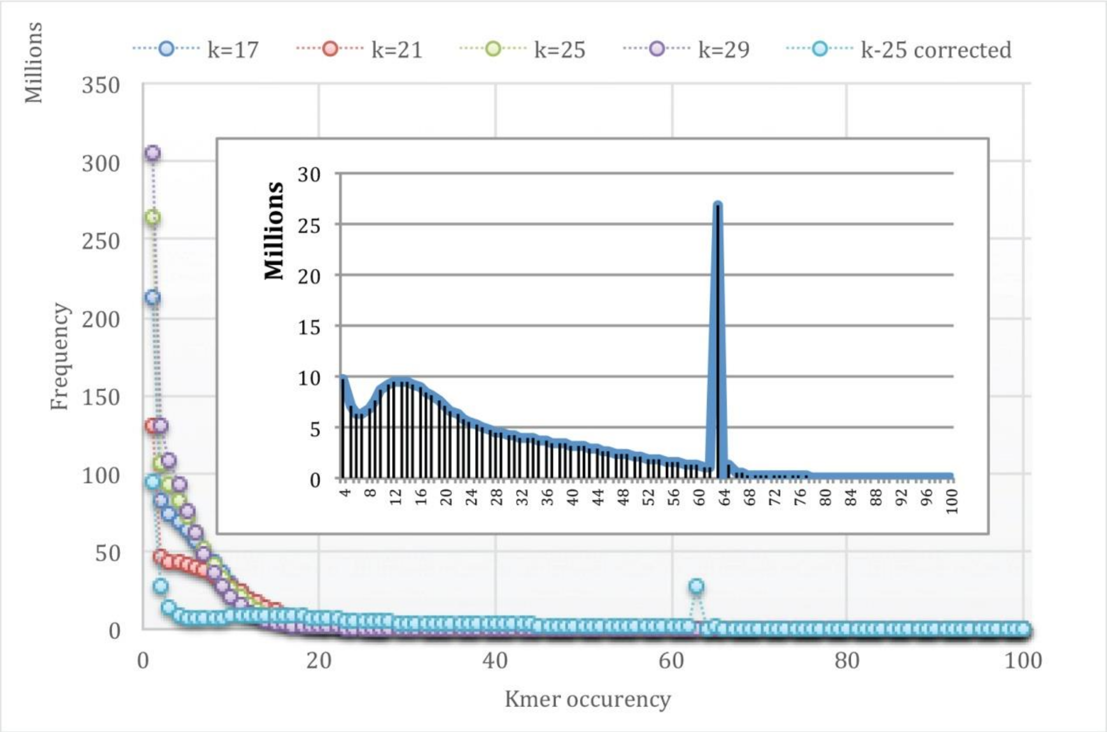

Supplementary Figure 2 Genome size estimation with multi-kmer frequency distribution of W14

The main graph depicts the distribution of 17mer, 21mer, 25mer, and 29mer in the reads of short insert size libraries (200-500 bp) and the inset shows the volume of 25mer corrected by the kmer spectrum method. The total kmer number of 'k=25 corrected' is 9,644,794,319, and the volume peak is 13, so the genome size can be estimated in 742 Mb using the formula: (total kmer number) / (the volume peak).

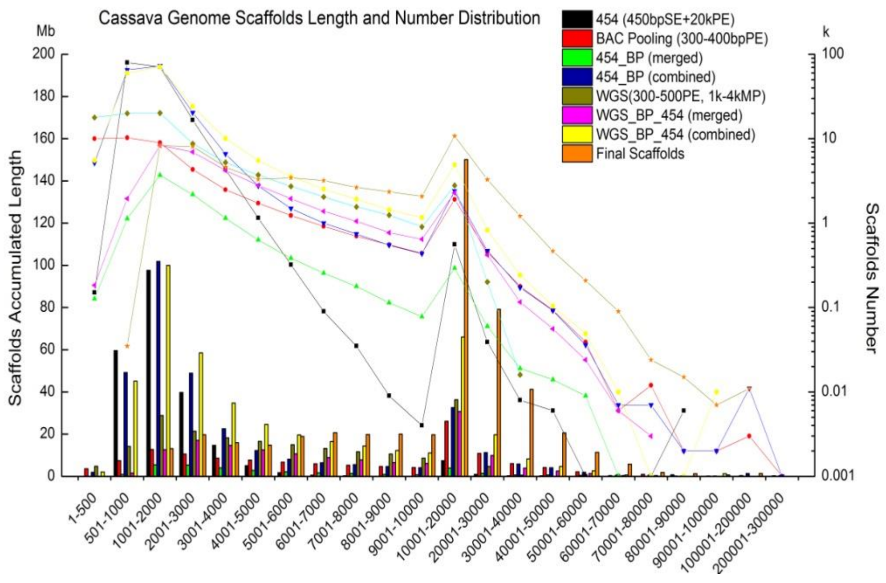

Supplementary Figure 3 Data contribution to hybrid assembly

The bar-chart was depicted contribution of scaffolds accumulated length consist of different types of sequencing data. The line-points were depicted contribution of different types of sequencing data in for constructed the scaffolds number.

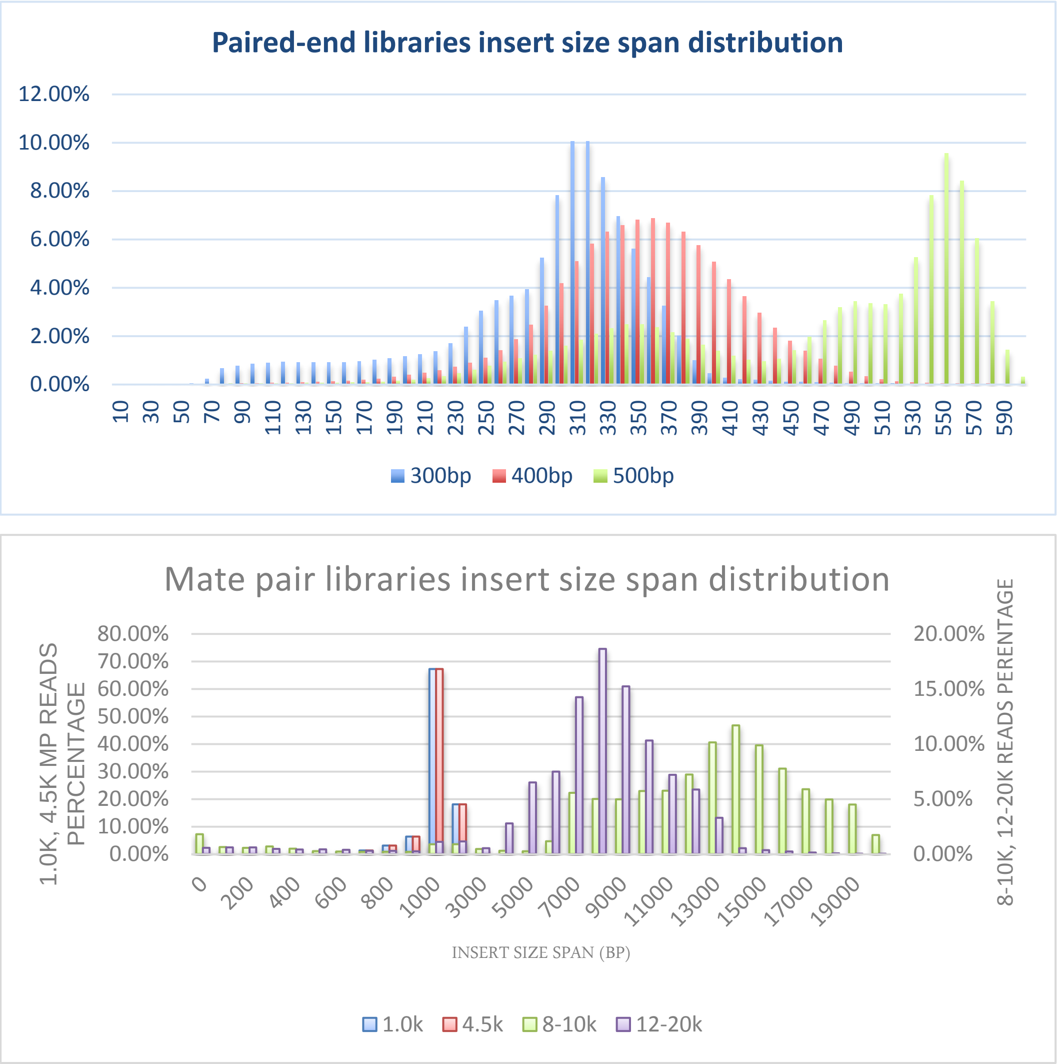

Supplementary Figure 4 Sequencing libraries insert size span distribution

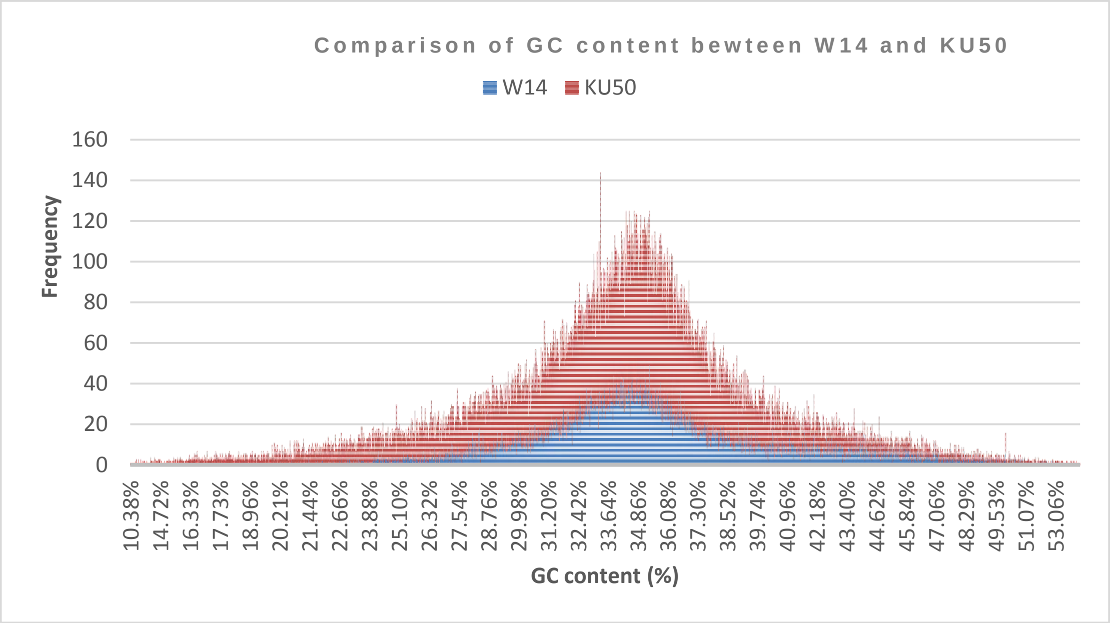

Supplementary Figure 5 The draft genome GC content distribution of W14 and KU50

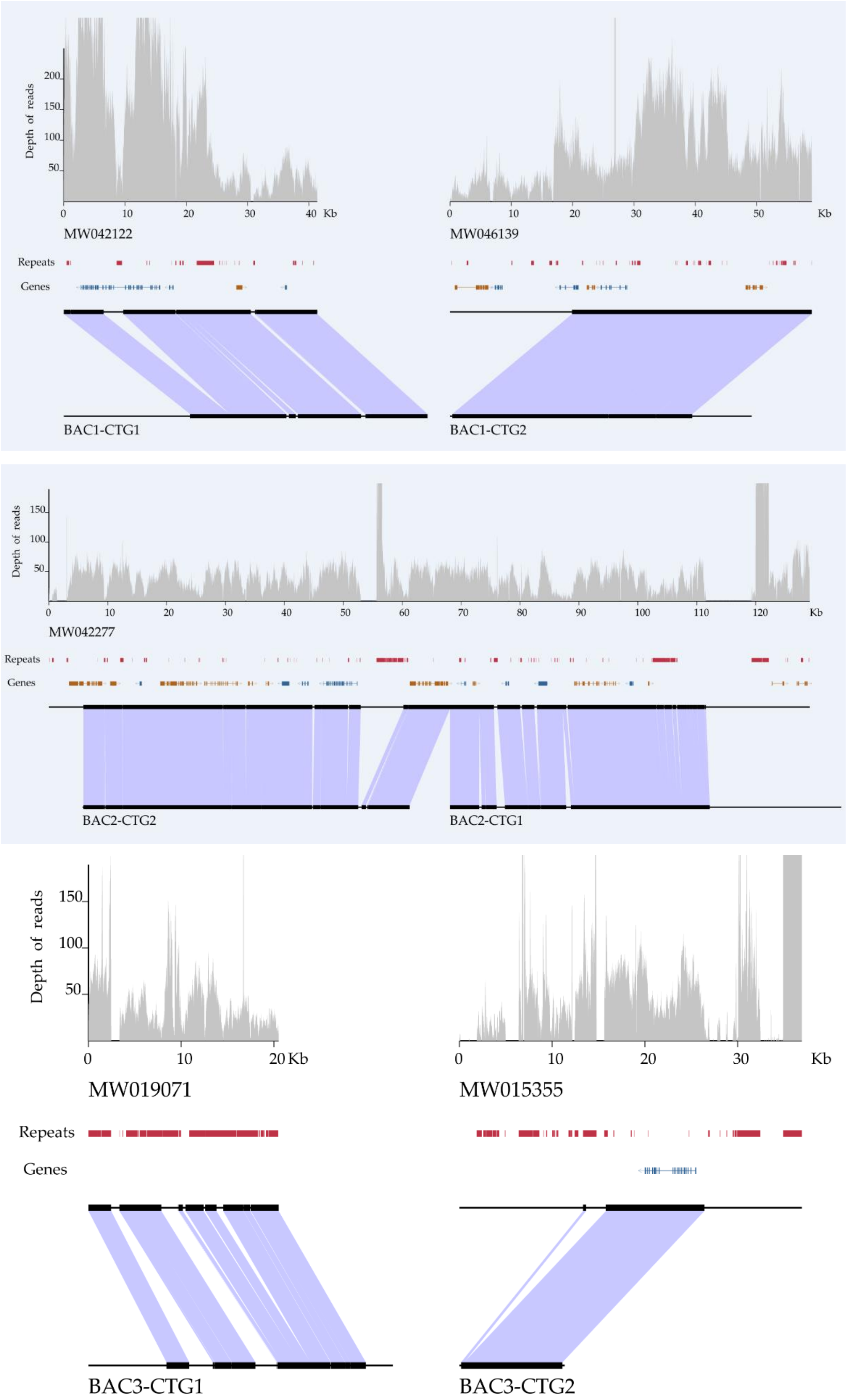
Depth of reads
200
150
100
50
0
10
20
30
40
Kb
MW042122
0
10
20
30
40
50
Kb
MW046139
Repeats
Genes
BAC1-CTG1
BAC1-CTG2
Depth of reads
150
100
50
0
10
20
30
40
50
60
70
80
90
100
110
120
Kb
MW042277
Repeats
Genes
BAC2-CTG2
BAC2-CTG1
Depth of reads
150
100
50
0
10
20 Kb
MW019071
0
10
20
30
Kb
MW015355
Repeats
Genes
BAC3-CTG1
BAC3-CTG2

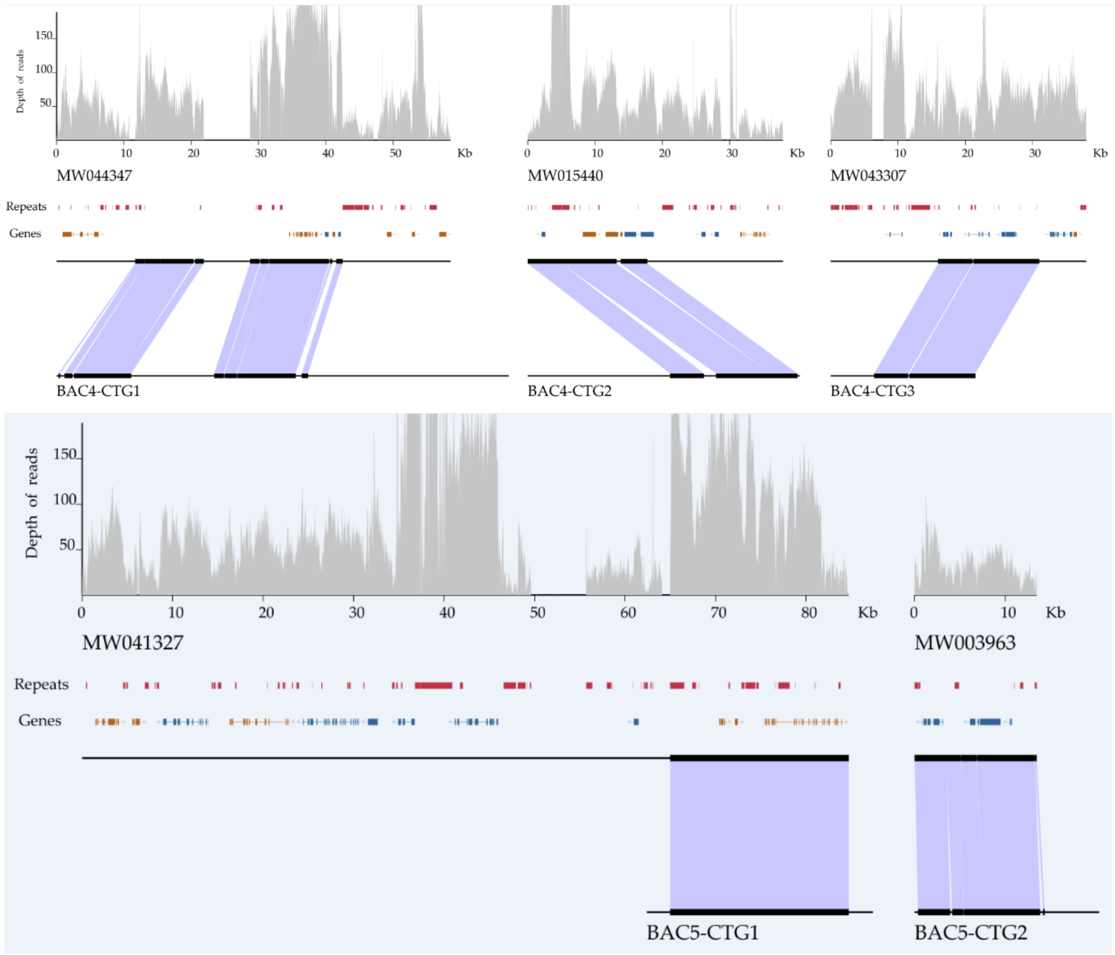

Supplementary Figure 6 Alignment of the assembled W14 scaffolds to the BACs sequenced by the 454 platform

Depth of reads in gray was calculated by mapping reads onto the W14 scaffolds. Repeats in red show the annotated TEs on the W14 scaffolds. The coffee and deep blue connected small blocks show the annotated genes on the W14 scaffolds. The thin black lines show the unmatched regions between the W14 scaffolds and BACs, while the thick lines show the matched regions, and the light blue blocks between them show the aligned region between 454 BACs and scaffolds. The alignment of other scaffolds to W14 genome have more or less the same trend with the above charts.

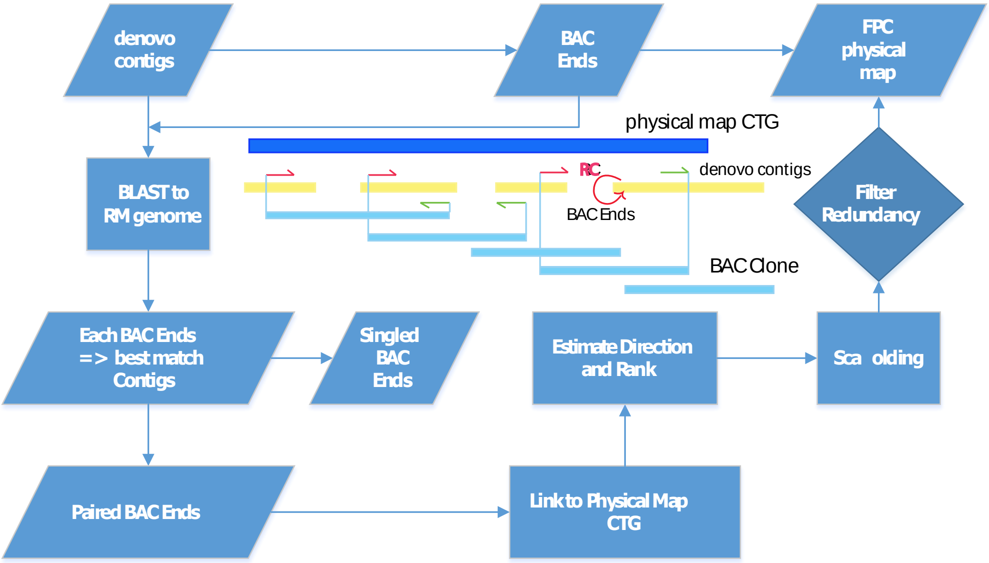

Supplementary Figure 7 Flowchart showing the pipeline of integrated scaffolding the physical map and draft genome

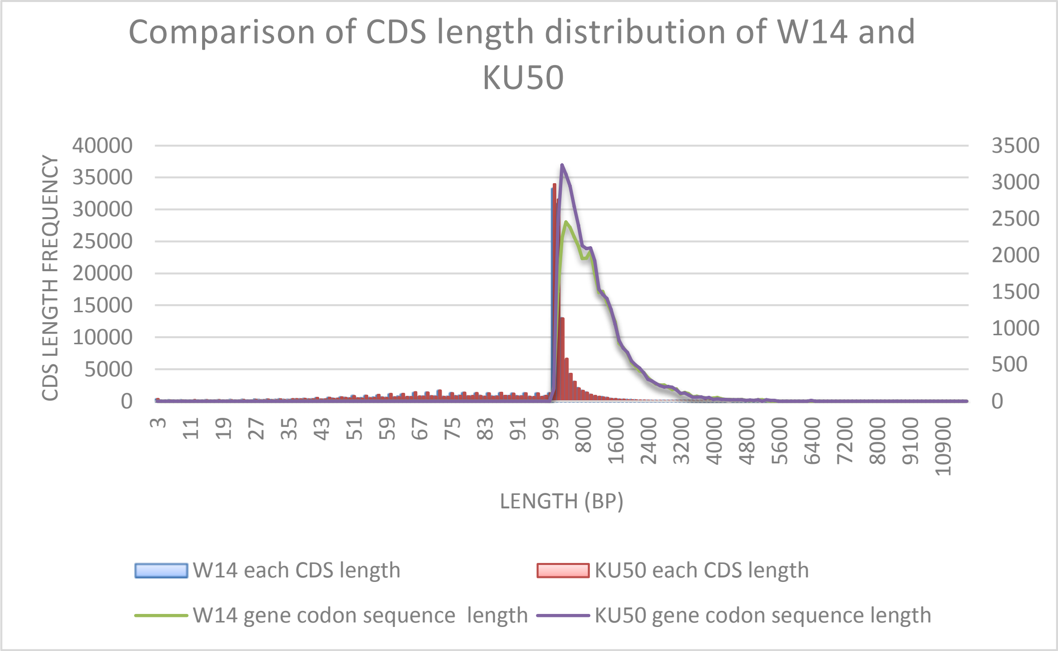

Supplementary Figure 8 Length distribution of CDS of all predicted genes in the genomes of W14 and KU50

a. **Cumulative Frequency of Cassava ESTs Mapped to the MW_v1 Genome**

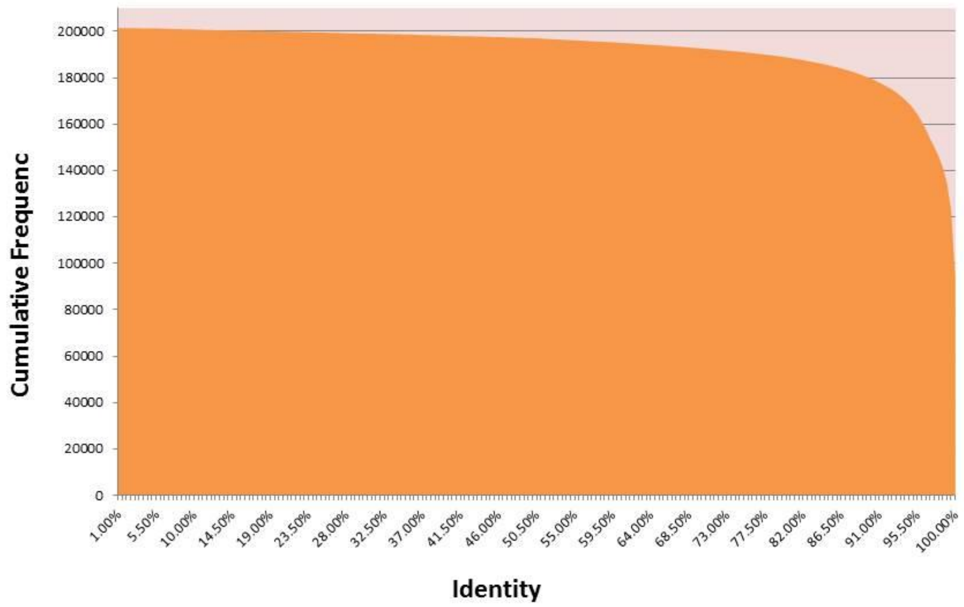

b. **Cumulative Frequency of Cassava ESTs Mapped to the MK_v1 Genome**

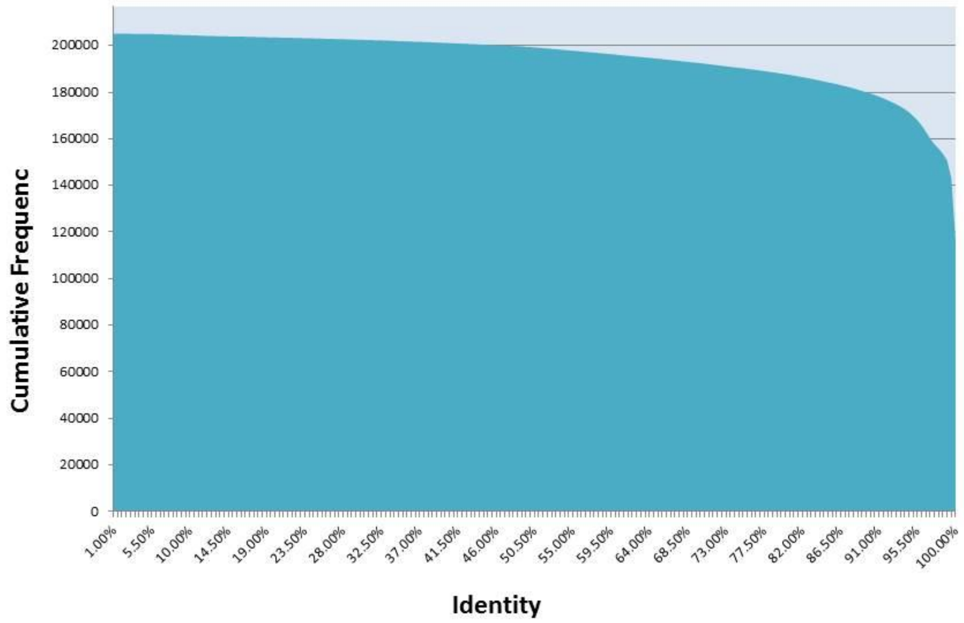

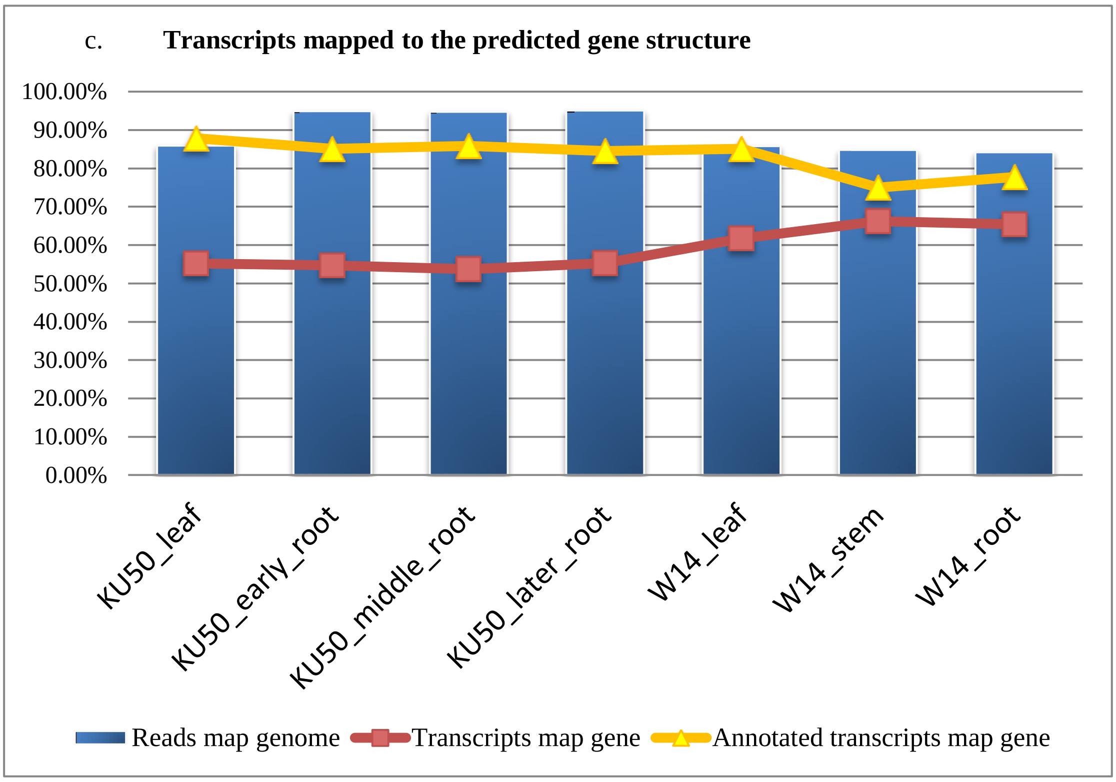

Supplementary Figure 9 Validation of gene prediction with ESTs and transcriptome

a. Over 94.9% ESTs and transcripts were mapped into W14 draft genome; b. No less than 92.8% ESTs and transcripts were mapped into to KU50 draft genome; c. Validation of predicted gene models in W14 and KU50 genome with transcriptome reads, de novo assembled transcripts and annotated transcripts alignment to ab initio predicted gene of two draft genomes. There were 55.3-66.3% transcripts and 75.0-87.9% annotated transcripts could be aligned to predicted genes in W14 and KU50 genome.

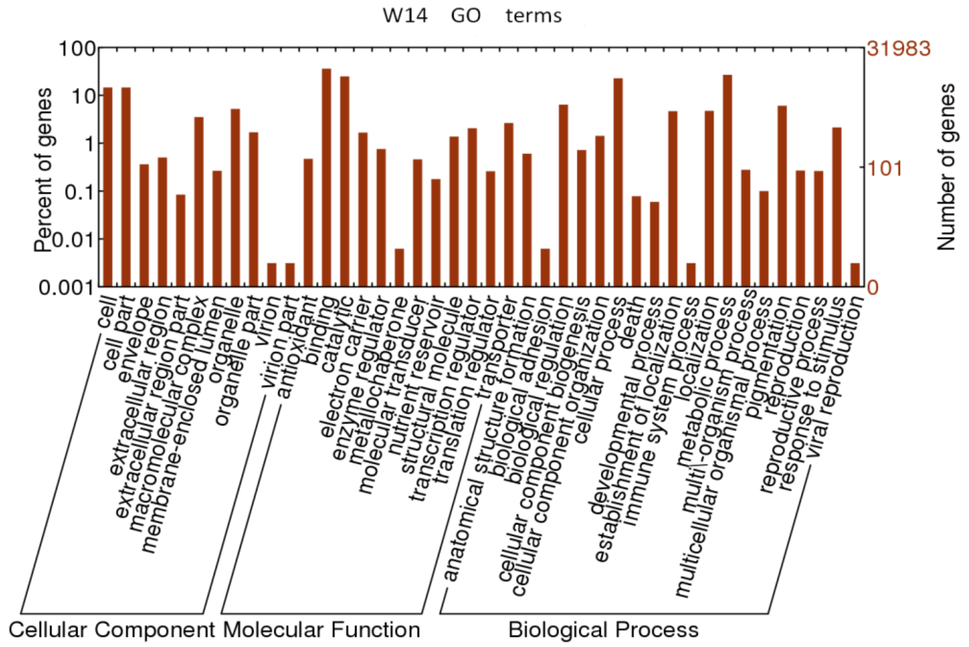

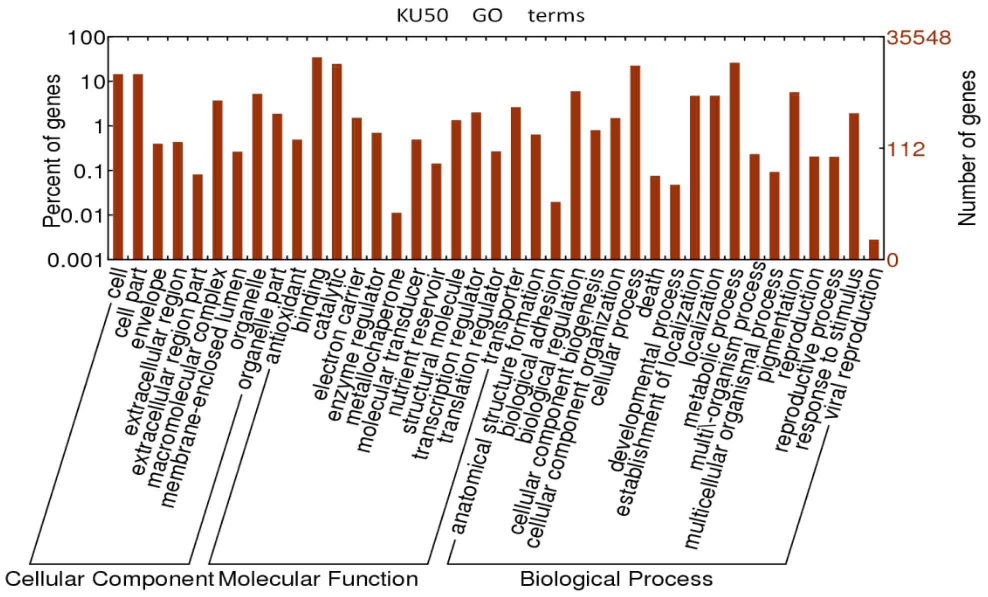

Supplementary Figure 10 Go term distribution of predicted genes of W14 and KU50

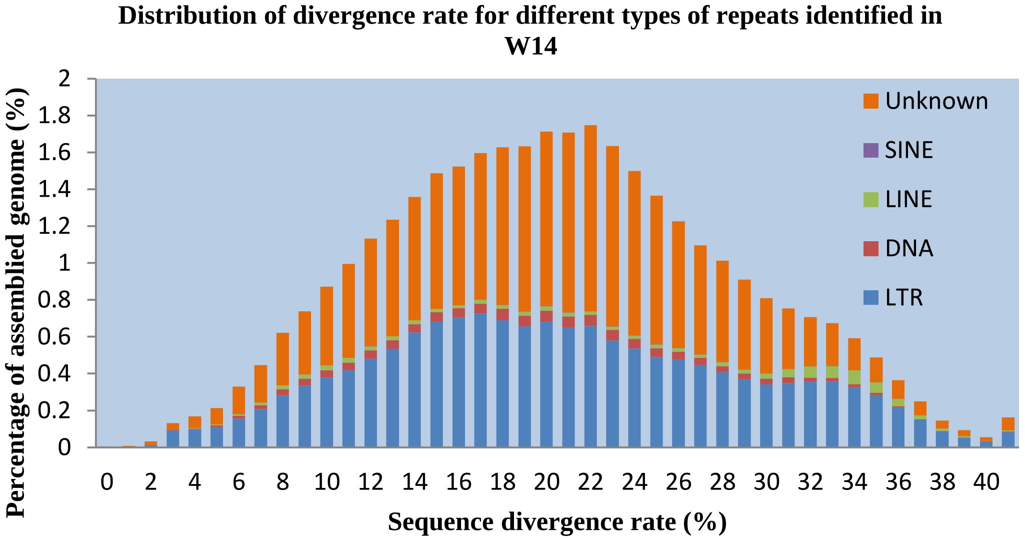

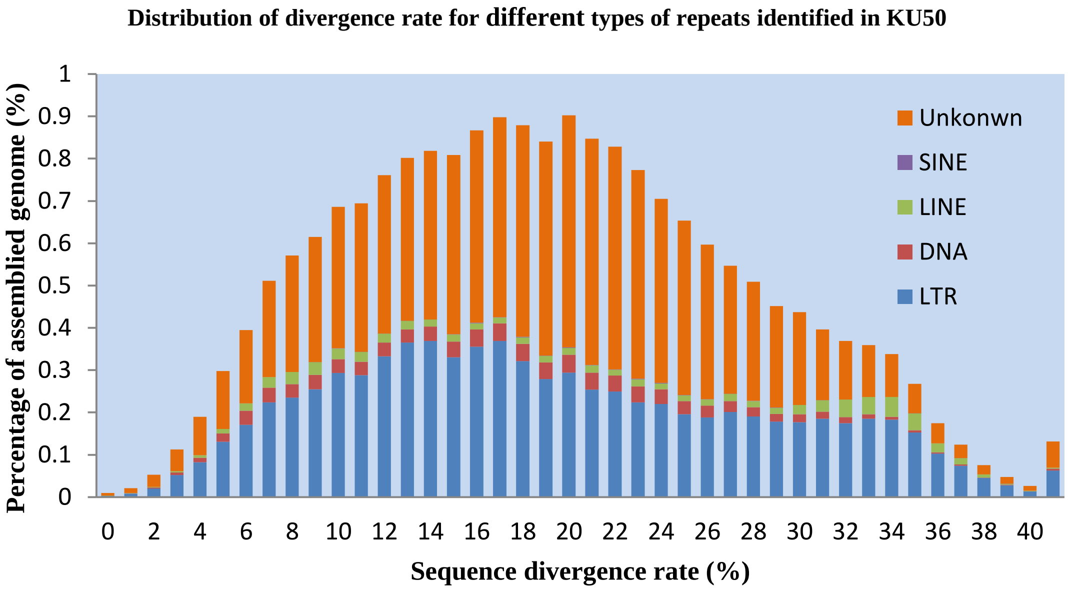

Supplementary Figure 11 Distribution of divergence rate for different types of repeats identified in the W14 and KU50 genome assemblies

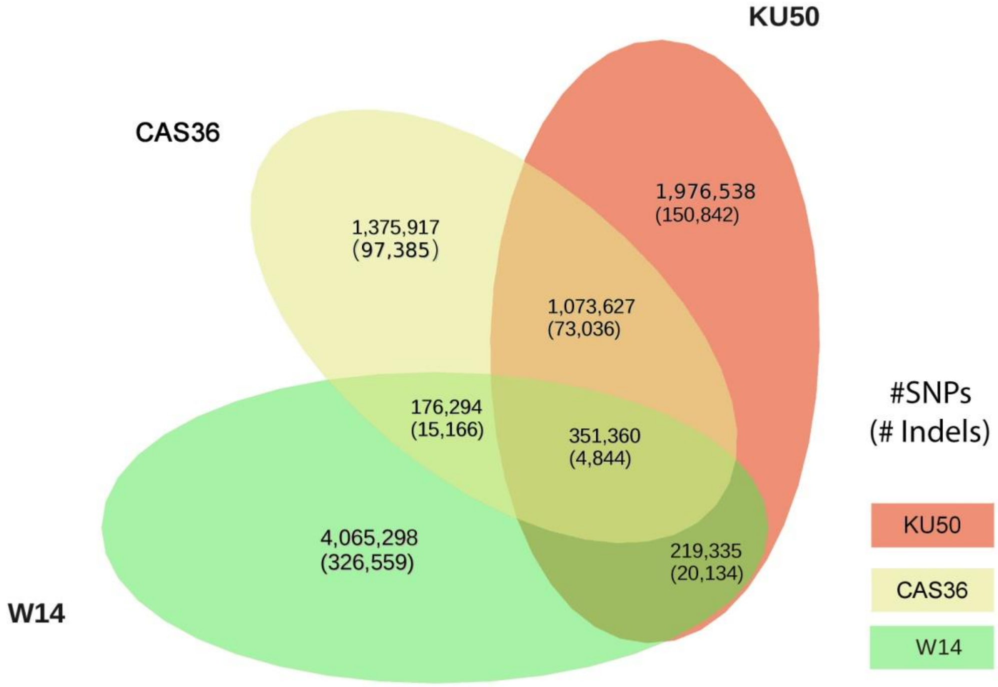

Supplementary Figure 12 Venn Diagram of numbers of SNVs and InDels between three cassava genomes, W14, KU50, and CAS36 (S1.600) to the genome of AM560

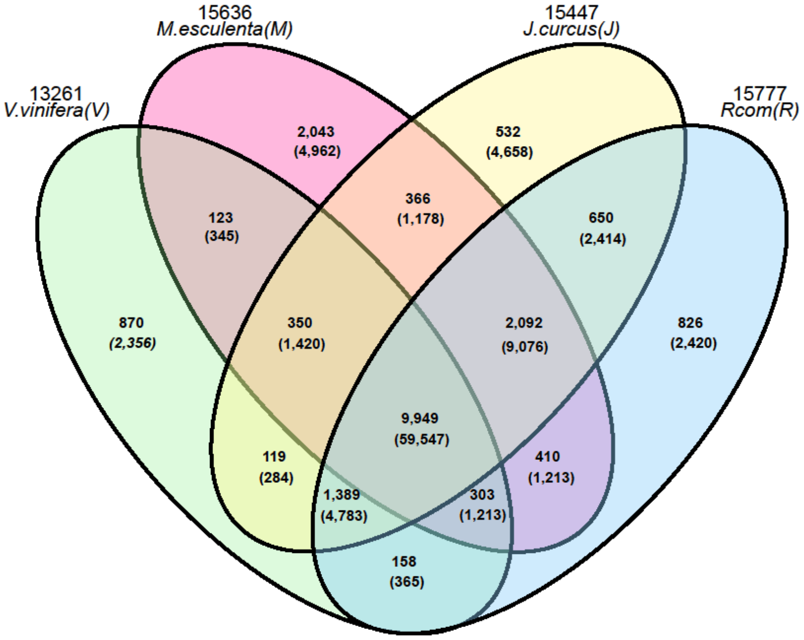

Supplementary Figure 13 Gene family analysis in Euphorbiaceae

Comparison of gene families among *M. esculenta* (15636), *J. curcus* (15447) and *R. communis* (15777) in Euphorbiaceae and *V. vinifera* (13261) revealed that there were 2,043 gene families unique in *M. esculenta*, being higher than those in *J. curcus* (532) and *R. communis* (826).

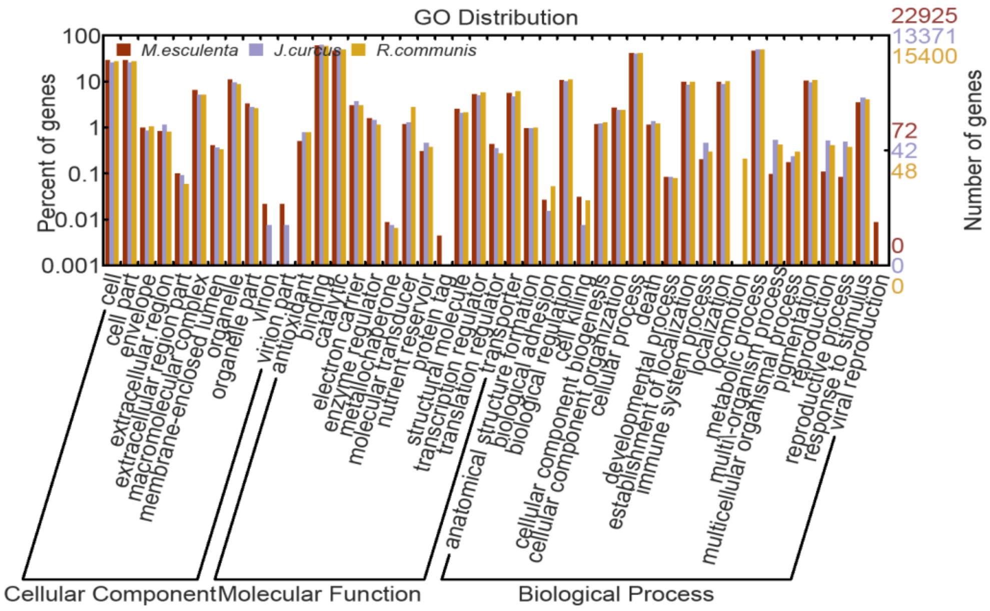

Supplementary Figure 14 Gene Ontology (GO) annotation of gene families among three Euphorbiaceae species

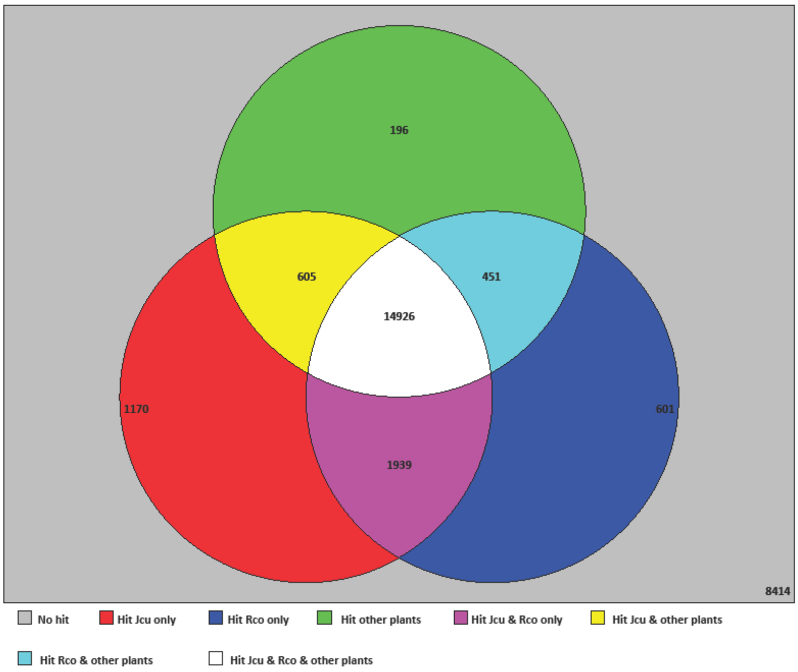

Supplementary Figure 15 Specific genes in cassava species The rectangle represents entire cassava gene models. Gray part means gene models has no hit against any other species, which are thus cassava species-specific. Three circles represent castor bean (blue), Barbadosnut (red) and 12 other model plants (green), respectively.

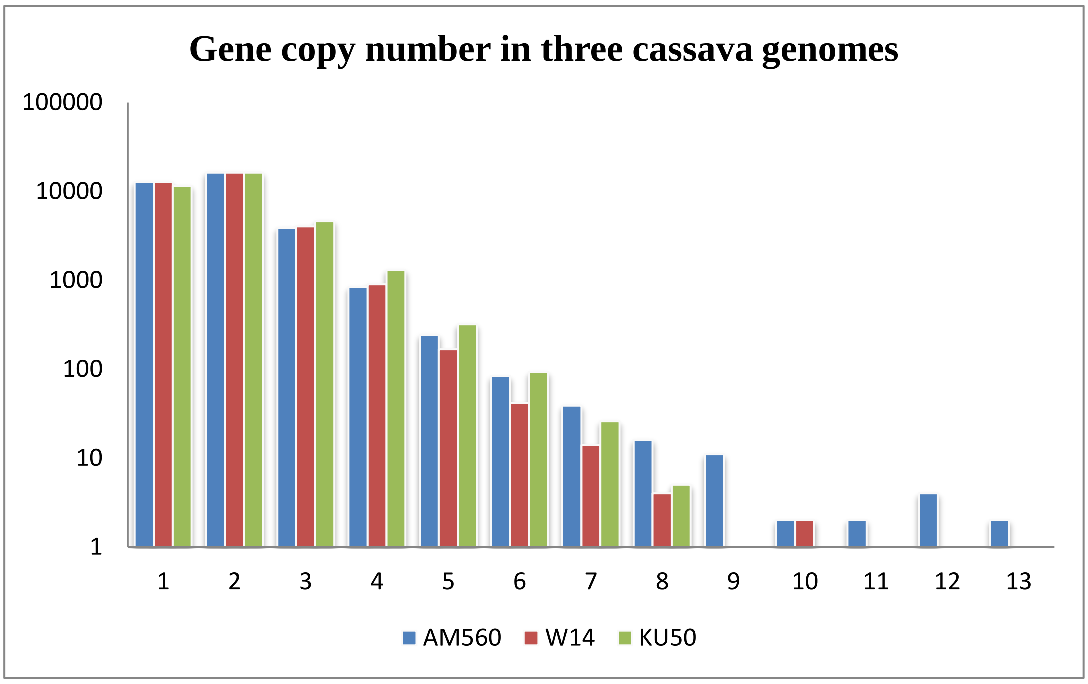

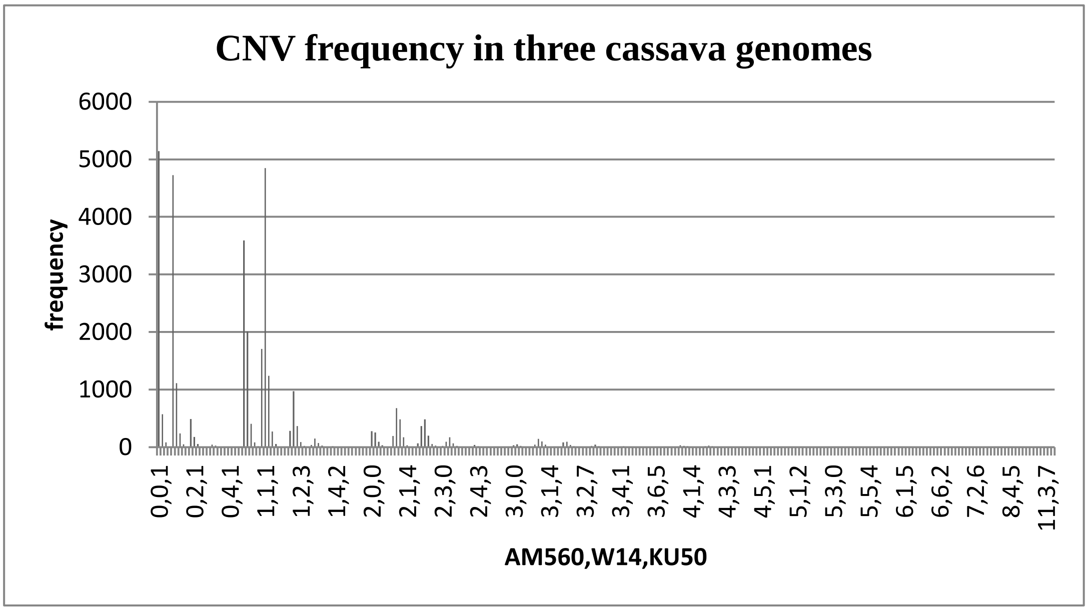

Supplementary Figure 16 Comparison of gene copy numbers and corresponding CNV frequency in the three cassava genomes

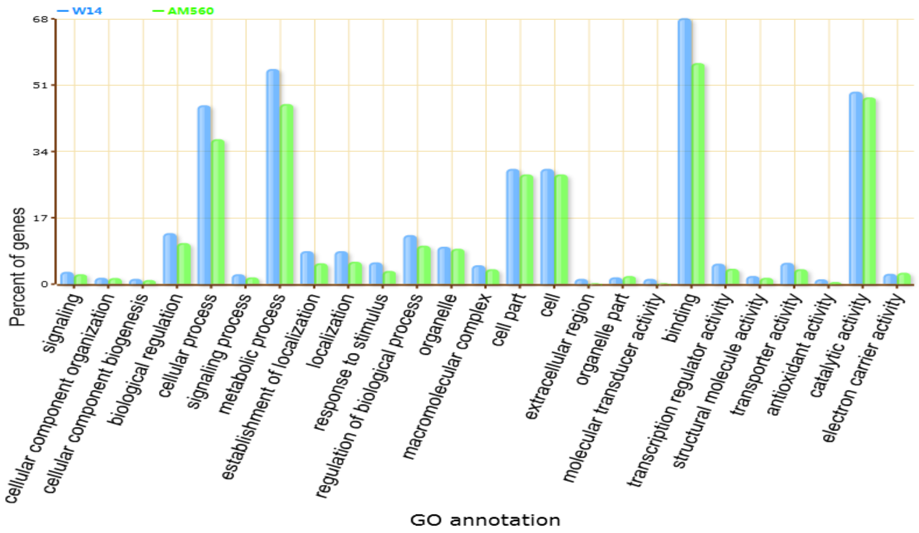

Supplementary Figure 17 GO annotation of genes with PAV between wild ancestor W14 and cultivated KU50 and AM560

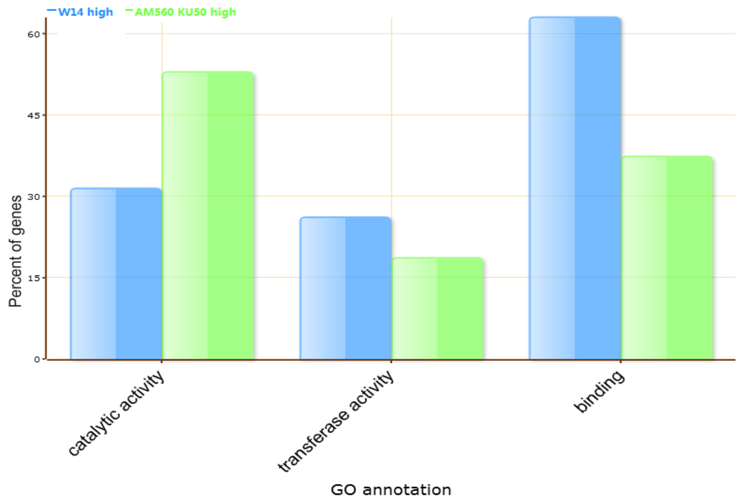

Supplementary Figure 18 GO annotated genes with significant difference in copy numbers between cultivated varieties KU50 and AM560 and wild W14

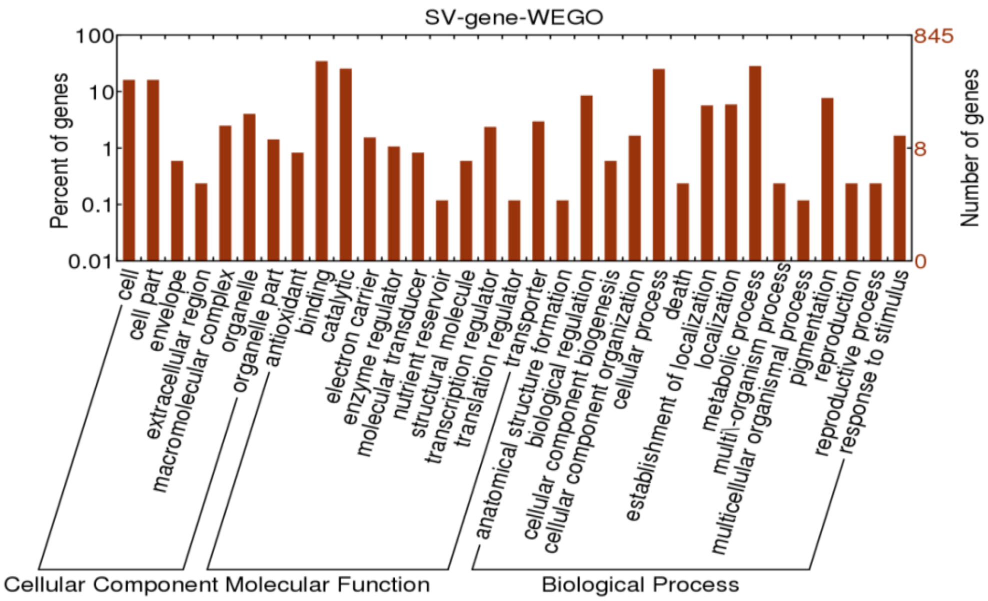

Supplementary Figure 19 GO annotation for the genes with structural variations between cultivated varieties and wild W14

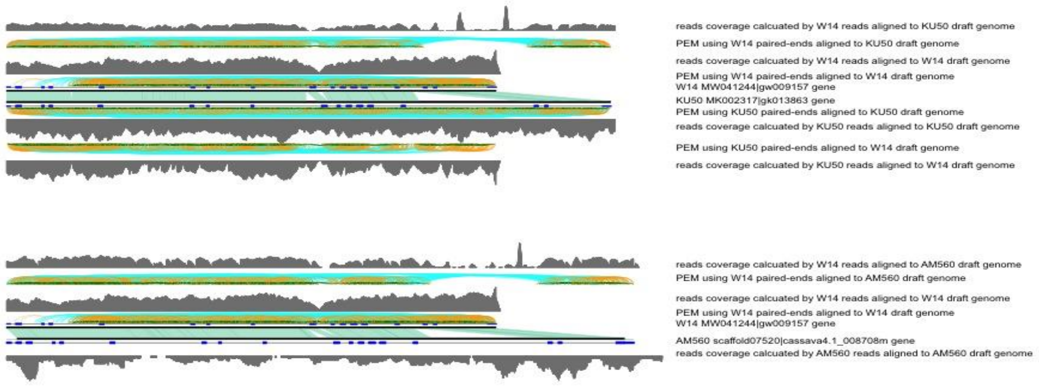

Supplementary Figure 20 SV example module: deletion-insertion between cultivars and wild subspecies

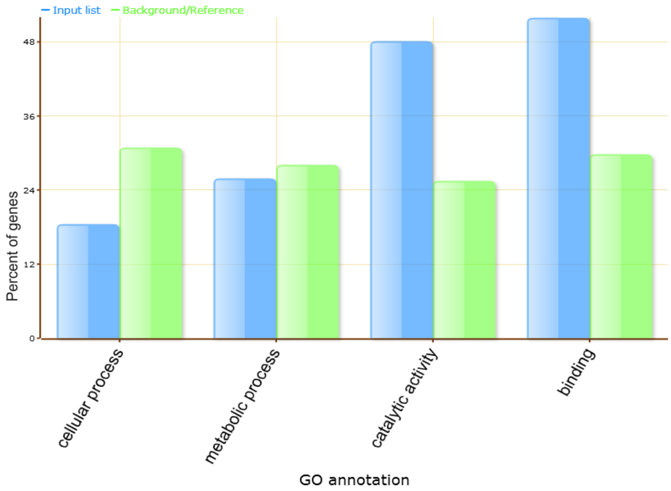

Supplementary Figure 21 GO annotation for 70 genes without SNV/InDel out of 6,567 orthologues between cultivated varieties and wild W14

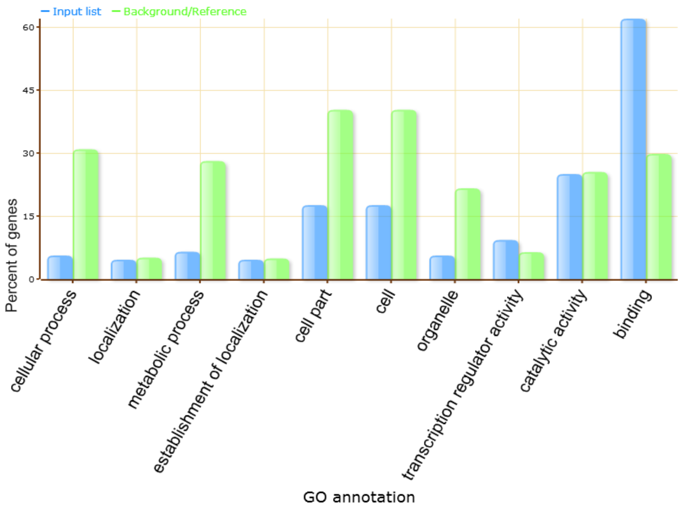

Supplementary Figure 22 GO Annotation for 277 genes with lower than 1.5% frequency of SNV/InDel between wild subspecies and cultivars

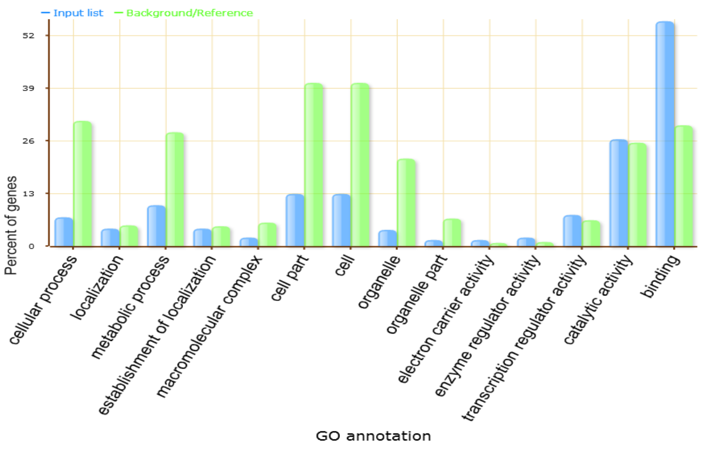

Supplementary Figure 23 GO annotation for 891 genes with SNV/InDels between wild subspecies and cultivars

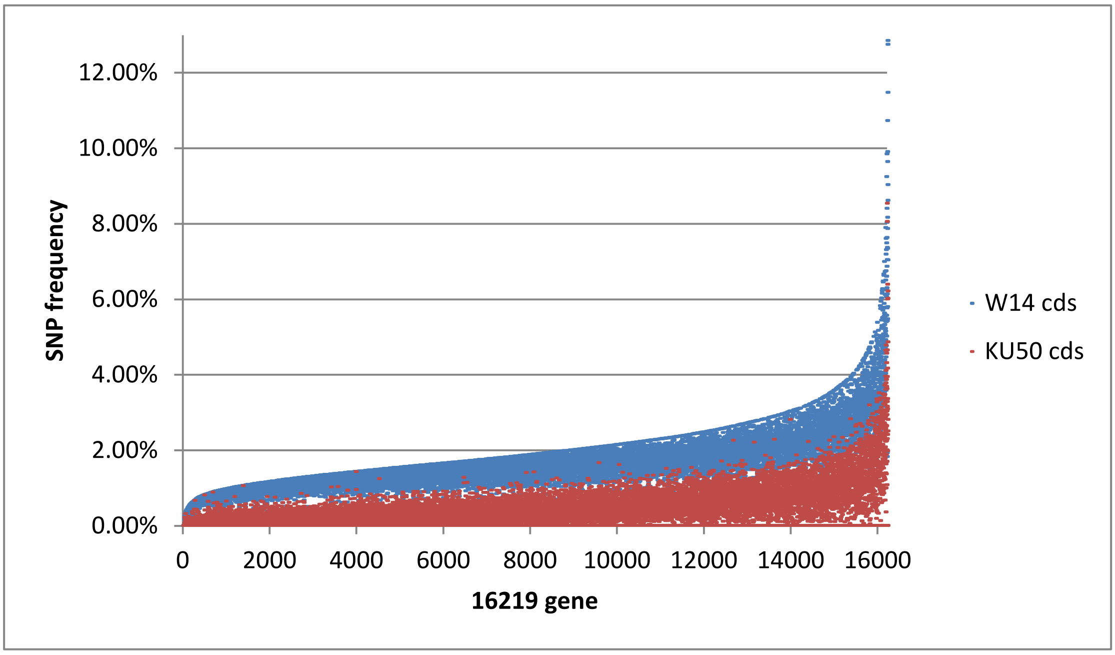

Supplementary Figure 24 A systemically different distribution of SNPs in the CDSs of the 16,219 genes between wild W14 and cultivar KU50

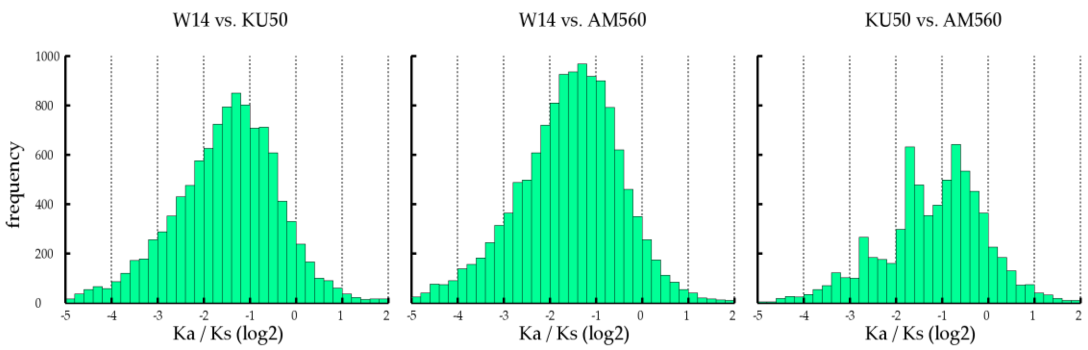

Supplementary Figure 25 Distribution of selective pressures with *Ka/Ks* (log2) between the three cassava genomes, KU50, AM560 and W14

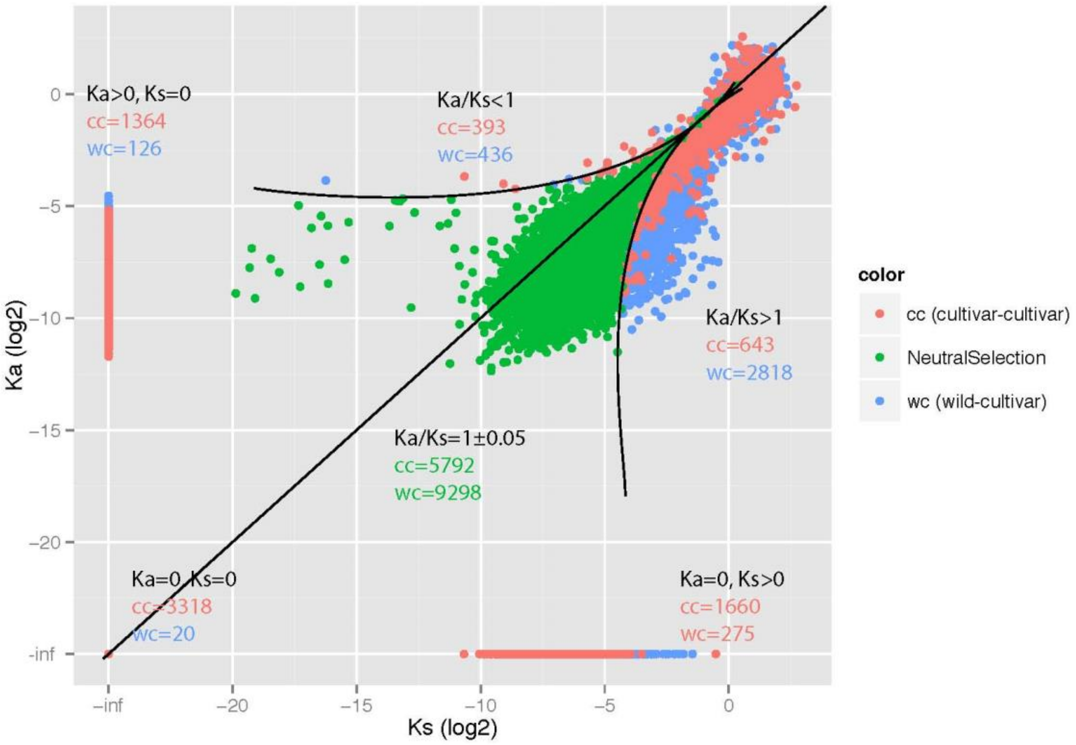

Supplementary Figure 26 Chart for synonymous substitution (*Ks*) and nonsynonymous substitution rate (*Ka*) and selection pressure (*Ka/Ks*) between wild W14 and cultivar (cw) and between cultivars (cc)

Ka/Ks=1 means genes with neutral selection, *Ka/Ks*>1 means positive selection and *Ka/Ks*<1 means negative selection. Genes with *Ka=Ks=0, Ka=0, Ks*>0 and *Ka*>0, or *Ks*=0 have very low selection pressure. It was shown that 2,818 genes were restrictively positively selected (*Ka/Ks*>1), 436 genes were negatively selected (*Ka/Ks*<1) and 9,298 genes were selected in a neutral manner from wild ancestor to cultivar. But, between cultivars, only 1,036 genes were selected strictly (*Ka/Ks*>1 and *Ka/Ks*<1) and 6,342 genes have very low selection pressure (*Ka*=*Ks*=0, *Ka*=0, *Ks*>0 and *Ka*>0, *Ks*=0). By comparison among them, we found that 1,133 genes have been selected severely during natural and domesticated evolution and caused clearly selection sweeping.

A

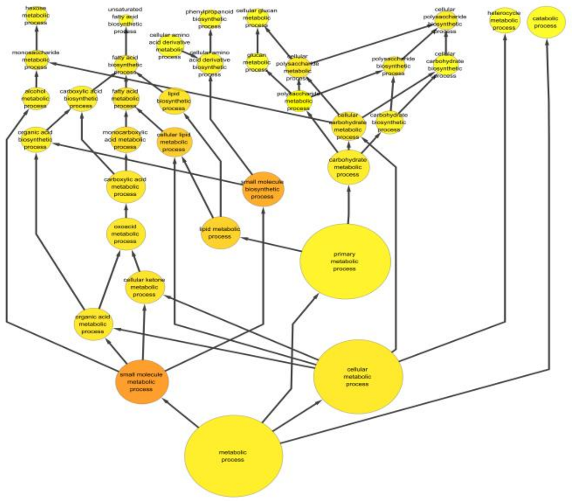
hexose metabolic process
unsaturated fatty acid biosynthetic process
phenylpropanoid biosynthetic process
cellular glucan metabolic process
cellular polysaccharide biosynthetic process
heterocycle metabolic process
catabolic process
cellular amino acid derivative metabolic process
monosaccharide metabolic process
fatty acid biosynthetic process
cellular amino acid derivative biosynthetic process
glucan metabolic process
cellular polysaccharide metabolic process
polysaccharide biosynthetic process
cellular carbohydrate biosynthetic process
alcohol metabolic process
carboxylic acid biosynthetic process
fatty acid metabolic process
lipid biosynthetic process
polysaccharide metabolic process
cellular carbohydrate metabolic process
carbohydrate biosynthetic process
organic acid biosynthetic process
monocarboxylic acid metabolic process
cellular lipid metabolic process
carboxylic acid metabolic process
small molecule biosynthetic process
carbohydrate metabolic process
oxoacid metabolic process
lipid metabolic process
primary metabolic process
cellular ketone metabolic process
organic acid metabolic process
small molecule metabolic process
cellular metabolic process
metabolic process

B

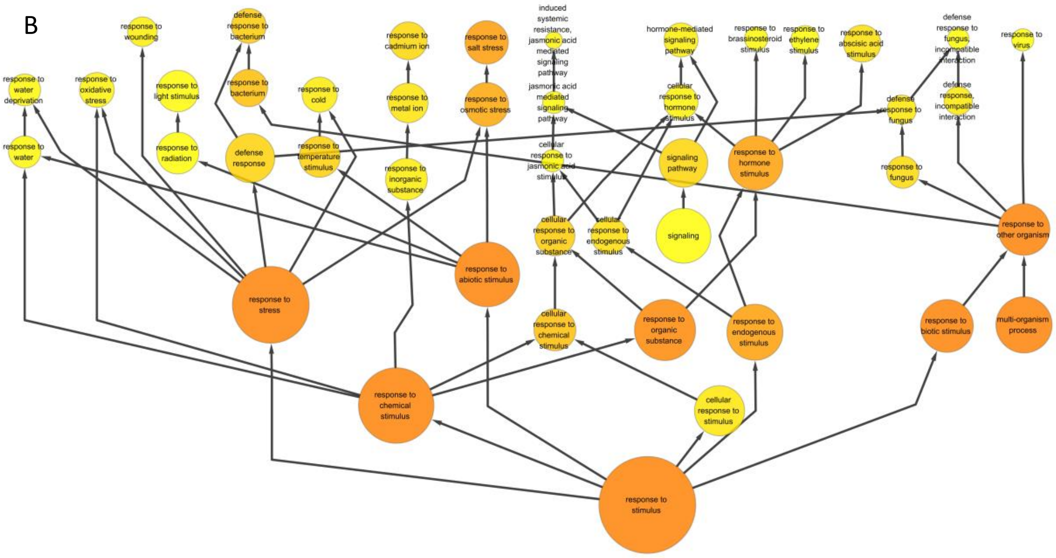
response to wounding
defense response to bacterium
response to cadmium ion
response to salt stress
induced systemic resistance, jasmonic acid mediated signaling pathway
hormone-mediated signaling pathway
response to brassinosteroid stimulus
response to ethylene stimulus
response to abscisic acid stimulus
defense response to fungus, incompatible interaction
response to virus
response to water deprivation
response to oxidative stress
response to light stimulus
response to bacterium
response to cold
response to metal ion
response to osmotic stress
jasmonic acid mediated signaling pathway
cellular response to hormone stimulus
defense response to fungus
defense response, incompatible interaction
response to water
response to radiation
defense response
response to temperature stimulus
response to inorganic substance
cellular response to jasmonic acid stimulus
signaling pathway
response to hormone stimulus
response to fungus
cellular response to organic substance
cellular response to endogenous stimulus
signaling
response to other organism
response to stress
response to abiotic stimulus
cellular response to chemical stimulus
response to organic substance
response to endogenous stimulus
response to biotic stimulus
multi-organism process
response to chemical stimulus
cellular response to stimulus
response to stimulus

C

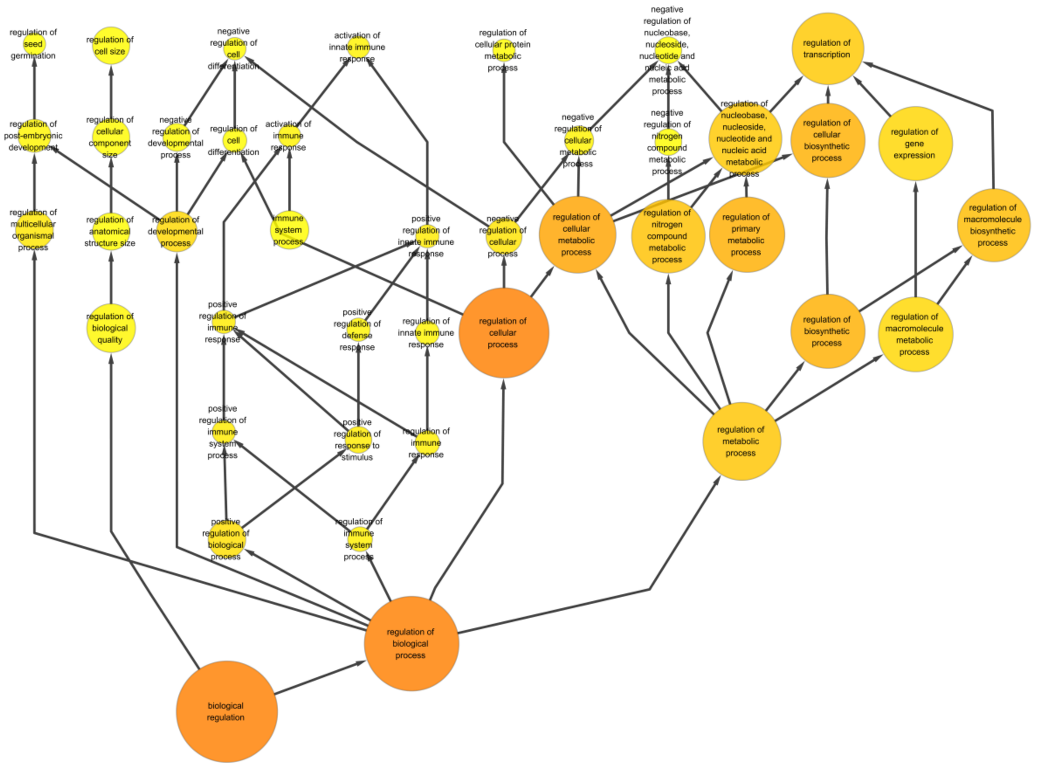
regulation of seed germination
regulation of cell size
negative regulation of cell differentiation
activation of innate immune response
regulation of cellular protein metabolic process
negative regulation of nucleobase, nucleoside, nucleotide and nucleic acid metabolic process
regulation of transcription
regulation of post-embryonic development
regulation of cellular component size
negative regulation of developmental process
regulation of cell differentiation
activation of immune response
negative regulation of cellular metabolic process
negative regulation of nitrogen compound metabolic process
regulation of nucleobase, nucleoside, nucleotide and nucleic acid metabolic process
regulation of cellular biosynthetic process
regulation of gene expression
regulation of multicellular organismal process
regulation of anatomical structure size
regulation of developmental process
immune system process
positive regulation of innate immune response
negative regulation of cellular process
regulation of cellular metabolic process
regulation of nitrogen compound metabolic process
regulation of primary metabolic process
regulation of macromolecule biosynthetic process
regulation of biological quality
positive regulation of immune response
positive regulation of defense response
regulation of innate immune response
regulation of cellular process
regulation of biosynthetic process
regulation of macromolecule metabolic process
positive regulation of immune system process
positive regulation of response to stimulus
regulation of immune response
regulation of metabolic process
positive regulation of biological process
regulation of immune system process
regulation of biological process
biological regulation

D

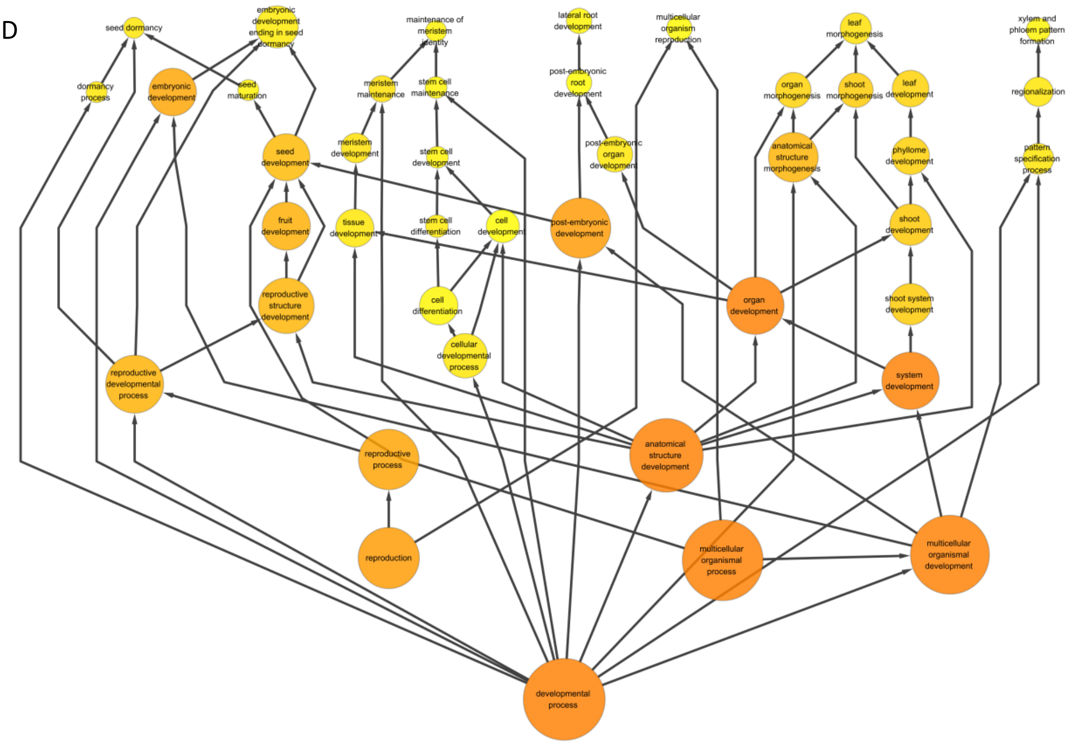
seed dormancy
embryonic development ending in seed dormancy
maintenance of meristem identity
lateral root development
multicellular organism reproduction
leaf morphogenesis
xylem and phloem pattern formation
dormancy process
embryonic development
seed maturation
meristem maintenance
stem cell maintenance
post-embryonic root development
organ morphogenesis
shoot morphogenesis
leaf development
regionalization
seed development
meristem development
stem cell development
post-embryonic organ development
anatomical structure morphogenesis
phyllome development
pattern specification process
fruit development
tissue development
stem cell differentiation
cell development
post-embryonic development
shoot development
reproductive structure development
cell differentiation
organ development
shoot system development
cellular developmental process
reproductive developmental process
system development
reproductive process
anatomical structure development
reproduction
multicellular organismal process
multicellular organismal development
developmental process

E

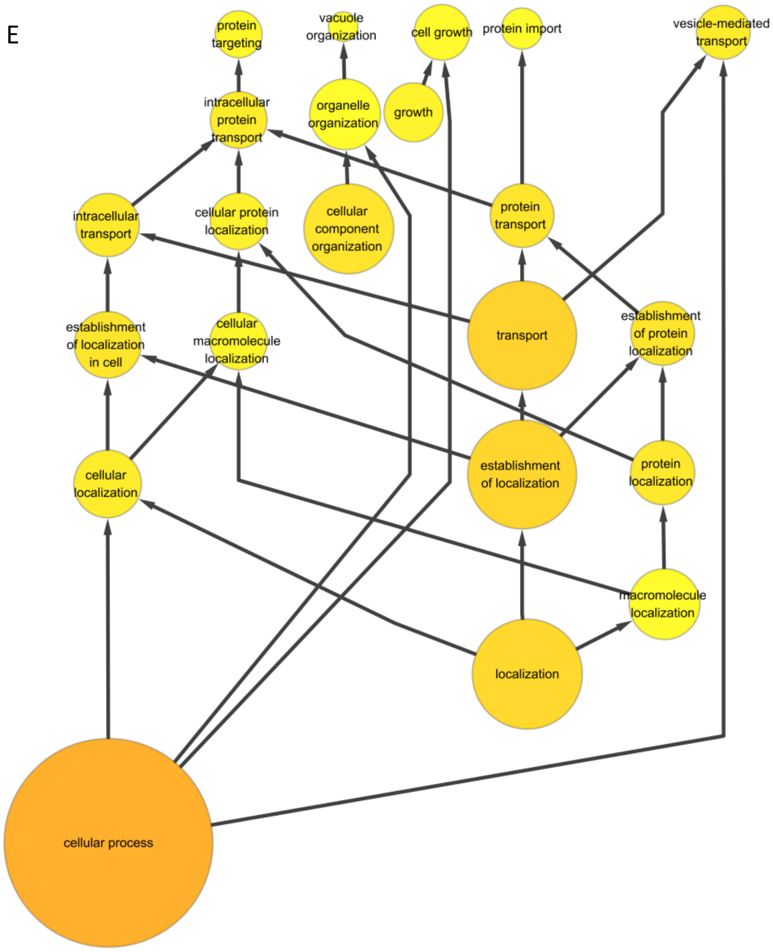

protein targeting
vacuole organization
cell growth
protein import
vesicle-mediated transport
intracellular protein transport
organelle organization
growth
intracellular transport
cellular protein localization
cellular component organization
protein transport
establishment of localization in cell
cellular macromolecule localization
transport
establishment of protein localization
cellular localization
establishment of localization
protein localization
macromolecule localization
localization
cellular process

F

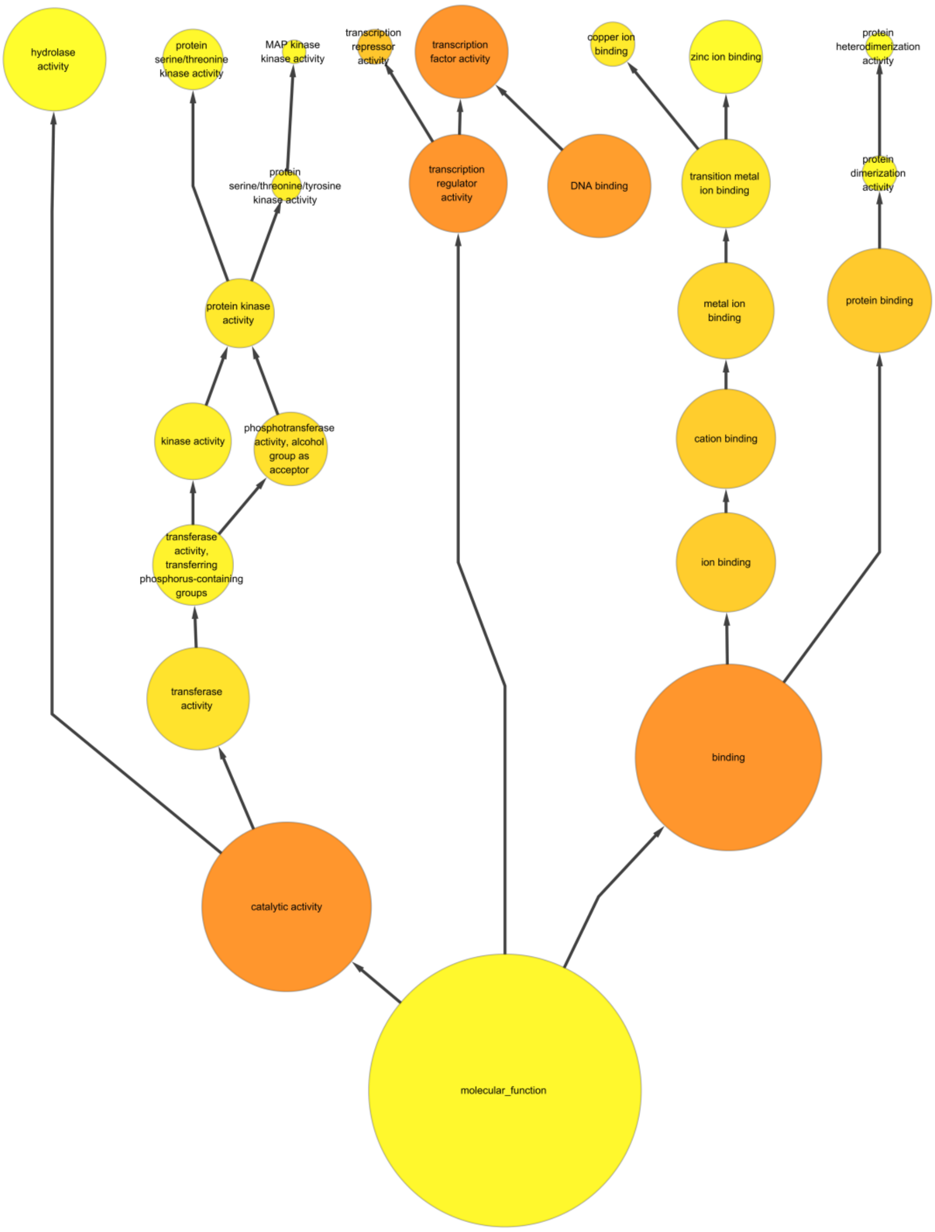
hydrolase activity
protein serine/threonine kinase activity
MAP kinase kinase activity
transcription repressor activity
transcription factor activity
copper ion binding
zinc ion binding
protein heterodimerization activity
protein serine/threonine/tyrosine kinase activity
transcription regulator activity
DNA binding
transition metal ion binding
protein dimerization activity
protein kinase activity
metal ion binding
protein binding
kinase activity
phosphotransferase activity, alcohol group as acceptor
cation binding
transferase activity, transferring phosphorus-containing groups
ion binding
transferase activity
binding
catalytic activity
molecular_function

G

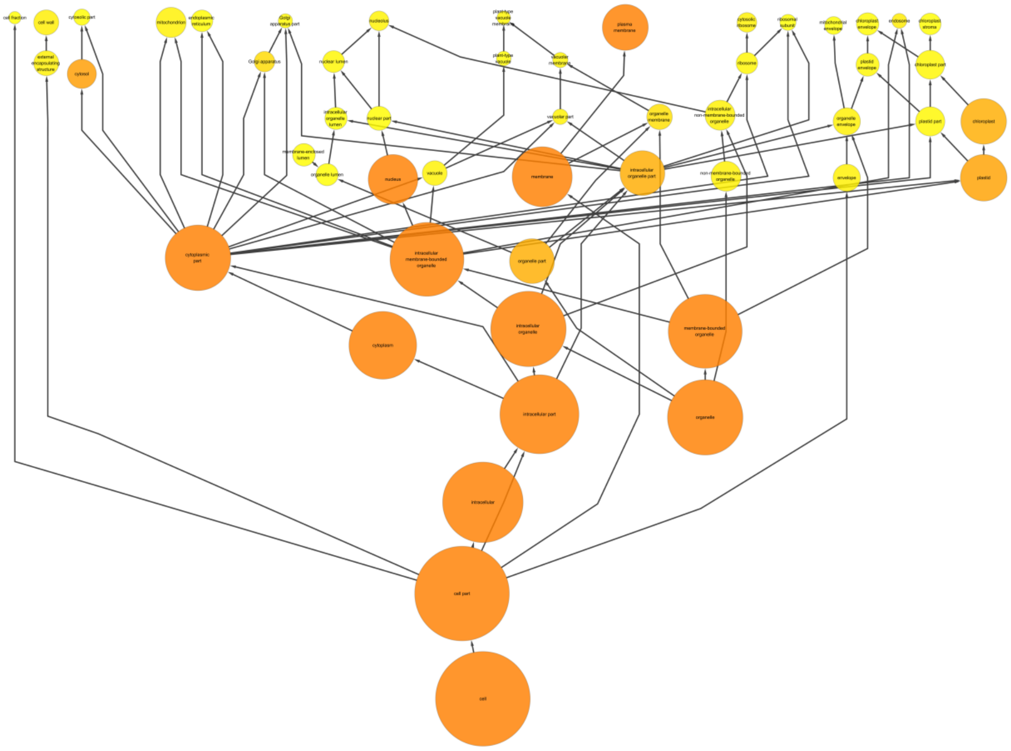

Supplementary Figure 27 BINGO enrichment analysis for genes that have been positively or negatively selected between W14 and cultivated cassava

(A) Genes enriched in the functional subcategory of metabolic processes. (B) Genes enriched in the functional subcategory of response to stimulus. (C) Genes enriched in the functional subcategory of biological regulation. (D) Genes enriched in the functional subcategory of developmental process. (E) Genes enriched in the functional subcategory of cellular process. (F) Genes enriched in the functional category of molecular function. (G) Genes enriched in the functional subcategory of cell part.

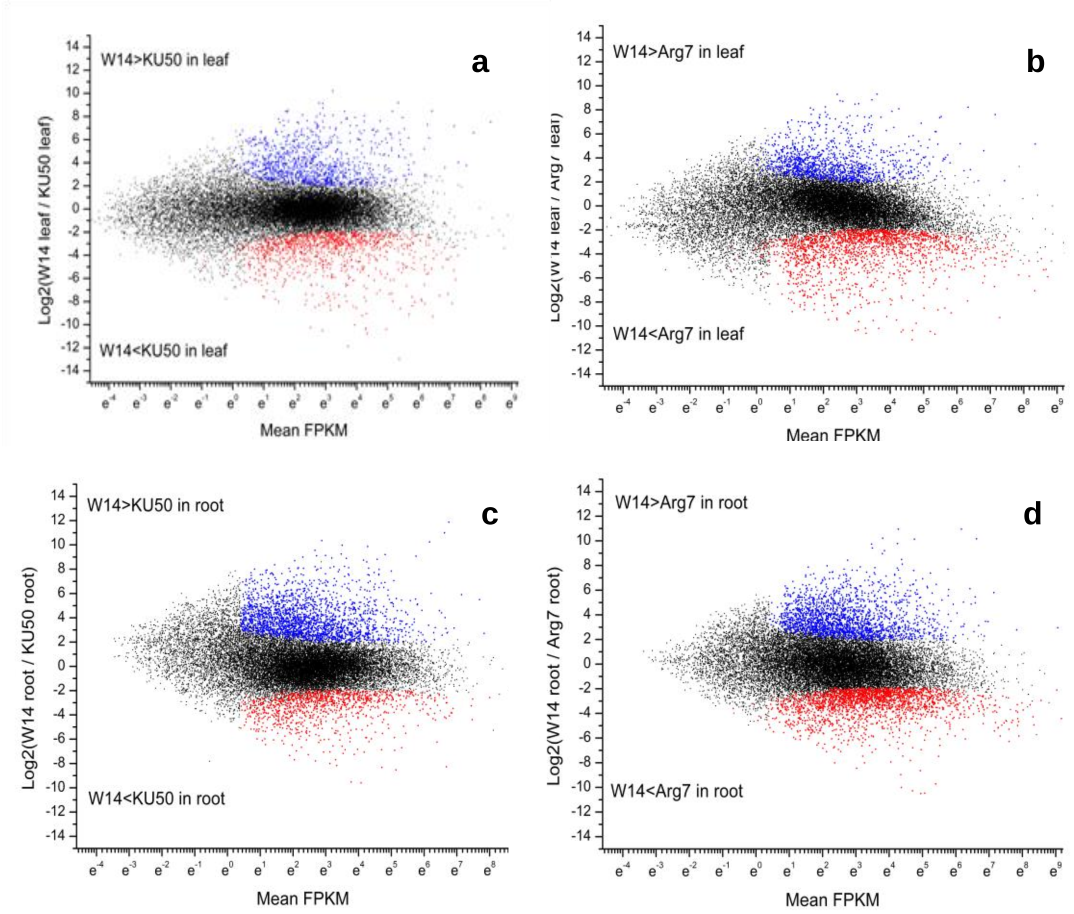

Supplementary Figure 28 Comparing transcriptomes in leaf and storage root between wild W14 and cultivated KU50 and Arg7

The blue and red dots mean the genes significantly different expressed than control with Log2FPKM reached to o.o5 P-value.

A

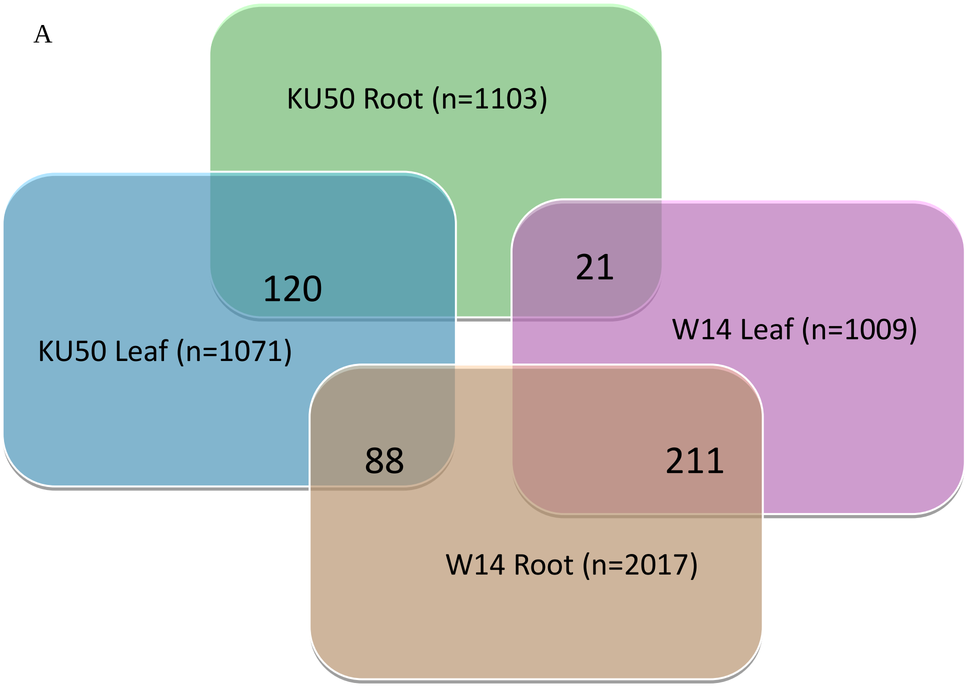

B

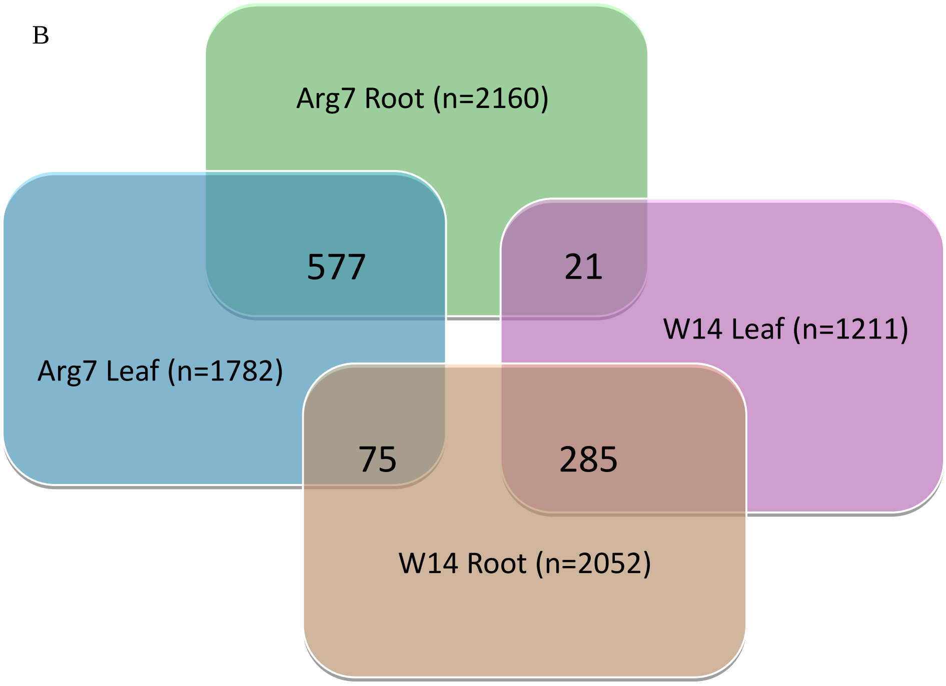

Supplementary Figure 29 Numbers of significantly expressed genes in leaf and storage root between wild W14 and cultivated KU50 and Arg7

A significant difference level of *P*-value ≤ 0.05 is referenced to a fold change of >3.0. (A) KU50 vs. W14. (B) Arg7 vs. W14.

A

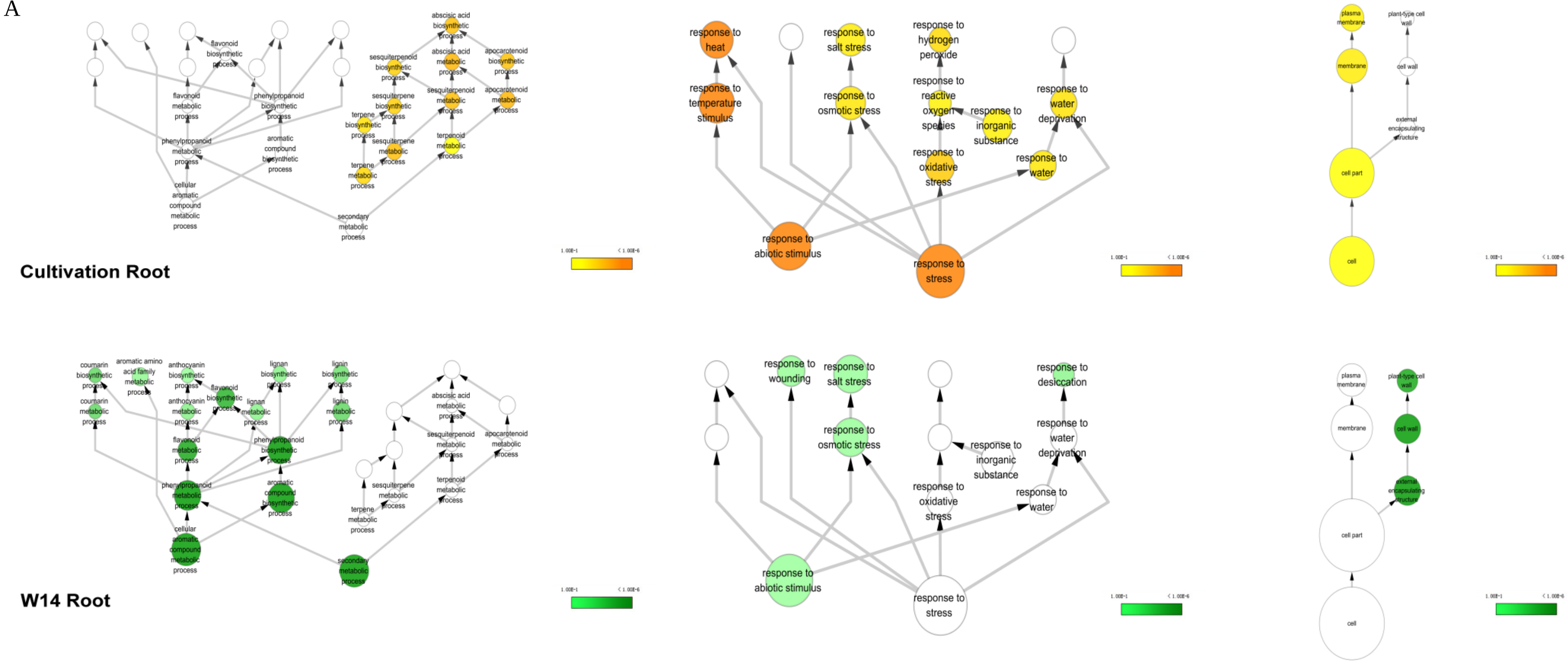

Cultivation Root
W14 Root
flavonoid biosynthetic process
flavonoid metabolic process
phenylpropanoid biosynthetic process
phenylpropanoid metabolic process
aromatic compound biosynthetic process
cellular aromatic compound metabolic process
secondary metabolic process
abscisic acid biosynthetic process
abscisic acid metabolic process
apocarotenoid biosynthetic process
apocarotenoid metabolic process
sesquiterpenoid biosynthetic process
sesquiterpene biosynthetic process
sesquiterpenoid metabolic process
sesquiterpene metabolic process
terpene biosynthetic process
terpene metabolic process
terpenoid metabolic process
coumarin biosynthetic process
coumarin metabolic process
aromatic amino acid family metabolic process
anthocyanin biosynthetic process
anthocyanin metabolic process
lignan biosynthetic process
lignan metabolic process
lignin biosynthetic process
lignin metabolic process
response to heat
response to temperature stimulus
response to salt stress
response to osmotic stress
response to hydrogen peroxide
response to reactive oxygen species
response to inorganic substance
response to oxidative stress
response to water deprivation
response to water
response to abiotic stimulus
response to stress
response to wounding
response to desiccation
plasma membrane
plant-type cell wall
membrane
cell wall
external encapsulating structure
cell part
cell

B

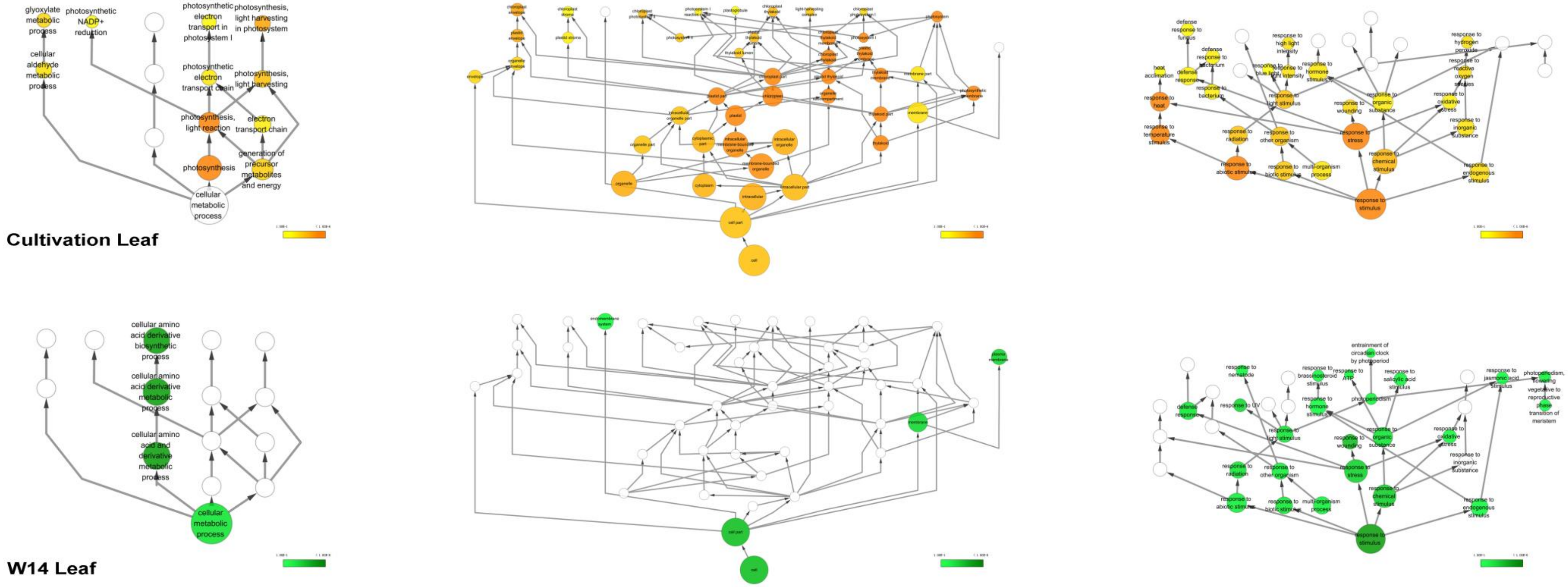
glyoxylate metabolic process
photosynthetic NADP+ reduction
photosynthetic electron transport in photosystem I
photosynthesis, light harvesting in photosystem
cellular aldehyde metabolic process
photosynthetic electron transport chain
photosynthesis, light harvesting
photosynthesis, light reaction
electron transport chain
photosynthesis
generation of precursor metabolites and energy
cellular metabolic process
Cultivation Leaf
cellular amino acid derivative biosynthetic process
cellular amino acid derivative metabolic process
cellular amino acid and derivative metabolic process
cellular metabolic process
W14 Leaf

C

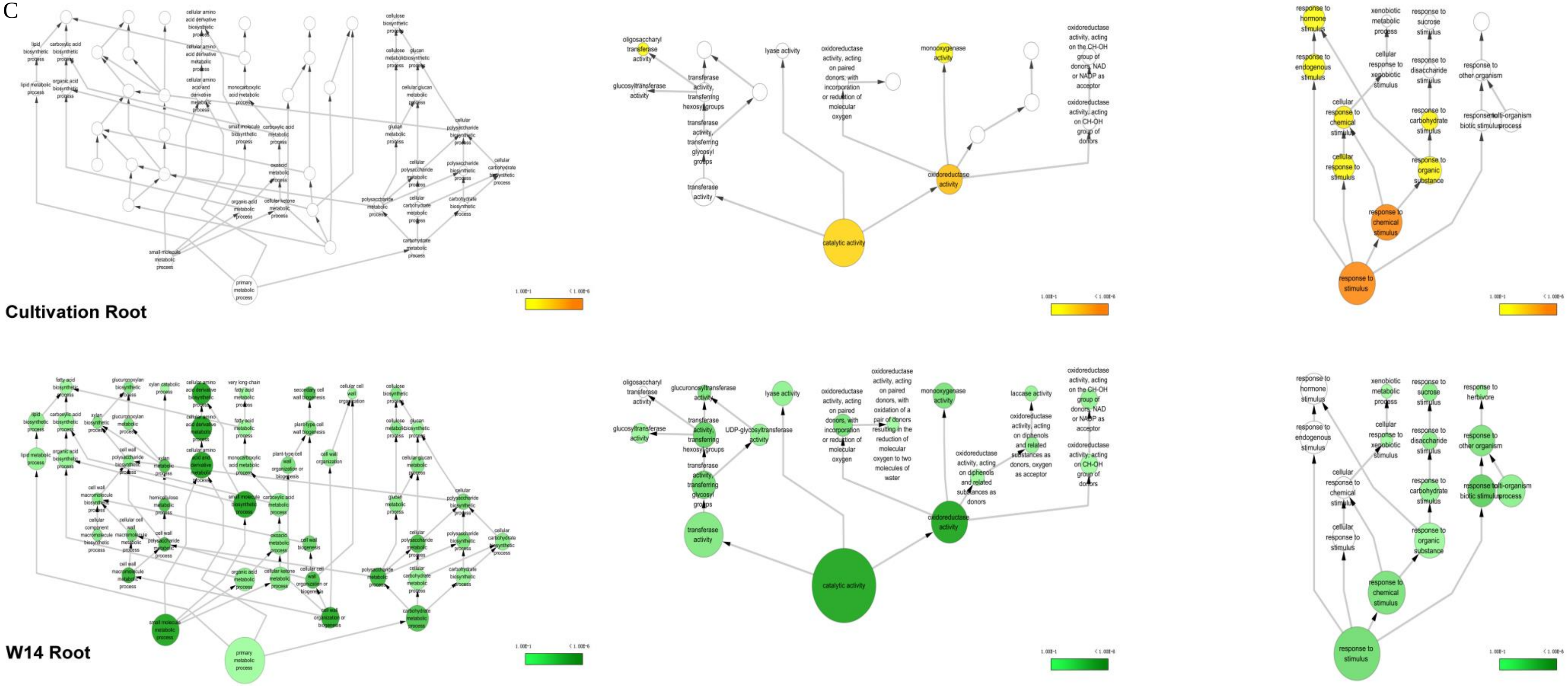

D

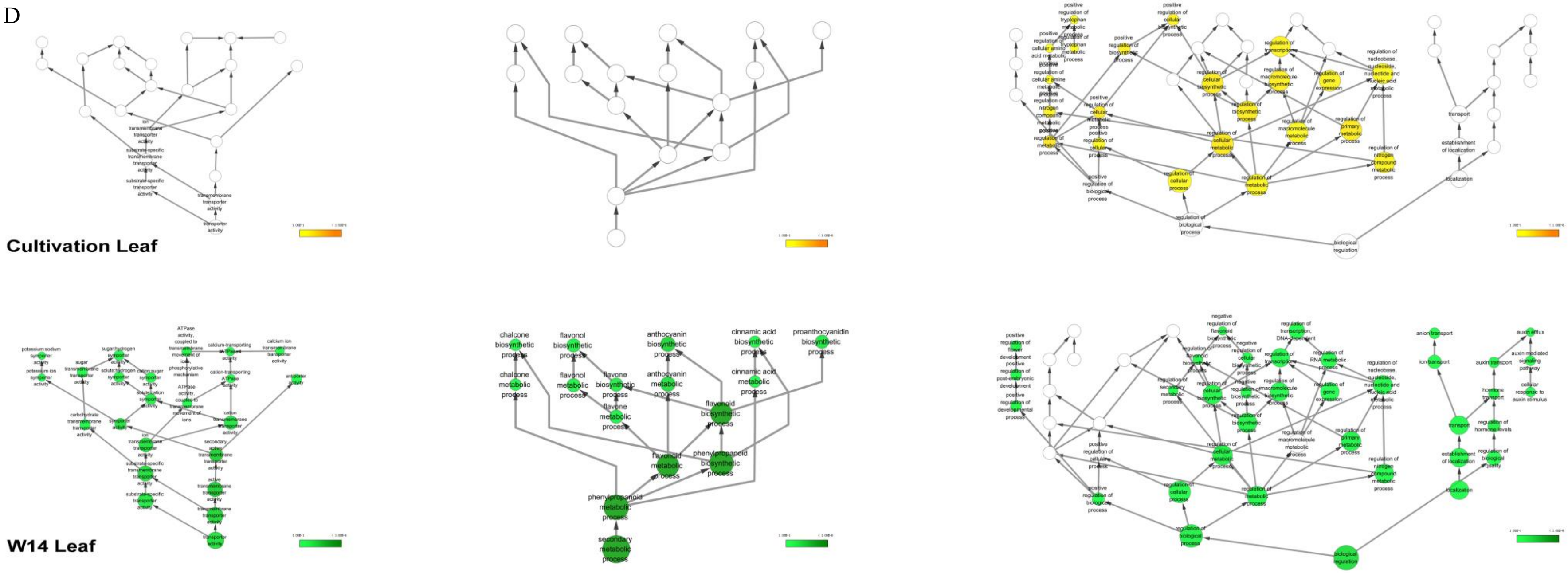

Supplementary Figure 30 Comparison of wild ancestor and cultivated cassava transcriptomes: GO enrichment analysis

(A) KU50-Arg7>W14 in storage root. (B) KU50-Arg7>W14 in functional leaf. (C) W14>KU50-Arg7 in storage root. (D) W14>KU50-Arg7 in functional leaf.

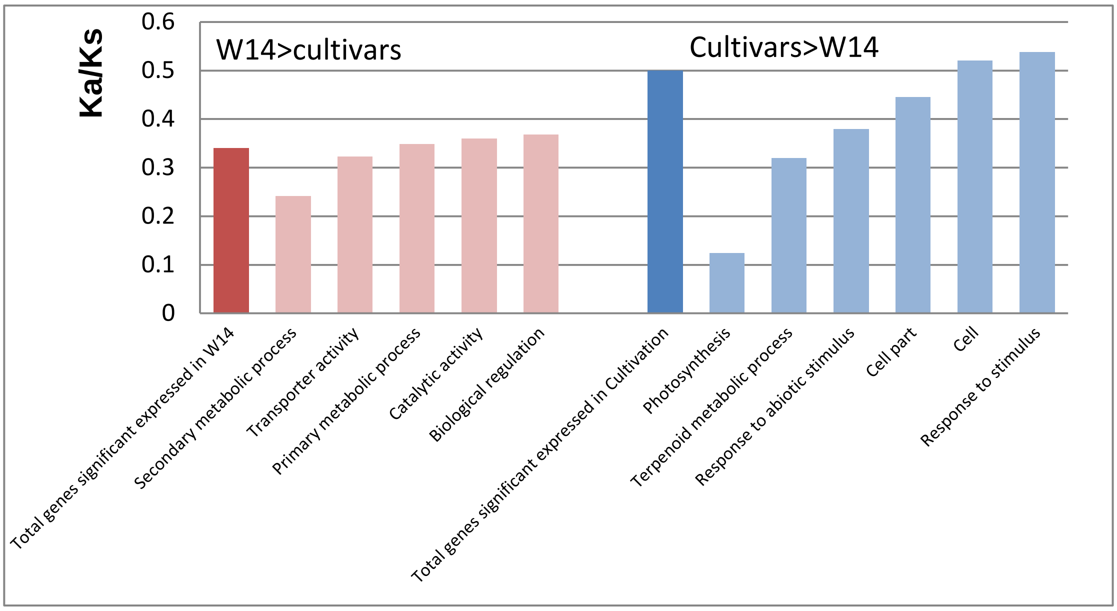

Supplementary Figure 31 Selection pressure (*Ka/Ks*) driving transcriptome evolution from wild to cultivated cassava

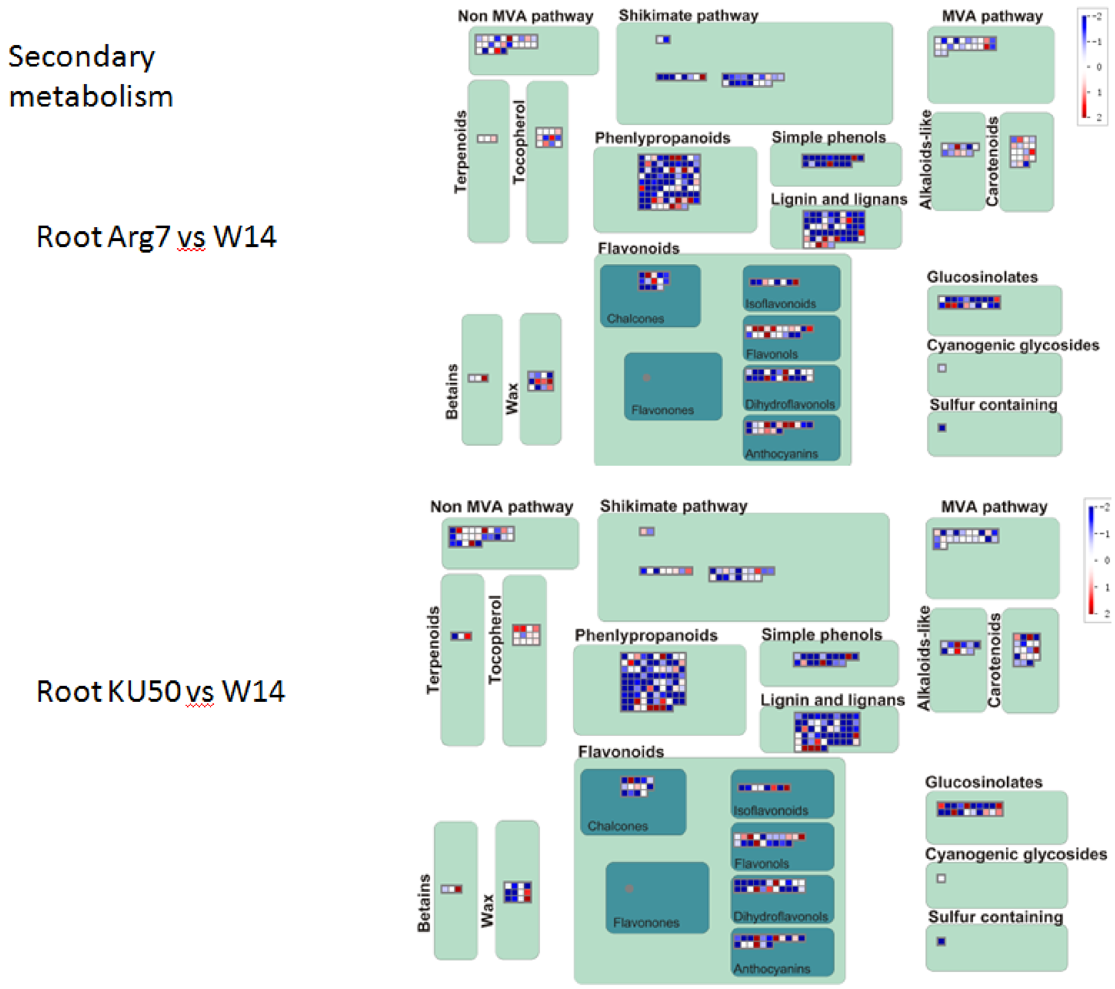

Supplementary Figure 32 Lower expression patterns of genes for secondary metabolism in storage root of Arg7 and KU50 than W14

Mapman images indicate the expression differences of genes that are related to the secondary metabolism between Arg7 and W14 and between KU50 and W14. The log2 ratios of Arg7 vs. W14 and KU50 vs. W14 were used to draw the Mapman images. The color of blue means that the gene has a higher expression level in W14 and that of red means that the gene has a higher expression level in cultivar (Arg7 or KU50).

Cell Wall precursor

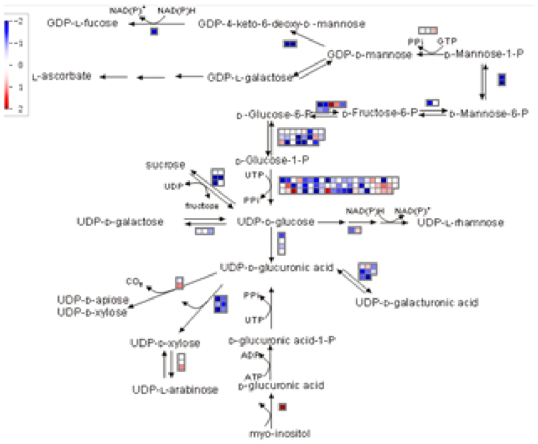

Root Arg7 vs. W14

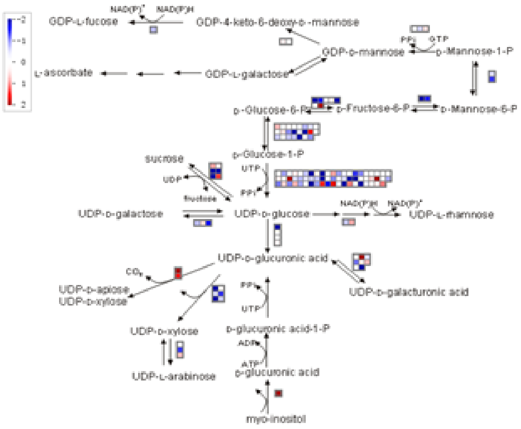

Root KU50 vs. W14

Supplementary Figure 33 lower expression of genes for cell wall synthesis in KU50, Arg7 than W14.

Mapman images indicate that the genes are related to the cell wall precursors between Arg7 and W14, and between KU50 and W14. The log2 ratios of Arg7 vs. W14 and KU50 vs. W14 were used to draw the Mapman images. The color of blue means that the gene has a higher expression level in wild W14 and that of red means that the gene has a higher expression level in cultivar (Arg7 or KU50).

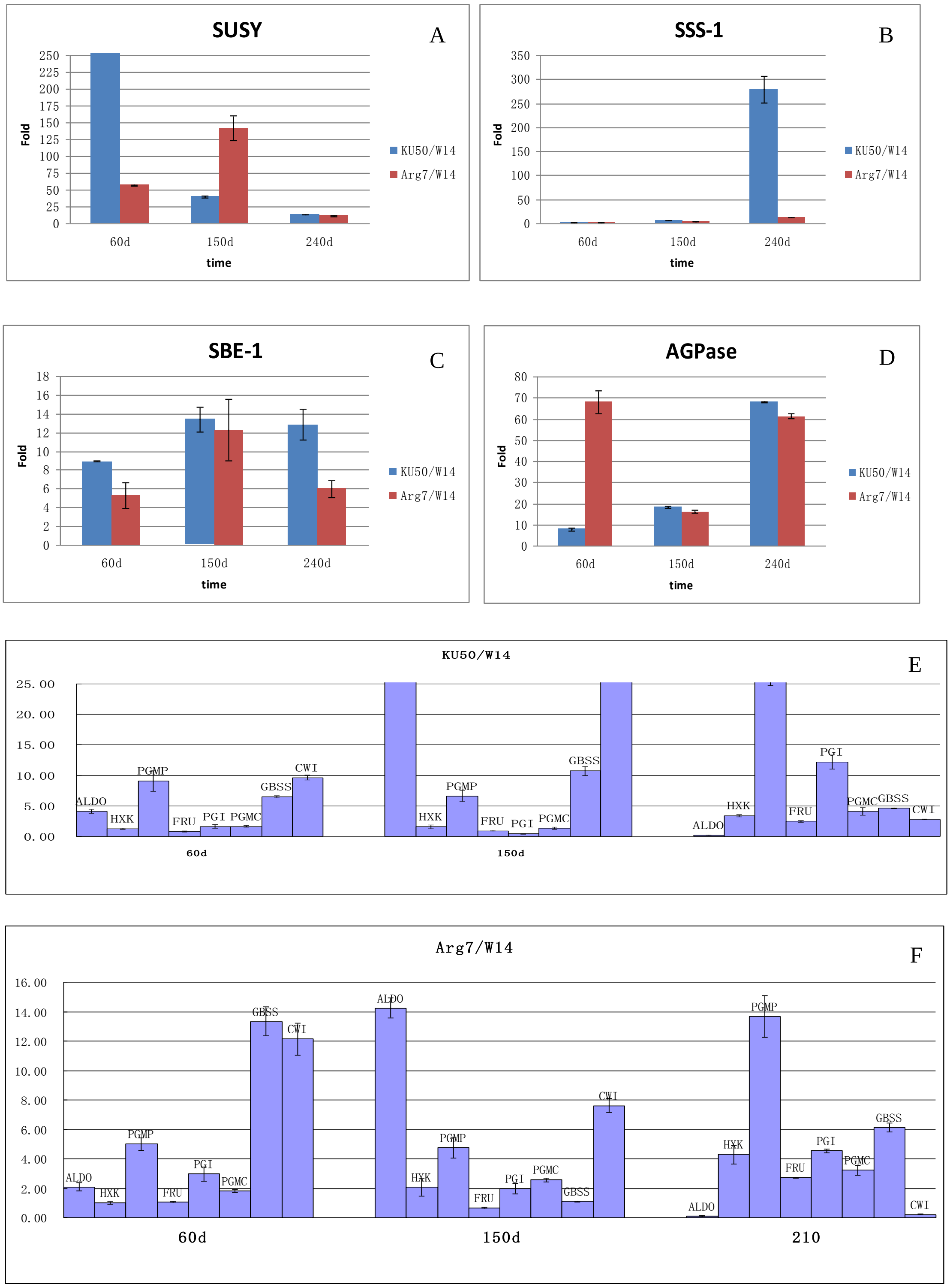

Supplementary Figure 34 RT q-PCR validation of higher expression of 12 selected genes for starch metabolism at three developmental stages of tuber root in cultivars than in wild species The comparative expression folds of KU50 and Arg7 to W14 were used for all genes. (A) SUSY with 11 to 957 folds. (B) SSS with 2.03 to 279.91 folds. (C) AGPase with 7.79 to 68.11 folds. (D) SBE with 5.28 to

13.41 folds. (E) Fold change range from 0.16 to 263.38 of KU50 to W14 with 8 genes, including *ALDO*, *HXK*, *PGMP*, *FRU*, *PGI*, *PGMC*, *GBSS* and *CWI*. (F) Fold changes ranging from 0.22 to 14.25 of Arg7 to W14 with 8 genes as the above.

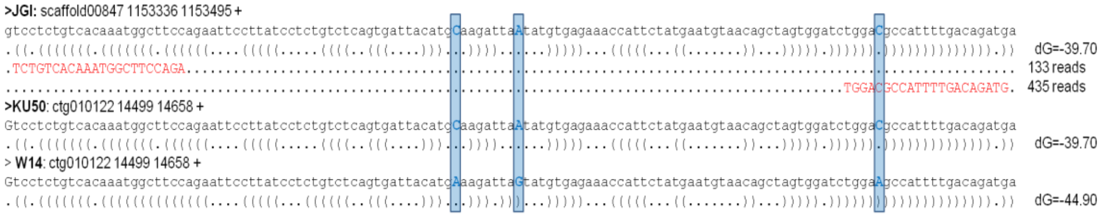

Supplementary Figure 35 miRNA novel-2 hairpins in the three cassava genomes

The genomic loci of novel-2 in the genomes are listed, followed by the hairpin sequences. The base mutations across the genomes are highlighted in the blue bars. The novel-2 mature miRNAs (red sequences) are detected in the sequencing dataset of AM560 (JGI), but not in those of KU50 and W14.

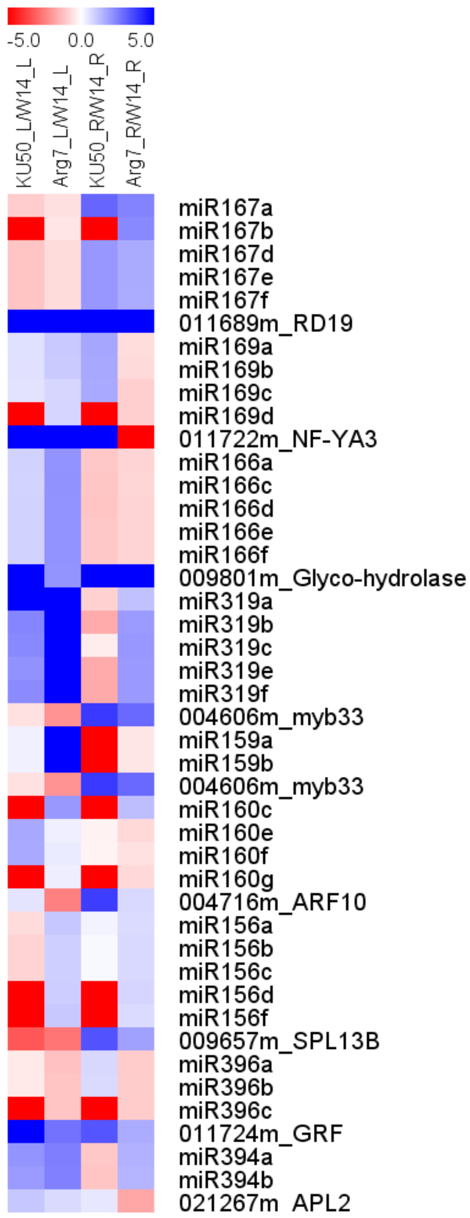

Supplementary Figure 36 Expression correlations of 9 miRNAs and their corresponding targets in leaf and tuber root of cultivars KU50 and Arg7 versus wild subspecies W14

The expressions of a majority of miRNAs showed negative correlations with their corresponding targets, except for those of miR156 and miR167. The heap map was performed on the log2 ratio of normalized expression of KU50 and Arg7 to W14 in leaf (L) and tuber root (R).

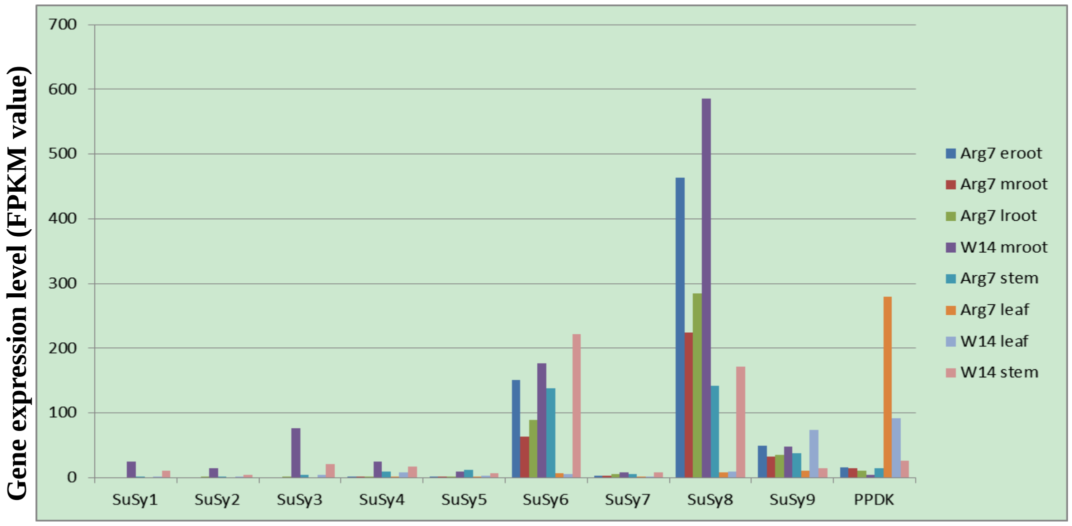

Supplementary Figure 37 Gene expression profiles of *SUSY* and *PPDK*

A

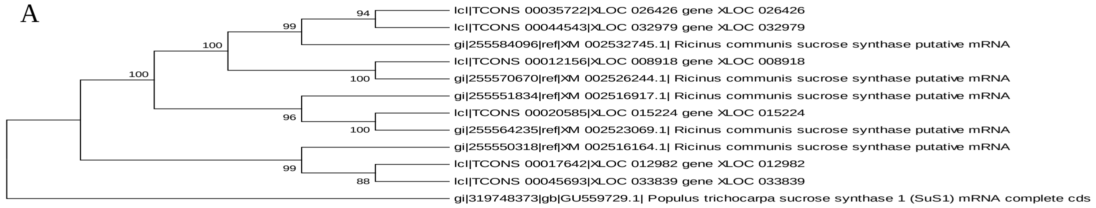

B

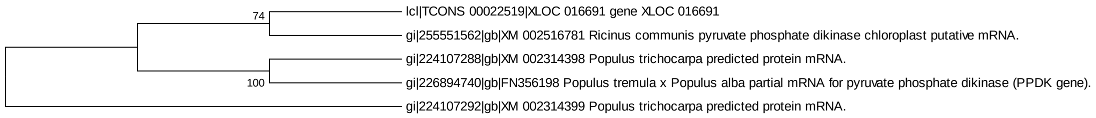

Supplementary Figure 38 Ortholog relationships among different species by MP tree

(A) MP tree of SUSY. (B) MP tree of PPDK

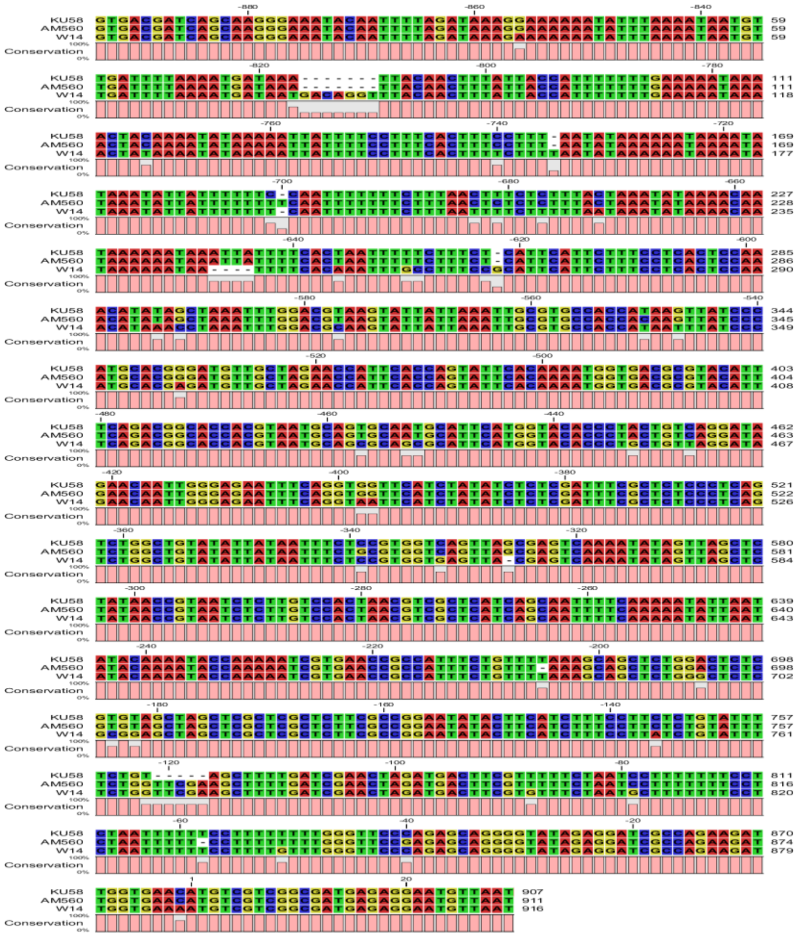

Supplementary Figure 39 Sequence alignment of the promoter region of *PPDK* in the three cassava genotypes

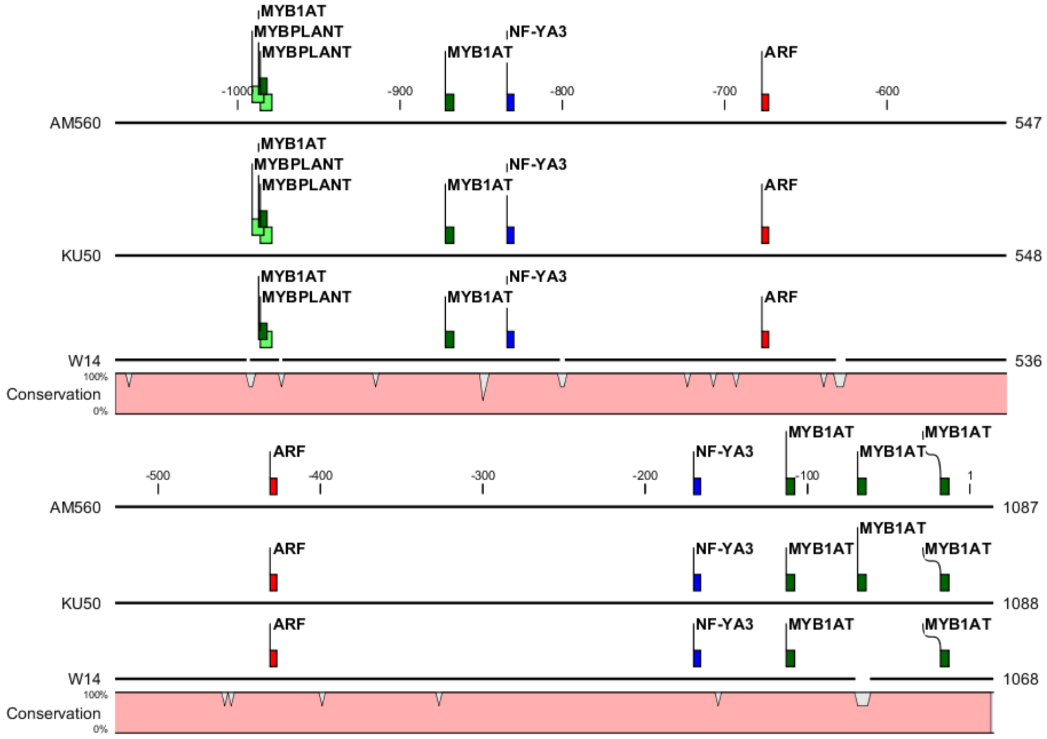

Supplementary Figure 40 Binding motifs of *MYB, ARF* and *NF-YA3* found in the upstream promoter region of *SUSY* in the genomes of wild W14 and cultivated KU50 and AM560

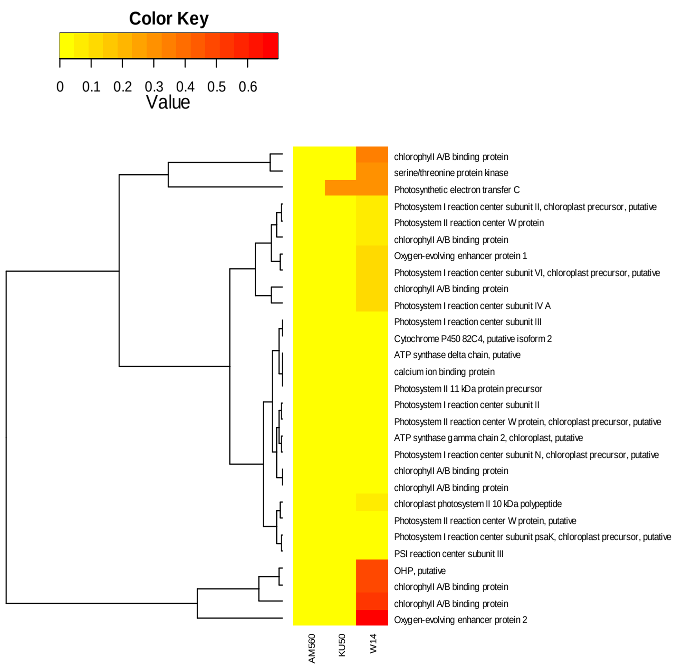

Supplementary Figure 41 Sequence comparison of 87 predicted proteins associated with light reactions between AM560, KU50 and W14

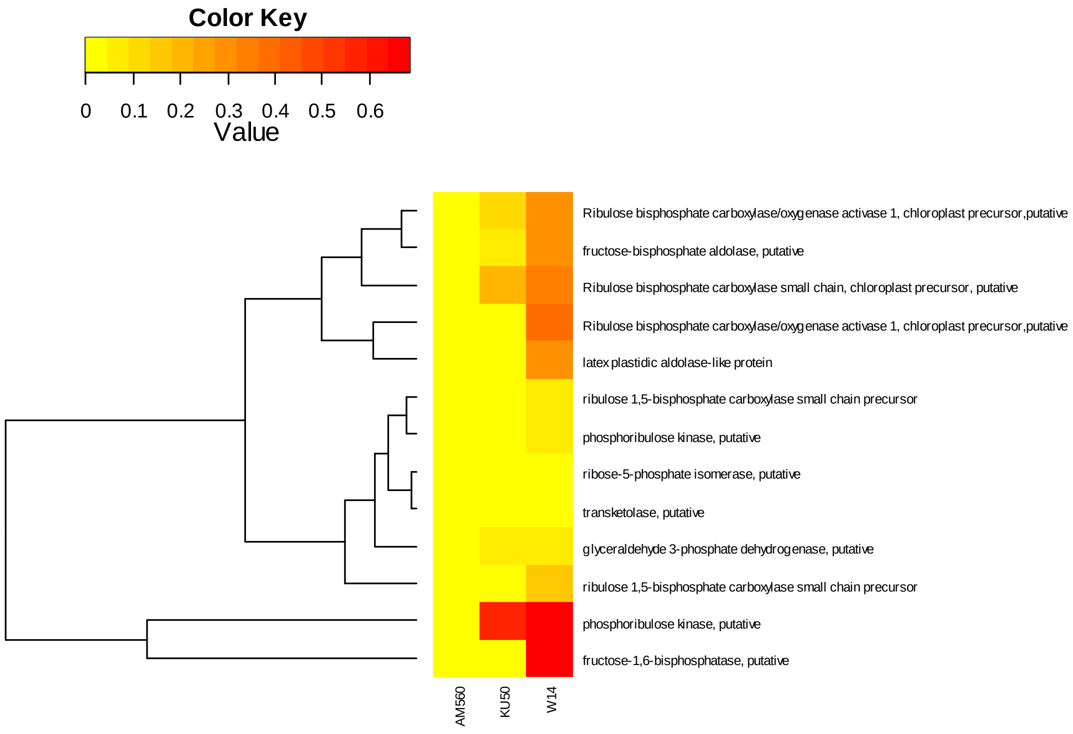

Supplementary Figure 42 Sequence comparison of 39 predicted proteins associated with Calvin cycle between AM560, KU50 and W14

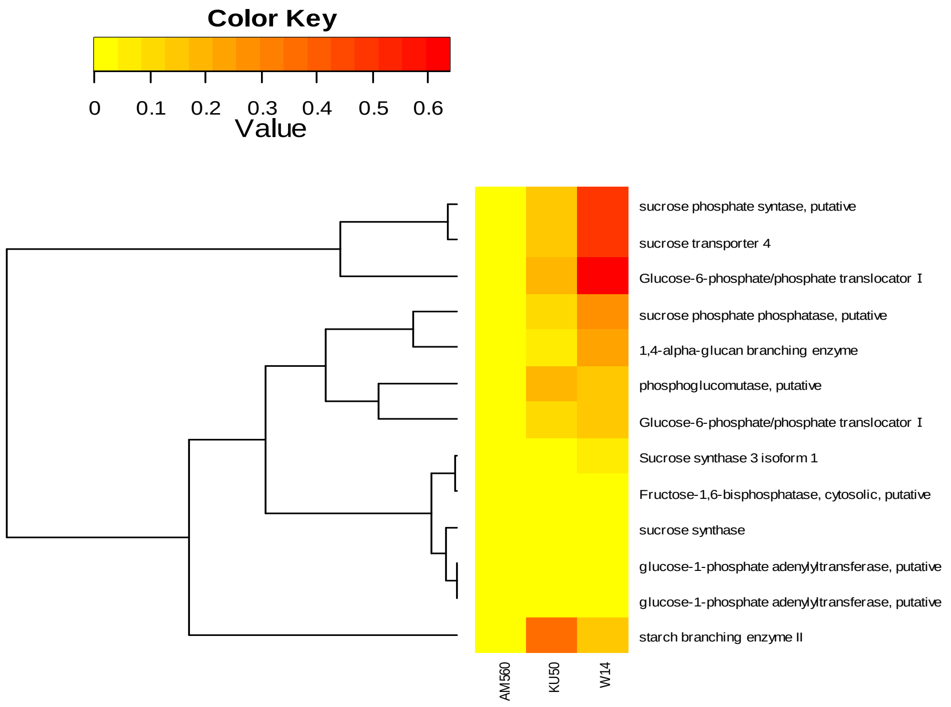

Supplementary Figure 43 Sequence comparison of 39 predicted proteins associated with synthesis of sucrose and starch between AM560, KU50 and W14

2) Supplementary Tables

Supplementary Table 1 Cassava genotypes and their characteristics used for WGS

Name	W14	KU50
Latin name	*Manihot esculenta* ssp. *flabellifolia* (Pohl) Cif.	*Manihot esculenta* ssp. *esculenta* Crantz
Fruit number	high	low
Propagation	seeds	stems
Pn (µmol/ m^2/s)	14.6 - 24.2	15.9 - 38.7
Tuber root yield (kg/plant/y)	0.5 - 2.0	3.0 - 10.0
Starch content (%)	3.0 - 5.0	28.0 - 32.0

Supplementary Table 2 Summary of BAC libraries and physical maps

Description	W14	AM560
Number of clones and genome coverage	59,904, ~10x	72,192, ~11x
Average insertion size	125 kb	115 kb
No. of clones fingerprinted	29,952	72,192
No. of high-quality fingerprints used for assembly	24,784	53,190
No. of contigs	2,485	2,105
No. of singletons	2,909	5,054
Total length of the contigs	762 Mb	793 Mb
N50 contig length	336 kb	551 kb
Longest contig	1,867 kb	4,445 kb
Average No. of clones per contig	9	25

Supplementary Table 3 Summary of genome sequencing data

W14		KU50	
Illumina Sequences			
Library	Coverage Depth (X)	Library	Coverage Depth (X)
300bp PE	14.74	200bp PE	21.75
500bp PE	27.15	300bp PE	0.37
1k-2k PE	11.72	500bp PE	2.69
4k MP	2.54	1.5k MP	8.43
8k MP	1.92	2.5k MP	5.80
10k~20k MP	1.61	4.5k MP	7.20
BP (300-500bpPE)	42.12	Total	46.25
Total	101.81		
454 Sequences			
450bp SE	1.81	20k PE	0.16
20k PE	0.18	Total	0.16
Total	1.95		

Supplementary Table 4 Resequencing genome data

Sample	Library insert size (bp)	Real insert size (bp)	PE Reads Length (bp)	Raw data			Filtered data		
				Total data (Gb)	Sequence depth (X)	Physical depth (X)	# Total data (Gb)	Sequence depth (X)	Physical depth (X)
CAS36	400	409.59	76	2.87	3.87	10.43	0.93	1.25	3.37
	400	410.13	76	3.09	4.16	11.23	1.74	2.35	6.34
	400	405.17	96	12.80	17.24	36.39	9.04	12.18	25.71
	400	403.55	96	6.23	8.39	17.64	3.96	5.34	11.22
	Total	407.11	86	24.98	33.67	79.68	15.67	21.12	50.00

Supplementary Table 5 Comparison of the W14 draft genome scaffolds with five independently sequenced BACs

BAC ID	Assembled length of BAC(bp)	Bases matched with the W14 genome (bp)	Match ratio (%)	Bases mismatched with the W14 genome (bp)	Mismatch Ratio (%)
BAC1	170,886	116,597	68.23	560	0.33
BAC2	121,938	94,481	77.48	471	0.39
BAC3	105,627	58,120	55.02	1,751	1.66
BAC4	129,136	54,365	42.10	298	0.23
BAC5	51,193	37,854	73.94	458	0.89
Average	115,756	72,283	62.44	707	0.61

Supplementary Table 6 Summary of the draft genome assembly and annotation

	W14		KU50	
	**all contigs/scafolds**	**all contigs/scafolds + mega scaffolds**	**all contigs/scafolds**	**all contigs/scafolds + mega scaffolds**
Fold of genome coverage	136	+ 8,361 BES	61	+ 43,022 BES
Total number of contigs/scaffolds	33,166	31,085	62,014	60,929
Total scalffold span	426 Mb	432 Mb	384 Mb	495 Mb
N50	33 kb	43 kb	13 kb	19 kb
Number of scaffolds	7,393	6,937	25,976	26,089
Largest scaffold	277 kb	431 kb	178 kb	335 kb
Average scaffold length	35 kb	43 kb	10 kb	15 kb
Scaffold N50	51 kb	67 kb	15 kb	27 kb
GC (%)	35.98%	35.62%	33.94%	33.68%
Gene number	34,483		38,845	
Total gene length:	92 Mb		94 Mb	
Total coding region length	41 Mb		42 Mb	
Gene density	10.37%		13.46%	
Mean length of intergenic region	5.2 kb		3.3 kb	
Minimum length of intergenic region	30 bp		655 bp	
Maximum length of	81 kb		38 kb	

intergenic region		
Total exon number	213,872	228,197
Exon number/gene	6.2	5.87
Total exon length	41 Mb	42 Mb
Mean length of exons	189.57 bp	183.54 bp
Minimum length of exon	3 bp	3 bp
Maximum length of exons	9 kb	8 kb
GC contentof exons	43.40%	43.36%
Total intron number	179,389	189,352
Intron number/gene	5.2	4.87
Total intron length	51 Mb	50 Mb
Mean length of Introns	350.76 bp	263.97 bp
Minimum length of introns	21 bp	21 bp
Maximum length of introns	19 kb	16 kb
GC content of introns	32.86%	32.88%

Supplementary Table 7 Functional gene annotation statistics

		W14		KU50	
	Database	Number 34,483	Percentage 100 (%)	Number 38,845	Percentage 100 (%)
Annotated	Swissprot	20,493	59.43%	22,861	58.85%
	TrEMBL	28,889	83.78%	33,029	85.03%
	InterPro/GO	24,663	71.52%	27,510	70.82%
	KEGG	25,367	73.56%	28,794	74.13%
	COG	12,017	34.85%	13,164	33.89%
	Pfam	26,587	77.10%	30,084	77.45%
	NR/NT	33,203	96.29%	37,477	96.48%
Annotated		33,310	96.60%	37,592	96.77%
Un-annotated		1,173	3.40%	1,253	3.23%

Supplementary Table 8 Summary of repetitive sequences in the KU50 and W14 genome assemblies

Repeat classes	Number of elements		Length occupied (bp)		Percentage of sequence (%)	
	KU50	W14	KU50	W14	KU50	W14
SINEs	331	273	34232	30083	0.01	0.01
LINEs	8931	17825	3526665	6434258	0.85	1.35
LTR elements	94004	100522	46408472	55903649	11.14	11.76
DNA elements	12517	26880	2953474	9377558	0.71	1.97
Unclassified	209815	364501	48560940	89444202	11.66	18.82
Simple repeats	19250	45307	1015255	2121623	0.24	0.45
Low complexity	80646	166835	4903664	11734724	1.18	2.47

Supplementary Table 9 Self-diversity evaluation of the three draft cassava genomes

Sample	W14	KU50	AM560
**# SNVs**	1,377,370	806,271	506,746
**SNVs density (# SNVs/kb)**	3.89	3.50	1.44
**# SNVs in genes**	295,358	109,701	73,628
**SNV density in genes (# SNVs/kb)**	3.70	2.98	0.16
**# SNVs in exons**	220,600	43,610	46,524
**SNV density in exons (# SNVs/kb)**	3.68	2.37	0.18
**# SNVs in intergenic regions**	1,082,082	806,149	433,118
**SNV density in intergenic regions (# SNVs/kb)**	3.31	3.50	1.27
**# SNVs in repeat regions**	796,028	476,739	393,831
**SNV density in repeat regions (# SNVs/kb)**	3.32	3.60	1.18

Supplementary Table 10 Summary of SNVs among three cassava genomes

Samples	W14	KU50	CAS36
# SNVs	4,812,287	3,620,860	2,977,198
SNV density (# SNVs/kb)	6.94	4.57	4.10
# SNVs in genes	1,574,460	516,278	517,321
SNV density in genes (# SNVs/kb)	3.40	1.12	1.12
# SNVs in exons	563,588	187,122	186,413
SNV density in exons (# SNVs/kb)	1.48	0.51	0.52
# SNVs in intergenic regions	3,237,827	3,104,582	2,459,877
SNV density in intergenic regions (# SNVs/kb)	6.25	4.37	3.92
SNVs in repeat regions	1,751,276	2,142,290	1,737,544
SNV density in repeat regions (# SNVs/kb)	1/214	1/274	1/294

Supplementary Table 11 Summary of InDels among three cassava genomes

Samples	W14	KU50	CAS36
**# InDels**	390,652	275,639	217,226
**InDel density (# Indels/kb)**	0.80	0.79	0.64
**# insertion**	159,467	132,396	103,964
**# deletion**	231,080	143,200	113,207
**average length (bp)**	3.59	3.65	4.07
**minium length (bp)**	1	1	1
**maximum length (bp)**	89	86	109
**# InDels in genes**	156,096	61,946	59,361
**# InDels in exons**	22,717	9,477	8,938
**# InDels in intergenic regions**	211,839	204,216	148,927
**# InDels in repeat regions**	74,467	96,390	72,873

Supplementary Table 12 Statistics of insertion and deletion in cultivars KU50 and AM560 relative to the wild ancestor subspecies W14

	Insertions	Intron	Exon	Intron & exon	Insertion sum (bp)
KU50	610	583	18	9	120,969
AM560	614	584	17	13	126,882
	Deletions	Intron	Exon	Intron & exon	Deletion sum (bp)
KU50	797	685	40	72	186,906
AM560	784	670	41	73	179,543

Supplementary Table 13 The synonymous (*Ks*) and nonsynonymous (*Ka*) divergence values and selective pressure (*Ka/Ks*) among the genomes of KU50, AM560 and W14 determined with 16,219 high-confidence 1:1:1 orthologous genes

	**W14_vs_KU50**	**W14_vs_AM560**	**KU50_vs_AM560**
Ka **(average)**	0.106675	0.082961	0.061529
Ks **(average)**	0.190331	0.154327	0.098463
Ka/Ks	0.560469	0.537566	0.624900
Ka+Ks **(average)**	0.297006	0.237288	0.159992

Supplementary Table 14 **Statistics of numbers and frequency of genes subjected to natural and artificial selection under low selective pressures during domestication**

	**W14_vs_AM560**		**W14_vs_KU50**		**KU50_vs_AM560**	
**Gene Number**	12973		10978		13170	
Ka=**0**	295	2.27%	269	2.45%	4978	37.80%
Ks=**0**	146	1.13%	182	1.66%	4682	35.55%
Ka+Ks=**0**	20	0.15%	28	0.26%	3318	25.19%
Ka/Ks(**log2)<-5**	356	2.74%	307	2.80%	1664	12.63%
Ka/Ks(**log2)>2**	144	1.11%	175	1.59%	1413	10.72%

Supplementary Table 15 Difference of *Ka*, *Ks* and *Ka/Ks* between cultivars and wild subspecies in the 4,982 genes that have very low selective pressure in cultivars and have been strictly selected for during domestication

	W14_vs_KU50	W14_vs_AM560	KU50_vs_AM560
Ka (average)	0.079137	0.078992	0.000001
Ks (average)	0.143521	0.143358	0.003402
Ka/Ks	0.535344	0.534884	0.000472

Supplementary Table 16 Summary of RNA-seq raw reads mapped and annotated transcripts

	W14 leaf (DL)	W14 stem (DS)	W14 root (MTR)	Arg7 leaf (DF)	Arg7 Stem (DS)	Arg7 early root (ETR)	Arg7 middle root (MTR)	Arg7 later root (LTR)	KU50 leaf (DL)	KU50 early root (ETR)	KU50 middle root (MTR)	KU50 later root (LTR)
Reads	68,673,876	13,359,172	9,693,871	30,710,363	29,605,379	12,034,644	15,087,006	39,221,907	29,905,212	32,700,866	34,029,215	32,255,360
Qualified reads	64,966,332	12,886,906	9,373,887	29,396,575	28,610,467	11,694,780	14,400,150	37,469,237	29,820,379	32,534,593	33,936,178	32,161,203
Mapped reads	37,086,555	5,759,129	7,714,331	25,342,750	24,330,092	8,717,932	12,637,538	32,240,630	22,409,073	29,201,102	30,934,931	28,868,179
Percentage of reads mapped	57.09%	44.69%	82.30%	86.21%	85.04%	74.55%	87.76%	86.05%	75.15%	89.75%	91.16%	89.76%
Expressed transcripts	53,715	46,698	38,965	43,023	46,439	41,461	40,868	45,294	51,300	50,334	49,913	48,358
Unique genes annotated	16,884	19,533	23,379	21,378	18,949	21,776	22,757	19,253	17,680	16,755	17,112	17,641
Mean length of unique genes	2,004.51	2,189.61	2,631.95	2,483.69	2,285.32	2,472.60	2,509.36	2,240.38	2,076.99	2,082.30	2,101.68	2,157.17

Note: Reference: AM560-2 (phyztome v7 assembly Mesculenta_147_RM), Alignment: bowtie2 v2.1.0/TopHat v2.0.9 Alignment: bowtie V2.1/TopHat V2.0.9; DiffExp: cuffdiff V2.1.1

Supplementary Table 17 Summary of networks of GO terms over-represented in functional leaf and storage root of wild and cultivated cassava

Group	Network[a]	GO term[b]	Number (%) in DEGP group	Number (%) in background[c]	P-value
W14 Leaf	1(C)	└ endomembrane system	29(14.87%)	2482(8.99%)	4.96E-03
		└ membrane	46(23.59%)	3727(13.51%)	9.30E-05
		└ plasma membrane	26(13.33%)	1574(5.70%)	4.97E-05
		└ cell	114(58.46%)	11708(42.43%)	4.48E-06
		└ cell part	114(58.46%)	11708(42.43%)	4.48E-06
	2(F)	└ transporter activity	20(10.26%)	1125(4.08%)	1.50E-04
		└ transmembrane transporter activity	18(9.23%)	840(3.04%)	3.15E-05
		└ active transmembrane transporter activity	14(7.18%)	501(1.82%)	1.45E-05
		└ secondary active transmembrane transporter activity	10(5.13%)	259(0.94%)	1.69E-05
		└ symporter activity	6(3.08%)	118(0.43%)	1.97E-04
		└ solute:cation symporter activity	5(2.56%)	117(0.42%)	1.48E-03
		└ solute:hydrogen symporter activity	4(2.05%)	90(0.33%)	3.85E-03
		└ cation:sugar symporter activity	4(2.05%)	90(0.33%)	3.85E-03
		└ sugar:hydrogen symporter activity	4(2.05%)	90(0.33%)	3.85E-03
		└ potassium ion symporter activity	1(0.51%)	1(0.00%)	7.07E-03
		└ antiporter activity	4(2.05%)	131(0.47%)	1.41E-02
		└ ATPase activity, coupled to transmembrane movement of ions	3(1.54%)	48(0.17%)	4.76E-03
		└ substrate-specific transmembrane transporter activity	15(7.69%)	683(2.48%)	1.12E-04
		└ ion transmembrane transporter activity	12(6.15%)	489(1.77%)	2.01E-04

	└ ATPase activity, coupled to transmembrane movement of ions, phosphorylative mechanism	3(1.54%)	35(0.13%)	1.93E-03
	└ cation transmembrane transporter activity	10(5.13%)	369(1.34%)	3.15E-04
	└ cation-transporting ATPase activity	2(1.03%)	31(0.11%)	2.02E-02
	└ potassium:sodium symporter activity	1(0.51%)	1(0.00%)	7.07E-03
	└ calcium ion transmembrane transporter activity	2(1.03%)	17(0.06%)	6.30E-03
	└ calcium-transporting ATPase activity	2(1.03%)	16(0.06%)	5.59E-03
	└ inorganic anion transmembrane transporter activity	3(1.54%)	60(0.22%)	8.85E-03
	└ carbohydrate transmembrane transporter activity	4(2.05%)	105(0.38%)	6.64E-03
	└ sugar transmembrane transporter activity	4(2.05%)	97(0.35%)	5.02E-03
	└ substrate-specific transporter activity	15(7.69%)	796(2.88%)	5.68E-04
3(P)	└ response to stimulus	54(27.69%)	3207(11.62%)	6.80E-10
	└ response to chemical stimulus	28(14.36%)	1710(6.20%)	2.90E-05
	└ response to organic substance	18(9.23%)	1037(3.76%)	4.30E-04
	└ response to ATP	1(0.51%)	1(0.00%)	7.07E-03
	└ auxin efflux	1(0.51%)	2(0.01%)	1.41E-02
	└ cellular response to auxin stimulus	2(1.03%)	33(0.12%)	2.27E-02
	└ response to brassinosteroid stimulus	3(1.54%)	53(0.19%)	6.28E-03
	└ response to salicylic acid stimulus	5(2.56%)	135(0.49%)	2.77E-03
	└ response to cadmium ion	6(3.08%)	279(1.01%)	1.47E-02
	└ response to stress	29(14.87%)	1853(6.72%)	4.66E-05
	└ defense response	13(6.67%)	637(2.31%)	6.40E-04
	└ response to oxidative stress	6(3.08%)	247(0.90%)	8.42E-03
	└ response to wounding	8(4.10%)	133(0.48%)	4.97E-06
	└ response to biotic stimulus	12(6.15%)	550(1.99%)	5.78E-04
	└ response to other organism	11(5.64%)	528(1.91%)	1.41E-03

	└ response to abiotic stimulus	18(9.23%)	1168(4.23%)	1.66E-03
	└ response to radiation	11(5.64%)	471(1.71%)	5.60E-04
	└ response to light stimulus	11(5.64%)	455(1.65%)	4.20E-04
	└ response to UV	5(2.56%)	65(0.24%)	9.80E-05
	└ photoperiodism	3(1.54%)	39(0.14%)	2.63E-03
	└ entrainment of circadian clock by photoperiod	1(0.51%)	3(0.01%)	2.11E-02
	└ response to endogenous stimulus	13(6.67%)	835(3.03%)	6.65E-03
	└ response to hormone stimulus	11(5.64%)	767(2.78%)	2.09E-02
	└ response to jasmonic acid stimulus	4(2.05%)	148(0.54%)	2.11E-02
4(P)	└ biological regulation	41(21.03%)	3243(11.75%)	1.47E-04
	└ regulation of biological process	33(16.92%)	2783(10.09%)	2.13E-03
	└ regulation of metabolic process	23(11.79%)	1825(6.61%)	5.11E-03
	└ regulation of cellular metabolic process	23(11.79%)	1664(6.03%)	1.63E-03
	└ regulation of nucleobase, nucleoside, nucleotide and nucleic acid metabolic process	20(10.26%)	1527(5.53%)	5.92E-03
	└ regulation of transcription	19(9.74%)	1468(5.32%)	8.19E-03
	└ regulation of RNA metabolic process	12(6.15%)	813(2.95%)	1.32E-02
	└ regulation of transcription, DNA-dependent	12(6.15%)	810(2.94%)	1.29E-02
	└ negative regulation of flavonoid biosynthetic process	1(0.51%)	1(0.00%)	7.07E-03
	└ regulation of flavonoid biosynthetic process	2(1.03%)	15(0.05%)	4.91E-03
	└ regulation of secondary metabolic process	2(1.03%)	30(0.11%)	1.90E-02
	└ regulation of nitrogen compound metabolic process	20(10.26%)	1545(5.60%)	6.71E-03
	└ regulation of gene expression	19(9.74%)	1642(5.95%)	2.39E-02
	└ regulation of primary metabolic process	22(11.28%)	1604(5.81%)	2.26E-03
	└ regulation of biosynthetic process	21(10.77%)	1540(5.58%)	3.05E-03
	└ regulation of macromolecule biosynthetic process	19(9.74%)	1504(5.45%)	1.04E-02

└ regulation of cellular biosynthetic process	21(10.77%)	1540(5.58%)	3.05E-03
└ negative regulation of biosynthetic process	3(1.54%)	76(0.28%)	1.68E-02
└ negative regulation of cellular biosynthetic process	3(1.54%)	76(0.28%)	1.68E-02
└ positive regulation of biological process	5(2.56%)	218(0.79%)	1.96E-02
└ positive regulation of response to external stimulus	1(0.51%)	3(0.01%)	2.11E-02
└ positive regulation of developmental process	3(1.54%)	50(0.18%)	5.34E-03
└ positive regulation of post-embryonic development	3(1.54%)	43(0.16%)	3.48E-03
└ positive regulation of response to extracellular stimulus	1(0.51%)	3(0.01%)	2.11E-02
└ positive regulation of response to nutrient levels	1(0.51%)	3(0.01%)	2.11E-02
└ positive regulation of cellular response to phosphate starvation	1(0.51%)	3(0.01%)	2.11E-02
└ positive regulation of flower development	3(1.54%)	34(0.12%)	1.77E-03
└ regulation of cellular process	29(14.87%)	2448(8.87%)	4.09E-03
└ regulation of biological quality	9(4.62%)	569(2.06%)	2.03E-02
└ regulation of hormone levels	5(2.56%)	124(0.45%)	1.91E-03
└ auxin transport	3(1.54%)	50(0.18%)	5.34E-03
└ cell volume homeostasis	1(0.51%)	2(0.01%)	1.41E-02
└ cellular water homeostasis	1(0.51%)	1(0.00%)	7.07E-03
└ secondary metabolic process	24(12.31%)	330(1.20%)	1.69E-17
└ diterpenoid metabolic process	2(1.03%)	24(0.09%)	1.24E-02
└ diterpenoid biosynthetic process	2(1.03%)	18(0.07%)	7.06E-03
└ gibberellin metabolic process	2(1.03%)	23(0.08%)	1.14E-02
└ gibberellin biosynthetic process	2(1.03%)	17(0.06%)	6.30E-03
└ terpenoid biosynthetic process	3(1.54%)	70(0.25%)	1.35E-02
└ phenylpropanoid metabolic process	18(9.23%)	133(0.48%)	3.40E-18
└ phenylpropanoid biosynthetic process	16(8.21%)	104(0.38%)	3.19E-17

		└ chalcone metabolic process	1(0.51%)	1(0.00%)	7.07E-03
		└ cinnamic acid metabolic process	1(0.51%)	1(0.00%)	7.07E-03
		└ flavonoid metabolic process	13(6.67%)	51(0.18%)	2.75E-17
		└ anthocyanin metabolic process	3(1.54%)	15(0.05%)	1.49E-04
		└ flavone metabolic process	2(1.03%)	8(0.03%)	1.35E-03
		└ flavonoid biosynthetic process	12(6.15%)	46(0.17%)	3.48E-16
	5(P)	└ cellular metabolic process	58(29.74%)	5407(19.59%)	4.34E-04
		└ cellular amino acid and derivative metabolic process	19(9.74%)	483(1.75%)	1.85E-09
		└ cellular amino acid derivative metabolic process	19(9.74%)	231(0.84%)	4.75E-15
		└ cellular amino acid derivative biosynthetic process	17(8.72%)	171(0.62%)	5.89E-15
Ku50-Arg7 Leaf	1(C)	└ light-harvesting complex	4(1.53%)	22(0.08%)	5.00E-05
		└ 1-phosphatidylinositol-4-phosphate 3-kinase, class IA complex	1(0.38%)	1(0.00%)	9.46E-03
		└ photosystem	10(3.83%)	38(0.14%)	1.81E-12
		└ photosystem I reaction center	2(0.77%)	8(0.03%)	2.40E-03
		└ organelle	89(34.10%)	6091(22.07%)	5.14E-06
		└ membrane-bounded organelle	89(34.10%)	5767(20.90%)	4.71E-07
		└ organelle envelope	16(6.13%)	601(2.18%)	2.15E-04
		└ organelle subcompartment	22(8.43%)	256(0.93%)	5.70E-15
		└ plastid thylakoid	22(8.43%)	254(0.92%)	4.84E-15
		└ plastid thylakoid lumen	6(2.30%)	58(0.21%)	1.81E-05
		└ plastid thylakoid membrane	21(8.05%)	211(0.76%)	1.26E-15
		└ chloroplast thylakoid	22(8.43%)	254(0.92%)	4.84E-15
		└ chloroplast thylakoid membrane	21(8.05%)	211(0.76%)	1.26E-15
		└ chloroplast thylakoid lumen	6(2.30%)	58(0.21%)	1.81E-05
		└ intracellular membrane-bounded organelle	89(34.10%)	5766(20.90%)	4.67E-07

└ plastid	52(19.92%)	2139(7.75%)	2.38E-10
└ plastid part	33(12.64%)	782(2.83%)	7.64E-13
└ chloroplast	51(19.54%)	2070(7.50%)	2.31E-10
└ chloroplast part	33(12.64%)	755(2.74%)	2.92E-13
└ intracellular organelle	89(34.10%)	6090(22.07%)	5.10E-06
└ intracellular organelle part	37(14.18%)	1970(7.14%)	5.09E-05
└ organelle part	37(14.18%)	1972(7.15%)	5.20E-05
└ membrane	54(20.69%)	3727(13.51%)	8.34E-04
└ photosynthetic membrane	22(8.43%)	227(0.82%)	4.56E-16
└ thylakoid membrane	21(8.05%)	224(0.81%)	4.23E-15
└ photosystem I	6(2.30%)	15(0.05%)	3.15E-09
└ chloroplast photosystem I	2(0.77%)	3(0.01%)	2.66E-04
└ photosystem II	4(1.53%)	23(0.08%)	6.01E-05
└ chloroplast photosystem II	4(1.53%)	17(0.06%)	1.69E-05
└ membrane part	22(8.43%)	1098(3.98%)	8.10E-04
└ envelope	16(6.13%)	601(2.18%)	2.15E-04
└ plastid envelope	15(5.75%)	382(1.38%)	4.14E-06
└ chloroplast envelope	15(5.75%)	361(1.31%)	2.08E-06
└ thylakoid lumen	6(2.30%)	74(0.27%)	7.31E-05
└ plastoglobule	3(1.15%)	55(0.20%)	1.53E-02
└ plastid stroma	10(3.83%)	354(1.28%)	2.12E-03
└ chloroplast stroma	9(3.45%)	335(1.21%)	4.84E-03
└ cytoplasm	73(27.97%)	4745(17.20%)	9.35E-06
└ cytoplasmic part	68(26.05%)	4323(15.67%)	1.03E-05
└ thylakoid	26(9.96%)	322(1.17%)	7.99E-17
└ thylakoid part	23(8.81%)	266(0.96%)	1.18E-15

	└ intracellular	101(38.70%)	7208(26.12%)	5.35E-06
	└ intracellular part	96(36.78%)	6908(25.03%)	1.56E-05
	└ extracellular region	10(3.83%)	393(1.42%)	4.45E-03
	└ cell	143(54.79%)	11708(42.43%)	3.61E-05
	└ cell part	143(54.79%)	11708(42.43%)	3.61E-05
2(P)	└ regulation of metabolic process	29(11.11%)	1825(6.61%)	4.38E-03
	└ regulation of cellular metabolic process	29(11.11%)	1664(6.03%)	1.14E-03
	└ positive regulation of cellular amino acid metabolic process	1(0.38%)	1(0.00%)	9.46E-03
	└ regulation of tryptophan metabolic process	1(0.38%)	1(0.00%)	9.46E-03
	└ positive regulation of tryptophan metabolic process	1(0.38%)	1(0.00%)	9.46E-03
	└ regulation of nucleobase, nucleoside, nucleotide and nucleic acid metabolic process	25(9.58%)	1527(5.53%)	5.56E-03
	└ regulation of transcription	25(9.58%)	1468(5.32%)	3.38E-03
	└ positive regulation of cellular amine metabolic process	1(0.38%)	1(0.00%)	9.46E-03
	└ regulation of nitrogen compound metabolic process	26(9.96%)	1545(5.60%)	3.30E-03
	└ regulation of macromolecule metabolic process	27(10.34%)	1685(6.11%)	5.31E-03
	└ regulation of gene expression	27(10.34%)	1642(5.95%)	3.78E-03
	└ regulation of primary metabolic process	28(10.73%)	1604(5.81%)	1.36E-03
	└ regulation of biosynthetic process	27(10.34%)	1540(5.58%)	1.56E-03
	└ regulation of macromolecule biosynthetic process	26(9.96%)	1504(5.45%)	2.30E-03
	└ regulation of cellular biosynthetic process	27(10.34%)	1540(5.58%)	1.56E-03
	└ positive regulation of biosynthetic process	4(1.53%)	66(0.24%)	3.56E-03
	└ positive regulation of cellular biosynthetic process	4(1.53%)	66(0.24%)	3.56E-03
	└ positive regulation of metabolic process	5(1.92%)	81(0.29%)	1.04E-03
	└ positive regulation of cellular metabolic process	5(1.92%)	78(0.28%)	8.76E-04

		└ positive regulation of nitrogen compound metabolic process	4(1.53%)	64(0.23%)	3.19E-03
		└ positive regulation of cellular process	5(1.92%)	137(0.50%)	9.91E-03
		└ regulation of cellular process	35(13.41%)	2448(8.87%)	9.19E-03
		└ regulation of stomatal closure	1(0.38%)	1(0.00%)	9.46E-03
	3(P)	└ photosynthesis	15(5.75%)	113(0.41%)	2.30E-13
		└ photosynthesis, light harvesting in photosystem I	3(1.15%)	4(0.01%)	3.32E-06
		└ photosynthetic electron transport chain	3(1.15%)	25(0.09%)	1.65E-03
		└ photosynthetic electron transport in photosystem I	2(0.77%)	15(0.05%)	8.63E-03
		└ photosynthetic NADP+ reduction	1(0.38%)	1(0.00%)	9.46E-03
		└ tetrahydrobiopterin biosynthetic process	1(0.38%)	1(0.00%)	9.46E-03
		└ tetrahydrobiopterin metabolic process	1(0.38%)	1(0.00%)	9.46E-03
		└ cellular aldehyde metabolic process	3(1.15%)	20(0.07%)	8.47E-04
		└ glyoxylate metabolic process	2(0.77%)	3(0.01%)	2.66E-04
		└ generation of precursor metabolites and energy	9(3.45%)	199(0.72%)	1.25E-04
		└ photosynthesis, light reaction	8(3.07%)	63(0.23%)	1.42E-07
		└ electron transport chain	4(1.53%)	56(0.20%)	1.95E-03
		└ photosynthesis, light harvesting	4(1.53%)	21(0.08%)	4.12E-05
W14 Root	1(C)	└ external encapsulating structure	39(5.44%)	462(1.67%)	1.40E-10
		└ cell wall	39(5.44%)	458(1.66%)	1.08E-10
		└ plant-type cell wall	20(2.79%)	180(0.65%)	5.43E-08
	2(F)	└ catalytic activity	294(41.00%)	7553(27.37%)	1.02E-15
		└ oxidoreductase activity	79(11.02%)	1326(4.81%)	5.98E-12
		└ steroid dehydrogenase activity, acting on the CH-OH group of donors, NAD or NADP as acceptor	2(0.28%)	7(0.03%)	1.30E-02
		└ 3-beta-hydroxy-delta5-steroid dehydrogenase activity	2(0.28%)	7(0.03%)	1.30E-02
		└ oxidoreductase activity, acting on CH-OH group of donors	10(1.39%)	139(0.50%)	3.49E-03

└ oxidoreductase activity, acting on the CH-OH group of donors, NAD or NADP as acceptor	10(1.39%)	114(0.41%)	7.93E-04
└ pinoresinol reductase activity	2(0.28%)	3(0.01%)	1.99E-03
└ oxidoreductase activity, acting on the CH-NH2 group of donors, oxygen as acceptor	4(0.56%)	36(0.13%)	1.38E-02
└ oxidoreductase activity, acting on diphenols and related substances as donors	5(0.70%)	35(0.13%)	1.99E-03
└ oxidoreductase activity, acting on diphenols and related substances as donors, oxygen as acceptor	5(0.70%)	27(0.10%)	5.87E-04
└ laccase activity	4(0.56%)	16(0.06%)	6.41E-04
└ oxidoreductase activity, acting on paired donors, with incorporation or reduction of molecular oxygen	14(1.95%)	154(0.56%)	5.22E-05
└ oxidoreductase activity, acting on paired donors, with incorporation or reduction of molecular oxygen, 2-oxoglutarate as one donor, and incorporation of one atom each of oxygen into both donors	6(0.84%)	70(0.25%)	9.68E-03
└ oxidoreductase activity, acting on paired donors, with oxidation of a pair of donors resulting in the reduction of molecular oxygen to two molecules of water	3(0.42%)	12(0.04%)	3.23E-03
└ monooxygenase activity	21(2.93%)	300(1.09%)	4.37E-05
└ transferase activity	93(12.97%)	2429(8.80%)	1.01E-04
└ acetolactate synthase activity	2(0.28%)	3(0.01%)	1.99E-03
└ transferase activity, transferring glycosyl groups	27(3.77%)	416(1.51%)	1.47E-05
└ transferase activity, transferring hexosyl groups	22(3.07%)	283(1.03%)	5.65E-06
└ glucosyltransferase activity	10(1.39%)	125(0.45%)	1.60E-03
└ UDP-glycosyltransferase activity	13(1.81%)	171(0.62%)	5.56E-04

	└ glucuronosyltransferase activity	4(0.56%)	9(0.03%)	5.13E-05
	└ nucleoside kinase activity	2(0.28%)	5(0.02%)	6.40E-03
	└ lyase activity	19(2.65%)	304(1.10%)	4.21E-04
3(P)	└ response to stimulus	121(16.88%)	3207(11.62%)	1.53E-05
	└ response to chemical stimulus	72(10.04%)	1710(6.20%)	3.86E-05
	└ response to organic substance	43(6.00%)	1037(3.76%)	1.90E-03
	└ response to carbohydrate stimulus	13(1.81%)	177(0.64%)	7.69E-04
	└ response to disaccharide stimulus	6(0.84%)	36(0.13%)	3.02E-04
	└ response to sucrose stimulus	6(0.84%)	35(0.13%)	2.57E-04
	└ response to metal ion	17(2.37%)	350(1.27%)	1.07E-02
	└ response to cadmium ion	15(2.09%)	279(1.01%)	6.66E-03
	└ cellular response to xenobiotic stimulus	2(0.28%)	4(0.01%)	3.91E-03
	└ response to biotic stimulus	34(4.74%)	550(1.99%)	3.47E-06
	└ response to other organism	30(4.18%)	528(1.91%)	6.18E-05
	└ multi-organism process	34(4.74%)	694(2.52%)	3.50E-04
4(P)	└ response to osmotic stress	20(2.79%)	388(1.41%)	3.10E-03
	└ response to salt stress	19(2.65%)	360(1.30%)	3.00E-03
	└ response to desiccation	4(0.56%)	18(0.07%)	1.03E-03
	└ response to wounding	10(1.39%)	133(0.48%)	2.54E-03
	└ response to abiotic stimulus	46(6.42%)	1168(4.23%)	3.58E-03
5(P)	└ metabolic process	208(29.01%)	6834(24.77%)	4.86E-03
	└ secondary metabolic process	35(4.88%)	330(1.20%)	2.26E-12
	└ phenylpropanoid metabolic process	28(3.91%)	133(0.48%)	8.43E-18
	└ phenylpropanoid biosynthetic process	22(3.07%)	104(0.38%)	2.52E-14
	└ coumarin metabolic process	3(0.42%)	3(0.01%)	1.75E-05
	└ lignan metabolic process	4(0.56%)	16(0.06%)	6.41E-04

└ lignan biosynthetic process	4(0.56%)	16(0.06%)	6.41E-04
└ lignin metabolic process	8(1.12%)	44(0.16%)	1.55E-05
└ lignin biosynthetic process	6(0.84%)	28(0.10%)	6.97E-05
└ flavonoid metabolic process	11(1.53%)	51(0.18%)	6.25E-08
└ anthocyanin metabolic process	4(0.56%)	15(0.05%)	4.91E-04
└ flavonoid biosynthetic process	10(1.39%)	46(0.17%)	2.31E-07
└ pigment metabolic process	7(0.98%)	92(0.33%)	1.01E-02
└ anthocyanin biosynthetic process	3(0.42%)	11(0.04%)	2.47E-03
└ cell wall macromolecule metabolic process	10(1.39%)	41(0.15%)	7.14E-08
└ cellular cell wall macromolecule metabolic process	5(0.70%)	14(0.05%)	1.92E-05
└ cell wall macromolecule biosynthetic process	4(0.56%)	12(0.04%)	1.89E-04
└ cell wall polysaccharide biosynthetic process	4(0.56%)	12(0.04%)	1.89E-04
└ hemicellulose metabolic process	6(0.84%)	13(0.05%)	4.43E-07
└ xylan metabolic process	6(0.84%)	13(0.05%)	4.43E-07
└ glucuronoxylan metabolic process	4(0.56%)	10(0.04%)	8.38E-05
└ glucuronoxylan biosynthetic process	4(0.56%)	10(0.04%)	8.38E-05
└ xylan biosynthetic process	4(0.56%)	10(0.04%)	8.38E-05
└ xylan catabolic process	2(0.28%)	3(0.01%)	1.99E-03
└ cellular polysaccharide biosynthetic process	11(1.53%)	92(0.33%)	2.69E-05
└ polysaccharide metabolic process	18(2.51%)	156(0.57%)	1.41E-07
└ cell wall polysaccharide metabolic process	7(0.98%)	19(0.07%)	2.98E-07
└ polysaccharide biosynthetic process	11(1.53%)	98(0.36%)	4.87E-05
└ polysaccharide catabolic process	4(0.56%)	26(0.09%)	4.29E-03
└ glucan metabolic process	11(1.53%)	111(0.40%)	1.52E-04
└ cellulose metabolic process	6(0.84%)	33(0.12%)	1.83E-04
└ cellulose biosynthetic process	6(0.84%)	30(0.11%)	1.05E-04

└ cellular glucan metabolic process	11(1.53%)	108(0.39%)	1.19E-04
└ glucan biosynthetic process	7(0.98%)	64(0.23%)	1.34E-03
└ cellular polysaccharide metabolic process	16(2.23%)	138(0.50%)	6.46E-07
└ pectin metabolic process	3(0.42%)	15(0.05%)	6.29E-03
└ cellular ketone metabolic process	33(4.60%)	630(2.28%)	1.28E-04
└ carboxylic acid metabolic process	32(4.46%)	620(2.25%)	2.09E-04
└ monocarboxylic acid metabolic process	19(2.65%)	290(1.05%)	2.34E-04
└ fatty acid metabolic process	12(1.67%)	171(0.62%)	1.78E-03
└ very long-chain fatty acid metabolic process	4(0.56%)	22(0.08%)	2.28E-03
└ fatty acid biosynthetic process	9(1.26%)	105(0.38%)	1.68E-03
└ carboxylic acid biosynthetic process	18(2.51%)	307(1.11%)	1.23E-03
└ coumarin biosynthetic process	3(0.42%)	3(0.01%)	1.75E-05
└ proanthocyanidin biosynthetic process	2(0.28%)	5(0.02%)	6.40E-03
└ cellular carbohydrate biosynthetic process	14(1.95%)	173(0.63%)	1.82E-04
└ cellular component macromolecule biosynthetic process	4(0.56%)	12(0.04%)	1.89E-04
└ cellular carbohydrate metabolic process	23(3.21%)	428(1.55%)	9.17E-04
└ organic acid metabolic process	32(4.46%)	621(2.25%)	2.15E-04
└ organic acid biosynthetic process	18(2.51%)	307(1.11%)	1.23E-03
└ oxoacid metabolic process	32(4.46%)	620(2.25%)	2.09E-04
└ cellular amino acid and derivative metabolic process	46(6.42%)	483(1.75%)	4.07E-14
└ cellular amino acid metabolic process	16(2.23%)	300(1.09%)	5.59E-03
└ cellular amino acid derivative metabolic process	36(5.02%)	231(0.84%)	5.70E-18
└ cellular amino acid derivative biosynthetic process	28(3.91%)	171(0.62%)	7.80E-15
└ cellular biogenic amine metabolic process	5(0.70%)	50(0.18%)	9.44E-03
└ cellular aromatic compound metabolic process	35(4.88%)	296(1.07%)	9.21E-14
└ aromatic compound biosynthetic process	27(3.77%)	177(0.64%)	1.40E-13

			└ aromatic amino acid family metabolic process	6(0.84%)	47(0.17%)	1.31E-03
			└ xenobiotic metabolic process	2(0.28%)	4(0.01%)	3.91E-03
			└ primary metabolic process	180(25.10%)	5719(20.73%)	2.35E-03
			└ carbohydrate metabolic process	45(6.28%)	782(2.83%)	6.81E-07
			└ lipid metabolic process	29(4.04%)	578(2.09%)	6.40E-04
			└ small molecule metabolic process	74(10.32%)	1248(4.52%)	3.70E-11
KU50-Arg7	Ro ot	1(C)	└ membrane	58(21.17%)	3727(13.51%)	3.00E-04
			└ plasma membrane	30(10.95%)	1574(5.70%)	4.95E-04
			└ cell	140(51.09%)	11708(42.43%)	2.26E-03
			└ cell part	140(51.09%)	11708(42.43%)	2.26E-03
		2(F)	└ catalytic activity	107(39.05%)	7553(27.37%)	1.67E-05
			└ oxidoreductase activity	33(12.04%)	1326(4.81%)	1.26E-06
			└ oligosaccharyl transferase activity	2(0.73%)	6(0.02%)	1.44E-03
		3(P)	└ response to inorganic substance	13(4.74%)	434(1.57%)	4.40E-04
			└ response to hydrogen peroxide	5(1.82%)	41(0.15%)	5.21E-05
			└ response to reactive oxygen species	5(1.82%)	64(0.23%)	4.40E-04
			└ response to stress	49(17.88%)	1853(6.72%)	2.91E-10
			└ response to osmotic stress	13(4.74%)	388(1.41%)	1.50E-04
			└ response to salt stress	12(4.38%)	360(1.30%)	2.81E-04
			└ response to oxidative stress	12(4.38%)	247(0.90%)	7.52E-06
			└ response to heat	18(6.57%)	131(0.47%)	1.09E-15
			└ response to water deprivation	9(3.28%)	188(0.68%)	1.17E-04
			└ response to abiotic stimulus	37(13.50%)	1168(4.23%)	5.01E-10
			└ response to temperature stimulus	22(8.03%)	359(1.30%)	1.40E-11
		4(P)	└ response to stimulus	70(25.55%)	3207(11.62%)	1.20E-10
			└ response to chemical stimulus	45(16.42%)	1710(6.20%)	2.04E-09

	└ response to organic substance	22(8.03%)	1037(3.76%)	7.32E-04
	└ cellular response to organic substance	9(3.28%)	323(1.17%)	5.23E-03
	└ response to carbohydrate stimulus	8(2.92%)	177(0.64%)	4.10E-04
	└ cellular response to chemical stimulus	12(4.38%)	361(1.31%)	2.88E-04
	└ response to endogenous stimulus	18(6.57%)	835(3.03%)	1.85E-03
	└ cellular response to hormone stimulus	7(2.55%)	227(0.82%)	7.81E-03
5(P)	└ terpenoid metabolic process	5(1.82%)	91(0.33%)	2.16E-03
	└ xanthophyll biosynthetic process	1(0.36%)	6(0.02%)	5.81E-02
	└ terpenoid biosynthetic process	4(1.46%)	70(0.25%)	5.22E-03
	└ sesquiterpenoid biosynthetic process	4(1.46%)	15(0.05%)	1.19E-05
	└ abscisic acid biosynthetic process	4(1.46%)	11(0.04%)	2.97E-06
	└ apocarotenoid metabolic process	5(1.82%)	18(0.07%)	7.17E-07
	└ apocarotenoid biosynthetic process	4(1.46%)	11(0.04%)	2.97E-06
	└ abscisic acid metabolic process	5(1.82%)	18(0.07%)	7.17E-07
	└ sesquiterpenoid metabolic process	5(1.82%)	22(0.08%)	2.13E-06

[a] P=Biological Process, F=Molecular Function; C=Cellular Component b GO terms best describing all branches of the network are selected; [c] GO annotation of *A. thaliana* (TAIR10, n= 27,594) was used as the background.

Supplementary Table 18 Cyanogenic glucoside content in leaves and storage roots of KU50, Arg7 and W14

	Dry matter content	Linamarin content (μg/mg)				Lotaustralin content (μg/mg)			
		fresh		dry		fresh		dry	
		Average	stdev	average	stdev	average	stdev	average	stdev
KU50 leaf	20.54%	1.563741	0.3979986	7.6131499	1.9376759	0.2074038	0.0578004	1.0097556	0.2814039
W14 leaf	20.83%	4.2242096	0.5488795	20.279451	2.635043	0.1427003	0.0253267	0.6850709	0.1215878
Arg7 Leaf	21.17%	0.9675663	0.1881622	4.5704598	0.8888151	0.1391249	0.0380292	0.6571794	0.1796373
KU50 tuber edge	38.53%	0.2063832	0.0819677	0.5356429	0.2127372	0.0185665	0.0155602	0.0481871	0.0403845
W14 tuber edge	5.94%	0.226606	0.0535187	3.8149158	0.9009884	0.0090861	0.0031047	0.1529643	0.0522683
Arg7 tuber edge	28.46%	0.0333005	0.0226442	0.1170081	0.0795648	0.0019559	0.0015269	0.0068724	0.0053651

Supplementary Table 19 Conservation of Euphorbiaceous miRNA families across the three cassava cultivars and eight other plant species

miRNA family	AM560	W14	KU50	rco	ptc	mtr	gma	ath	vvi	osa	ppt
156	11	11	9	8	11	9	7	8	9	12	3
159	2	2	2	1	6	1	4	3	3	6	0
160	7	7	4	3	8	5	1	3	5	6	9
162	2	2	2	1	3	1	1	2	1	2	0
164	4	4	4	4	6	4	1	3	4	6	0
166	8	8	8	5	17	8	2	7	8	14	13
167	6	6	5	3	8	1	7	4	5	10	1
168	1	1	1	1	2	1	1	2	1	2	0
169	5	5	4	3	32	17	5	14	25	17	0
171	9	9	9	7	14	7	3	3	9	9	2
172	2	2	2	1	9	1	6	5	4	4	0
319	6	6	6	4	9	2	3	3	5	2	5
390	3	3	2	2	4	1	2	2	1	1	3
391	1	1	1	1	0	0	0	1	0	0	0
393	2	2	2	1	4	2	1	2	2	2	0
394	3	3	3	2	2	0	2	2	3	1	0
395	4	4	4	5	10	18	0	6	14	25	1
396	4	4	3	1	7	2	5	2	4	9	0
397	1	1	1	1	3	0	0	2	1	2	0
398	1	1	1	2	3	3	2	3	3	2	0
399	8	8	4	6	12	17	0	6	9	11	0
403	2	2	2	2	3	0	0	1	6	0	0
408	2	2	2	1	1	0	0	1	1	1	2
477	5	5	4	2	2	0	0	0	1	0	8

530	1	1	1	1	2	0	0	0	0	1	0
535	3	3	1	1	0	0	0	0	3	1	4
827	1	1	1	1	1	0	0	1	0	3	0
2111	2	2	2	1	0	19	0	2	1	0	0
2950	1	1	1	0	0	0	0	0	1	0	0
3627	0	0	0	1	0	0	0	0	1	0	0
range	1	1	1	0	0	0	0	0	0	0	0
novel-5, -6	2	2	2	1	0	0	0	0	0	0	0
novel-12	1	0	0	0	0	0	0	0	0	0	0
novel-14	1	0	0	0	0	0	0	0	0	0	0
novel-18	1	0	0	0	0	0	0	0	0	0	0
novel-19	1	0	0	0	0	0	0	0	0	0	0
novel-20	4	4	4	0	0	0	0	0	0	0	0
novel-24	1	1	1	1	0	0	0	0	0	0	0
novel-26	2	2	2	1	0	0	0	0	0	0	0
novel-30 to 32	1	1	1	1	0	0	0	0	0	0	0
novel-33	1	1	1	0	0	0	0	0	0	0	0
novel-34	0	1	0	0	0	0	0	0	0	0	0

Note: The eight other plant species are *R. communis* (rco), *P. trichocarpa* (ptc), *M. truncatula* (mtr), *G. max* (gma), *A. thaliana* (ath), *V. vinifera* (vvi), *O. sativa* (osa) and *P. patens* (ppt). The numbers listed in the table are the numbers of members in miRNA families. A "0" listed in a cell indicates that the miRNA gene is not conserved in the corresponding genome; otherwise, the number of members in the miRNA family is colored in yellow. The light yellow indicates that the miRNA family has fewer members in KU50 than the two other cassava lines, AM560 and W14. Novel miRNAs are indicated by "novel-#", e.g. novel-5.

Supplementary Table 20 Distribution of non-coding RNAs in the wild subspecies and cultivars of cassava

Summary of non-coding RNA genes in wild W14

Type	# of genes	Mean length (bp)	Total length (bp)	% of genome
miRNA	143	21		0.00
tRNA	861	74.93	64517	0.02
rRNA	337	158.19	53311	0.01
18S	115	144.08	16569	0.00
26S	178	181.77	32355	0.01
5.8S	17	101.12	1719	0.00
5S	27	98.81	2668	0.00
snoRNA	473	101.27	47900	0.01
snRNA	139	142.04	19744	0.00
SRP-RNA	31	257.35	7978	0.00
lncRNA	128782	417	53702094	12.56

Summary of non-coding RNA genes in cultivar KU50

Type	# of genes	Mean length (bp)	Total length (bp)	% of genome
miRNA	126	21		0.00
tRNA	707	74.84	52914	0.01
rRNA	192	206.03	39558	0.01
18S	59	284.22	16769	0.00
26S	110	185.38	20392	0.00
5.8S	7	113.71	796	0.00
5S	16	100.06	1601	0.00
snoRNA	364	99.94	36378	0.01
snRNA	106	139.39	14775	0.00
SRP-RNA	15	240.47	3677	0.00
lncRNA	188344	613	115454872	30.10

Summary of non-coding RNA genes in cultivar AM560

Type	# of genes	Mean length (bp)	Total length (bp)	% of genome
miRNA	146	21		0.00
tRNA	743	74.90	55650	0.01
rRNA	237	203.01	48113	0.01
18S	51	270.33	13787	0.00
26S	135	214.60	28971	0.00
5.8S	4	115.00	460	0.00
5S	47	104.15	4895	0.00
snoRNA	349	103.39	36082	0.01
snRNA	89	146.85	13070	0.00
SRP-RNA	30	247.93	7438	0.00

Supplementary Table 21 Comparison of predicted protein sequences of genes involved in photosynthesis and sucrose and starch synthesis between cultivar KU50 and wild W14 relative to cultivar AM560

Cassava varieties	Light reaction	Calvin cycle	Synthesis of sucrose and starch
	Similarity (%)	Similarity (%)	Similarity (%)
KU50	98.7±5.37	91.9±16.44	90.1±10.94
W14	86.5±18.76	74.3±22.38	79.8±20.56

3) Supplementary Notes

Supplementary Note 1 Choice of cassava ancestor and cultivated variety for WGS

Manihot genus includes about 98 species occurring in both northern South America (80) and in Mexico/Central America (17). Cultivated cassava is derived from a single wild South American progenitor (referred to as *M. esculenta* ssp. *flabellifolia*) occurring in northern Mato Grosso, Rondônia and Acre states in Brazil and adjacent areas of northern Bolivia[1,3-4]. Cassava genomes are extremely heterozygous due to its fertilization by open pollination as part of its natural evolution and vegetative propagation habits selected during 7000-12000 years of domestication[5-7]. In this study, we used two cassava accessions, W14 and KU50, for whole genome sequencing. W14, kindly provided by the Germplasm Unit of CIAT, is an accession of the wild cassava subspecies, *M. esculenta* ssp. *flabellifolia*, the nearest ancestor of cultivated cassava. It was originally collected in Brazil[2,4]. It is an ancestor of cultivated cassava, a middle type between wild ancestor species, *M. esculenta* ssp. *flabellifolia*, and modern cultivated species. KU50 is a representative cultivar of the cultivated cassava, *M. esculenta*. It was bred by Kasas University in Thailand and has been extensively used in commercial plantations in East Asia. There are obvious differences in agronomic and economic traits between the two genotypes (**Supplementary Table 1)**. KU50 is propagated by stem cuttings and seldom bears fruits, but it has a high tuber root yield, with3 to 10 kg/plant per year. KU50 has a high photosynthesis rate and its starch content in fresh tuber roots ranges from 28 to 32%. On the other hand, W14 usually produces a large number of fruits and is propagated only by seeds. However, the photosynthesis rate of W14 is lower than that of KU50. Its tuber root yield is only 0.5 - 2.0 kg/plant per year and its starch content in fresh tuber roots is only 3 - 5% (**Supplementary Figure 1)**. Two other cultivated varieties, CAS36 and Arg7 were used in the experiment. CAS36, a self pollinated generation (S1.600) of sugary cassava, a natural mutant supplied by

EMBRAPA Brazil with high sugar content (15-20%), substantial starch content (5%) and large tuber roots, was used for production of 20-fold coverage re-sequencing of genome. Arg7, a variety with elite agronomic traits bred in Argentina was used for transcription profiling and annotation with KU50 and W14.

Supplementary Note 2 Construction of BAC library and physical map

Construction of BAC libraries A cassava inbred line, AM560-2, was used for BAC library construction. DNA was partially digested with *Hin*d III, cloned into the vector pIndigoBAC536 and transformed into the *E. coli* host DH10B. A total of 72,192 clones were obtained, with an average insert size of 115 kb and approximately 7% of the clones having no insert. The library represents about 11x coverage of the cassava genome, based on its genomic size of 740 Mb. The accession W14, a wild ancestor of cultivated cassava, was also used for BAC library construction. The BAC libraries were constructed by Amplicon Express Inc. (Washington, USA) using the method of Tao *et al.*[8]. DNA was partially digested with *Eco*R I and *Hin*d III, respectively, and cloned into the pCC1BAC vector. The W14 BAC libraries consist of a total of 59,904 clones, with an average insert size of 115 kb for the *Eco*R I library and 129 kb for the *Hin*d III library, representing approximately 10 genome equivalents of the genotype (**Supplementary Table 1**).

BAC Fingerprinting A total of 72,192 BAC clones from the cassava AM560 library and 29,952 BAC clones from the W14 libraries were fingerprinted using the SNaPshot-based high-information-content fingerprint (HICF) technique[9-10], with modifications[11]. 0.5 - 1.2 μg of BAC DNA was simultaneously digested with 2.0 units each *Bam*HI, *Eco*RI, *Xba*I, *Xho*I and *Hae*III (New England Biolabs, Beverly, Massachusetts) at 37°C for 3 h. The DNA was labeled with 0.4 μl of the SNaPshot kit (Applied Biosystems, Foster City, California) at 65 ^{o}C for 1 h and precipitated

with ethanol. The labeled DNA was dissolved in 9.9 µl of Hi-Di formamide, and 0.3 µl of GeneScan 1200 LIZ (Applied Biosystems, Foster City, California) was added to each sample as an internal size standard. Restriction fragments were sized with ABI3730XL using 50-cm capillaries and POP7 (Applied Biosystems, Foster City, California). The fragment size calling was accomplished with the GeneMaper software (Applied Biosystems, Foster City, California) with assistance of FP Pipeliner (http://www.bioinforsoft.com/). Two BAC clones were inserted in each plate as a quality standard and to detect incorrect plate orientation. Clone fingerprints were edited with combination of FPMiner software (BioinforSoft, Beaverton, OR) and GenoProfiler[12] using the following criteria: Fragments in the size range of 75 – 1,000 bp were measured. For the data quality control, vector bands and clones failed in fingerprinting or lacking inserts were removed. In addition, samples with fewer than 25 or more than 200 fragments were excluded from the analysis. Fingerprints of cross-contaminated samples were detected using a module in the FPMiner and removed from the data set. The cross-contamination was defined as clones residing in neighboring wells in either 384-well format or 96-well format (quadrants) plates and sharing 30% or more of the mean numbers of fragments calculated using the formula: shared bands*2/(bands of clone 1 + bands of clone 2).

Contig assembly For the AM560 physical map assembly, after fingerprint editing, 58,244 clone fingerprints representing 8 cassava genome equivalents, were suitable for contig assembly. These clone fingerprints were then used for an initial automated contig assembly using the FPC software[13], with a tolerance of 5 (0.5 bp). The initial assembly was performed at a relatively high stringency (1×10^{-45}) to minimize contig assembly of clones from unrelated regions of the genome. The “DQer” function was used to dissemble contigs containing more than 15 % questionable (Q) clones. The “Singleton to End” and “End to End” functions were employed to merge contigs that are actually overlapped by successively decreasing the assembly stringency, i.e., of increasing the Sulston cutoff values. At last, the 10% largest contigs were subjected to manual editing such as examining and disjoining the contigs with CB map analysis. At the end, the FPC assembly resulted in a total of 2,105 contigs and 5,054 singletons

(**Supplementary Table 2**). For the W14 physical map assembly, similar assembly procedure and parameter settings were used, resulting in a total of 2,485 contigs and 2,909 singletons (**Supplementary Table 2**).

BAC end-sequencing The minimum tilling path (MTP) clones were picked from both the AM560 and W14 assemblies and end sequenced. The BAC end sequences (BESs) were generated using the Sanger sequencing approach (ABI 3730XL) and used to align whole-genome shotgun sequence contigs and scaffolds onto the physical map BAC contigs.

Supplementary Note 3 Genome size estimation

As shown in **Supplementary Figure 2**, the main graph depicts the distribution of 17mer, 21mer, 25mer, and 29mer in the reads of short insert size libraries (200-500 bp) and the inset shows the volume of 25mer corrected by the kmer spectrum method. The peak at low frequency and high volume represents random base errors and heterozygosity in the raw sequences. The high frequency and volume peaks at 63 may be due to the presence of Endophyte sequences. The total kmer number of ‘k=25 corrected’ is 9,644,794,319, and the volume peak is 13, so the genome size can be estimated in 742 Mb using the formula: (total kmer number)/(the volume peak[1]).

Supplementary Note 4 Data description

The *Manihot esculenta* genome contains 18 chromosome pairs. We used a combined whole-genome shotgun sequencing (WGS) and BAC pooling (BP) strategy. Sequence data was produced on Illumina Genome Analyser II, HiSeq 2000 and Roche 454 GS FLX at the Beijing Institute of Genomics (BIG) and Qingdao Bioenergy and Process Institute of Chinese Academy of Sciences (CAS). Hierarchical insert library construction was followed by Illumina paired-end sequencing protocol. The libraries' insert sizes were generally 200-300 bp, ~500 bp, 1-2kb, ~4kb, and 10-20kb, in addition to a long-insert (10-20kb) pair-end 454 library for each genome (**Supplementary Figure 4**). For BAC pooling (BP) data, a set of 9984 genome BAC clones (26×384) of W14 was sequenced with 300-500bp pair end by Illumia Highseq 2000, it covered 42.12 fold of genome according to its average insertion of 125kb per clone. Totally, genome sequence coverage of 103x and 46x was obtained for W14 and KU50, respectively (**Supplementary Table 1; Supplementary Table 3**). We also generated a high-quality re-sequencing data set for sugary cassava CAS36, with a genome coverage of 21x (**Supplementary Table 4**).

Supplementary Note 5 Assembly strategy

Cassava draft genome sequences were assembled with multi-formed sequencing data using the following strategies, the following of which programs were parts of GNU software package and GATE v1.0 (https://github.com/BENMFeng/GATE).

Data pre-processing Low-quality PCR duplications were discarded using the PERL script '*filterPCRdup.pl*'; duplicated reads were identified by seed generated from 75% bases of 5' leftmost of paired-end reads, and only the highest quality copy was retained. Then, we used the C++ program '*scanAP*' (http://code.google.com/p/biowiki/) to find out the reads that had sequencing adapter or artificial nucleotides based on pairwise local alignment, and trimmed them by PERL script '*trim_seq.pl*' according to the alignment location. Lower-quality (Phred quality <Q13, i.e. $Q=-10\log_{10}P_{error}$, $P_{error}<=5\%$), fluctuating bases quality distribution (s.d.>10) or with ambiguous calling bases (Ns) over 10% of reads should be discarded, and low quality (Q<13) or with 'Ns' bases that had not been called as any kind of nucleotides at read termini would be trimmed as not shorter than 25 base pairs, whereas it won't be preserved. These processes were carried out by PERL script '*fastqcut.pl*'.

Pollution removal Using BWA[21] v0.6.1 with parameters set as 'aln -l 31 –k 0' to align all reads to Bacterium or potential pollution during the whole library construction and sequencing processes, and discarded the reads that perfectly matched to the pollution sequences. We separated the nuclear and organelle genome sequence by alignment to pre-assembly of chloroplast and mitochondrial sequence, for the reason that an organelle genome has 1000-fold more copies than a nuclear genome in plant DNA library. Therefore, we could reduce the artificially induced complexity of the sequences for draft genome assembly.

Error corrections According to the kmer-frequency distribution compared to kmer species based on Lander-Waterman model with Fan's algorithm[14], we eliminated or corrected the reads with low kmer frequency (kmer from 17 to 25) using a C++ program of '*ec*' –"Error Correction" in some reads, but the same kmer sets in others

with more higher (>2 times) frequency, of which most were due to random base calling errors.

De novo assembly Illumina sequences were assembled using SOAPdenovo v1.05[15], and optimized the assembly quality via kmer parameters set from 17 to 41 for different data sets with read lengths ranging from 50 to 101 bp, according to kmer frequency distribution and optimizing kmer estimation (**Supplementary Note 3**).The major assembly process followed the flow: Contigs construction -> scaffolding -> GapCloser (v1.12-r6, http://soap.genomics.org.cn/about.html#resource2).

Hybrid assembly Whole genome shotgun (hereinafter referred to as the unified abbreviation 'WGS') illumina short reads (50~101bp, **Supplementary Note 4**), BAC pooling (hereinafter referred to as the unified abbreviation 'BP') shotgun illumina short reads (75~101bp, **Supplementary Note 2 and 4**), and 454 'long' reads (250~550bp), respectively we had designed different strategies and programs to assemble, and used a hybrid assembly strategy for combined them to be united. For short illumina short reads we using SOAPdenovo v1.05 based on de Bruijn Graph algorithm as coalescent description, respectively for WGS and BP sequences. Using MSR-CA v1.6.1 (http://www.genome.umd.edu/SR_CA_MANUAL.htm) we clustered the illumina short reads (50~101bp) that with at least 35bp mapping to 454 prevenient assembled sequences into super reads (200bp~500bp), then took these super reads and 454 reads as input for Newbler v2.5.3[16] to construct the third set of contigs, for the reason of we just have low coverage depth of 454 sequencing reads (**Supplementary Note 4**). Consequentially, using BLAST v2.2.25 with parameters of e-value set as 1e-10, the contigs that were totally part of sequence of the other contigs, the smaller fragments or duplicated copies that were treated as redundancy, had been removed from the combined contigs. Then we used Phusion[17] and CAP3[18] to merged contigs based on greedy graph and OLC algorithm.

Insert size distribution, classification and GC content Using BWA[19] v0.6.1, we aligned all the paired-end (300, 400, 500bp insert size libraries) and mate-pair (1.0k, 4.5k, 8 -10k, 12 - 20k insert size libraries) sequences to the W14 draft genome,

extracted the mapping insert size span by filtering the unmapped reads with SAMtools v0.1.18[20] and fixed the mate-pair information by Picard-tool-kit v1.51 (http://picard.sf.net) from the alignment BAM file, while the PE/MP had proper pairs flag '0x2'. So, we separated and grouped the libraries to be 300bp, 400bp, 500bp, 1kb, 2kb, 4kb, 8kb, 10kb, 12kb, 15kb and 20kb. The distribution of the insert sizes of the paired-ends and mate-pair libraries are shown in **Supplementary Figure 4.** After eliminated the polluted sequences of other known species of GeneBank using BLAST, GC content was used for estimation of assembled contigs sourced from the same segment. As shown in **Supplementary Figure 5**, the GC content distribution of W14 and KU50 were similar, indicating that the GC content of cassava should be around 34% and 36%.

Scaffolding According to pairwise local alignment which did not allow gaps, but maximize two mismatches in 25 tuples seed, paired-ends, mate-pairs of Illumina reads and long pair ends of 454 reads were mapped to the merged contigs. Contigs were in conjunction with other contigs as scaffolds linked by 1kb, 2kb, 4kb, 8kb, 10kb, 12kb, and 20kb insert size libraries mapping information, using C++ program link_scaffold_v0.4[16].

Assembly error estimation and correction We aligned the high-quality reads to interval assembly contigs to self-genome using BWA[19] 0.6.1. We detected the SNVs and InDel of the self-genome using GATK v1.1-30-g2b2a4e0[20-21]. Using the 'BaseRecalibrator' and 'IndelRealigner' functions of GATK v1.1-30-g2b2a4e0 and the 'fillmd' function of SAMtools v0.1.18 to generate MD tag, we recalibrated the mapping quality and realigned to be more accurate due to InDel localization. Most homozygous SNVs and InDels in self-alignment were caused by assembly errors and low sequence coverage, so, we corrected these loci, according to genotyping consensus by the 'mpileup' function of SAMtools v0.1.18, which is computed by the maximum likelihood of genotype. Meanwhile, we corrected the heterozygous loci by selecting the maximum likelihood of allele base with the highest sequencing quality (Phred quality) ≥20 and mapping quality ≥60 (-10 log10 Pr{fmapping position is wrong}).

Supplementary Note 6 Evaluation of the draft assembly of W14 by BAC sequences

Five randomly selected BACs were sequenced using Roche 454 sequencing methods, and were assembled into 24 contigs using Newbler v2.6.3[16]. To investigate the accuracy and completeness of the full genome assembly, we aligned the 24 contigs of the 5 BACs to the W14 draft genome, using BLAT with 90% or more identity. Comparison of the assembled scaffolds of W14 to the fully sequenced BACs (**Supplementary Table 5**) revealed that draft genome scaffolds spanned approximately 62.4% of the BACs. This ratio is approximately equivalent to the assembled draft genome of 432 Mb which represented for 58.2% of the whole genome size. In the aligned regions, the rate of single base differences (the mismatch ratio between them) was about 6.1 bases per kilo-base (kb). The matched and mismatched bases were counted by the results of full-length alignment using BLAT[22] (**Supplementary Figure 6**).

Supplementary Note 7 Integrated scaffolding of physical map and draft genome

To anchor the draft genome to physical map[23] and generate mega-scaffolds, we sequenced 8,361 BAC ends (BES), which were sourced from the BAC clones used to construct the Finger Printed Contigs (FPC[24-26], generated by Luo[9]). We aligned these BESs to hard masker genome (ReatpeatMasker, see Note 12), identified the mappable mate pair relationships with contigs, and then linked the contigs to the FPC according to estimated direction and rank by BAC clones' ordering on FPC. Wefiltered the confused rank and repeat anchored contigs, scaffolded and added a certain length of 'Ns', given by the size of BAC clone and FPC distance information, to join the contigs anchored with the same FPC CTG. These processes were operated

by the *wfbscaffolds.pl* pipeline (of GATE), following the flowchart as **Supplementary Figure 7**. The final mega-scaffold result is shown in **Supplementary Table 6**.

Supplementary Note 8 Draft genome statistics

The draft genome sequence of W14 consisted of 33,166 scaffolds spanning 426 Mb, with an N50 size of 33 kb an N80 size of 26 kb covering 3,716 scaffolds and the largest scaffold size of 277 kb. A total of 34,483 gene models were *ab inito* predicted from the W14 draft genome, with a gene density of 10.37% and an average exon size of 190 bp (**Supplementary Table 6**).

The draft KU50 genome assembly consisted of 62,073 scaffolds spanning 385 Mb, with an N50 size of 13 kb, an N80 size of 2 kb covering 11,782 scaffolds and the largest scaffold szie of178 kb. A total of 38,845 gene models were *ab inito* predicted from the KU50 draft genome, with a gene density of 13.46% and an average exon length of 184 bp (**Supplementary Table 6**).

Supplementary Note 9 Gene Prediction

We utilized five *ab initio* predictors to construct the entire predicted genes structure *in silico*For W14 and KU50, respectively,39,919 and 43,318 genes were predicted using AUGUSTUS[27], 64,236 and 65,960 genes predicted using SNAP[28], 58,348 and 83,484 genes predicted using GeneMark-ES[29], 44,978 and 53,028 genes predicted using GENSCAN[30], 33,038 and 59,763 genes predicted using GENEID.

Moreover, we predicted gene structures in the W14 and KU50 genomes using a similarity-based approach with GenomeThreader (GTH[31]) via spliced alignment. Euphorbiaceous proteins and *M. esculenta* ESTs and cDNA sequences were used in the analysis. As a result, 19,982 and 23,091 genes were predicted for W14 and KU50, respectively. Furthermore, according to PASA[32] based on collapsing alignments to transcripts on the basis of splicing compatibility, we aligned ESTs and full-length cDNAs to the genomes via GMAP[33], and assembled RNA-seq sequences utilizing Inchworm, a component of Trinity[34] in PASA[35] prediction process. 21,083 and 25,185 unique genes were manually reconstructed for W14 and KU50, respectively.

Genome-guided RNA-seq transcriptome reconstruction was followed by Tophat-Cufflinks protocol. Finally, 34,483 and 38,845 genes were annotated for the W14 and KU50 genomes. The gene predictions generated using different approaches were combined with spliced alignments of proteins and transcripts into a weighted consensus gene structure using the evidence-based combiner - EVidenceModeler (EVM[35]) via a weighting: (ab initio predictions) ≤ (EST alignments) < (GenomeThreader) < (RNA-seq).

In addition, we used GeneGffMasker.pl of GATE (URLs) to identify the UTRs, start codon and stop codon according to the predicted ORF via HMM algorithm and revised the Gff v3.0 annotation result. The predicted gene statistical report is shown in **Supplementary Table 6** and the CDS length distribution of the two cassava genomes is shown in **Supplementary Figure 8**.

We evaluated the gene region coverage of the draft genomes of W14 and KU50 with 201,392 ESTs and transcripts that resulted from 12 RNA-seq libraries of W14, KU50

and Arg7. As the cumulative frequency distribution, the ESTs and transcripts with identity values of over 94.9% and 92.8% were mapped to the draft genomes of W14 and KU50, respectively (**Supplementary Figure 9a, b**). Then, we validated the *de novo* predicted gene models with annotated transcriptome (**Supplementary Figure 9c**). Compared to over 90% mapped reads, approximately 55.3 – 66.3% *de novo* assembled transcripts could be aligned to predicted genes in W14 and KU50. However, 75 – 87.9% annotated transcripts with gene products could be aligned to predicted genes in W14 and KU50 genome. The unmappable transcripts were probably the non-protein coding RNA, most of which were sourced from lncRNAs. We had independently investigated the IncRNAs in **Supplementary Note 21**. The unmappable annotated transcripts could be lost in un-annotated assembly or lacked in the assembly. These results indicated that the most of the gene structures were covered in the assembly and gene sets predicted from the draft genomes of W14 and KU50. Their functional annotation is shown in **Supplementary Table 7**.

Supplementary Note 10 Functional annotation in comparative genomes

Gene annotation was carried out using BLAST v2.2.25[36] with the *blastn* parameter as 1e-5 to search the 'best hit' of nucleotide sequences of genes from NT (release 02-Dec-2011, URLs) database, and with the *blastp* parameter as 1e-5 to search the 'best hit' of peptide sequences of genes from KEGG[37-38] (Release 05-Sup-2011), NR (release 02-Dec-2011, URLs), TremBL[39] (Release 08-Feb-2011), SwissProt[39] (Release 08-Feb-2011), COG[40] (Release 05-Sup-2011,), and Pfam[41] (Pfam27.0, 21-Dec-2012) databases using InterproScan[42] to identify additional GO annotation for wild and cultivated cassava genes. Consequently, approximately 97% of the W14 and KU50 genes were annotated to known genes; only 3.4% and 3.2% of the genes

were unknown or novel genes. The statistical result of annotation is shown in **Supplementary Table 7**. The gene ontology enrichment analysis of all functional genes with WEBGO[43] showed that there was no clear difference of gene categories between W14 and KU50 (**Supplementary Figure 10**).

Supplementary Note 11 Genome heterozygosity in cassava

To compare the diversity of the three cassava genomes, W14, KU50 and AM560-2, we aligned the raw reads to their draft genomes using BWA v0.6.1[19], and called SNVs (single nucleotide variants) using GATK[20-21] v1.1-30-g2b2a4e0. The data used for the analysis included 40-fold paired-end reads of W14 and 24-fold paired-end reads of KU50 that we sequenced using Illumina GAII and the 454 reads of AM560-2 downloaded from NCBI SRA accession SRS193279 released by the DOE JGI team. We used the draft genome sequence of AM560-2 released version of Mesculenta_assembly_147 (cassava4.1) as the cassava cultivar genome of AM560-2's reference. We obtained 1.37 million of SNVs for W14, 0.81 million of SNVs for KU50, and 0.51 million of SNVs for AM560. The SNV density values were 3.89, 3.50 and 1.44 SNVs per 1000 bp for the genomes of W14, KU50 and AM560, respectively. Because AM560 is an inbred cultivar, it only has an approximately half SNV rate as the other cultivar sequenced, KU50. It was apparent that the wild cassava W14 has a higher heterozygosity than either of the cultivars. According to Per SNV heterozygosity and kmer frequency distribution (see **Supplementary Note 3**), the species *M. esculenta* has an extremely high heterozygous rate, which brought in the major complexity of *de novo* assembly of the cassava genomes. The more details of heterozygosity of the three genomes are shown in **Supplementary Table 9**.

Supplementary Note 12 Genome diversity with SNVs and InDels

W14, KU50 andCAS36 reads generated with Illumina GAII were mapped to the AM560 v4 draft genome assembly (Phytome v7.0, URLs) created by JGI using BWA[19] (v0.6.1) – SAMtools (v0.7.1) – Picard (v1.51) – GATK[20-21] (v1.1-30-g2b2a4e0) – Dindel[44] (v1.01), which executed the pipeline on Grid Linux cluster by a batch pipeline shell generator - GATE (URLs). A total of 4.8 million of SNV loci were detected, when the W14 reads were aligned to AM560, and approximately 3.6 and 3 million of SNVs were detected, when the KU50 and CAS36 reads were aligned to AM560, respectively. Meanwhile, we found 0.4 million of InDels with W14, 0.3 million with KU50, and 0.2 million with CAS36, when they were aligned to AM560. Both the SNV and InDel search results showed that wild cassava (W14) is more diverged than cultivated cassava (KU50, CAS36) from AM560. And there are more shared diversity loci between KU50 and CAS36 than with W14 (**Supplementary Tables 10 and 11; Supplementary Figure 12**).

Supplementary Note 13 Comparing genomes in Euphorbiaceae and estimation of cassava divergence time

Gene function annotation The motifs and domains of genes were determined by InterProScan against protein databases including Pfam, PRINTS, ProFile, SuperFamily, ProDom and SMART. Gene Ontology (GO) IDs for each gene were obtained from the corresponding InterPro entry.

Gene family (OrthoMCL) BlastP was used on all the protein sequences against a database containing a protein dataset of *M. esculenta*, *Jatropha curcas* (Barbodos Nut), *Ricinus communis* (castor bean), *Arabidopsis* and *Vitis vinifera* (grape) under an E-value of 1E-5.The OrthoMCL method with mode 3 was applied to construct gene families.

Gene family (Blast) BlastP was used on all the protein sequences against a database containing a protein dataset of all the five species (*M esculenta*, *J. curcas*, *R. communis*, *Arabidopsis* and grape) under an E-value of 1E-5. Single-linkage group method was used to construct gene families. If gene A, B and C are from three species respectively, A is a blast hit of B and B is a blast hit of C, then we think that A, B and C form a gene family.

Comparative gene family The comparison of gene families among the threespecies in Euphorbiaceae and grape is shown in **Supplementary Figure 13**. There were 2,043 unique gene families in *M. esculenta*, being much higher than those in *J. curcas* (532) and *R. communis* (826). Gene Ontology (GO) enrichment analysis revealed that there was a significant difference in categories of viron, viron part, viral reproduction, protein tag, locomotion and cell killing among the three species in Euphorbiaceae (**Supplementary Figure 14**).

Comparison of gene models in Euphorbiaceae Comparative analyses were done among cassava, castor bean, Barbados nut and 12 other plant species (for the 12 plant species, see below) to find cassava species-specific genes. For cassava, we used the three cassava predicted CDS sequences (wild W14, cultivars KU50 and AM560). The Barbodosnut CDS sequences were developed by South China Botanical Garden,

Chinese Academy of Sciences. The castor bean and 12 other plant species (*Populus trichocarpa*, *Gossypium raimondii*, *Cucumis sativus*, *Medicago truncatula*, *Aquilegia coerulea*, *Arabidopsis thaliana*, *Prunus persica*, *Citrus sinensis*, *Solanum lycopersicum*, *Oryza sativa*, *Zea mays* and *Sorghum bicolor*) CDS sequences were downloaded from Phytozome. We searched the three cassava CDS sequences against those of castor bean, Barbadosnut and 12 other plant species by BLAST. The three cassava CDS sequences were combined using previously defined gene models. The BLAST results were filtered using an e-value of < 1e-10 and a bit score of > 200. The details of the cassava gene models matched against other species are shown in **Supplementary Figure 15**. We found that 8,414 out of 34,153 (24.6%) cassava gene models were species-specific, while this number might be magnified by gene fragmentation. We also found 3,710 Euphorbiaceae-specific genes, which were shared among the Euphorbiaceae species, but not in 12 other plant species.

Estimation of divergence time of cassava Comparison with gene set in nuclear genome of a species, chloroplast sequences are more conservative and with more overlap sequences for phylogenetic analysis of it. We used 71 chloroplast genes from eight species and cassava with wild subspecies W14 as well as KU50 and AM560 in cultivated subspecies for estimation of divergence time of cultivated cassava from its wild ancestor W14. These eight outer species are:

Family	Species	Abbreviation
Fabaceae	*Medicago truncatula*	Mtr
Cucurbitaceae	*Cucumis sativus*	Csa
Salicaceae	*Populus trichocarpa*	Ptr
	Populus nigra	Pni
	Populus trichocarpa x *Populus deltoides*	Ptd
Euphorbiaceae	*Ricinus communis*	Rco
	Euphorbia esula	Ees
	Jatropha curcas	Jcu

Manihot esculenta ssp. *flabellifolia*	(W14)	Mef-W14
M. esculenta ssp. *esculenta* (cultivar KU50)		Mes-KU50
M. esculenta ssp. *esculenta* (cultivar AM560)		Mes-AM560

Bayesian divergence time was estimated based on the concatenated dataset of the 71 amino acid sequence alignment using BEAST[45] v1.7.5. To reduce the negative effects of heterogeneity of substitution rates, a relaxed molecular clock model of uncorrelated log normal distribution (UCLD) was selected. JTT+G model with four Gamma Categories and a Yule process for tree prior were specified. For the time calibration, two time constraints in previous study[46] were used. The most recent common ancestor (MRCA) of *Fabales* and *Cucurbitales* was assumed to be a normal distribution centered at 98 million years ago (MYA) with a standard deviation of 2.5 MYA. The MRCA of *Salicaceae* and Euphorbiaceae was treated as a normal distribution prior as well, with the standard deviation of 1 MYA and the mean set to 89 MYA. Markov Chain Monte Carlo (MCMC) chain length was set to 1, 000 million with sampling at every 100,000 generations, resulting in 10,000 trees. Tracer v1.5 (http://tree.bio.ed.ac.uk/software/tracer/) was used to assess the convergence by effective sampling size (ESS) values of all parameters greater than 200. A maximum clade credibility tree was generated using TreeAnnotator v1.6.1 with the first 30% of the trees excluded as burn-in. Finally, the divergence times were visualized in Figtree v1.3.1 (http://tree.bio.ed.ac.uk/software/figtree/). The estimation results are shown in **Fig. 1d**.

Supplementary Note 14 Comparison of the draft genomes between W14 and cultivated cassava

We compared the predicted ORFs of the three cassava genomes (W14, KU50, AM560) with the *A. thaliana* and *R. communis* trascriptomes. We grouped the *A. thaliana* and *R. communis* genes as reference clusters according to the sequence homology. A total 9,886 gene clusters with an identity value (match length/gene length) of over 50% were grouped using the best hit of global alignment tool Blat[24] and a hieratical clustering. Then, we used a pairwise alignment strategy to compare cassava ORFs to the clusters, compare the three cassava ORF sets to each other and compare all the ORFs to themselves. We listed each ORF perfect match of *A. thaliana* and *R. communis* gene clusters, the orthologous ORFs, and the perfect matches to the other ORF of itself genome. Finally, we grouped them together by the relationships of gene ID index. The perfect match we donated as the identity value required the identity value in accordance with the condition of：

$$\begin{cases} match \geq 100 \\ \dfrac{match}{min} \geq 0.5 \end{cases} \quad if\ \ min \geq 100$$

$$\begin{cases} match \geq 50 \\ \dfrac{match}{min} \geq 0.8 \end{cases} \quad if\ min < 100$$

where ***min*** is denoted as the minimal length as the length of two pairwise alignment ORFs; ***match*** is denoted as matched homology length of two pairwise alignment ORFs. In total, 34,154 gene CNVs were composed of 28,072 genes for W14, 31,310 genes for KU50, and 28,484 genes for AM560. Using an 80% similarity pairwise alignment, 6,567 genes were identified in single copy in the three genomes.

For PAV (present and absent variation) analysis, the PAV genes among the W14, KU50 and AM560 genomes were grasped directly. Considering *de novo* assembly and *ab inito* gene prediction false negatives, we filtered the PAV genes which still could be found in the draft genome with 100% coverage or 30% coverage and over 30x coverage in raw read mapping. The results showed that 1,584 genes were only present in W14 but absent in cultivars, while 1,678 genes were only present in

cultivars but absent in their wild progenitor W14. GO annotation revealed that most of the PAV genes were ascribed into six biological processes, including binding, catalytic activity, metabolic process, cellular process, cell and cell part (**Supplementary Figure 17**), suggesting that these genes have been strictly selected during the process of long-time domestication. Copy Number Variation (CNV) annotation found that of the genes with significant difference in CNV between W14 and cultivars, 30 genes have high CN only in W14 and 80 genes have higher CN only in cultivars. All these genes are involved in catalytic activity, binding and transferase activity (**Supplementary Figure 18**).

Supplementary Note 15 Comparison of SNV/InDel and SV in one-to-one single copy genes

A total of 6,567 one-to-one single-copy genes among the three cassava genomes, W14, KU50 and AM560, have been used for evaluation of gene structural variation during the process of domestication. Structure Variations (SV > 50 bp) between W14 and cultivars appeared in 1,830 genes, with a ratio of 27.86%. Of these genes, 881 had an average of 1.59 SVs within the body of a gene and 1,108had an average of 1.67 SVs in the 2,500-pb region of 5’ upstream of a gene. Only 146 genes had SVs in both gene body and 5’ upstream region. Further statistical analysis showed that most of the SVs are insertions (610), with 583 being in introns, 18 in exons and 9 spanning over introns and exons, and deletions (797), with 685 being in introns, 40 in exons and 72 striding over introns and exons. There were only three inversions among the 881 genes that had SVs in gene body (**Supplementary Table 12**). GO analysis could not find the selective trend of the genes (**Supplementary Figure 19**). As one type of DNA transposons, miniature inverted-repeat transposable elements (MITEs) have been proven to be highly associated with gene expression. We searched the 1-kb

upstream regions of the orthologous genes in W14 and two cultivars. 553 MITEs were found, of which 310 and 243 were present in the genes of AM560 and W14, independently (**Supplementary Data 6**), and 143 MITEs in AM560 were absent in cultivar KU50.

SVs that took place in all 6,567 single-copy genes were figured out by paired-end mapping (PEM) and their depth coverage generated via pairwise alignment to estimate the breakpoints of SV. One of examples of deletions and inversions is shown in **Supplementary Figure 20**, showing that a fragment with a length of 1,913bp in the introns of KU50 and AM560 was deleted in the corresponding genomic region of W14. So, when we used PEM for breakpoint diagnosis, we could see the normal (with forward-reverse so called FR strand of both ends of pairs and spanning in the size of library construction) paired-ends that were aligned to W14 were abnormally (without FR strand of both ends of pairs, or the spans of alignment insert size were extended or shrunk) extended from 300 bp to 2,000 bp, which exactly crossed over the fragment with a length of 1,913 bp in KU50 and AM560 and the coverage depth of which was lower than the other regions, except for the small repeat peaks.

SNV/InDel analysis found that 70 genes with no SNV/InDel and 891 genes with SNV/ InDel variations were only in the W14 genome, including 277 genes with less than 1.5% mutation frequency. It was also shown that there was a lower frequency of both SNVs and InDels in gene body than the upstream and downstream of the genes between wild species and cultivars. GO annotation revealed that the 70 genes without SNV/InDel were ascribed into four biological processes, binding, catalytic activity, metabolism process and cellular process in the importance of descending order (**Supplementary Figure 21**). The other two clusters of genes with SNV/InDel only present in the W14 genome were mainly assigned into the four biological processes and six other biological processes (**Supplementary Figure 22 and 23**). These results indicated that these genes have been sweepingly selected by nature and humankind.

Supplementary Note 16 Selection pressure driving genome variation from wild to cultivated cassava

We identified 16,219 orthologous groups using the pairwise similarity scores calculated with Blast among three complete proteomes. The synonymous (*Ks*) and nonsynonymous (*Ka*) divergence values and selective pressure (*Ka/Ks*) among the genomes of KU50, AM560 and W14 were calculated with 16,219 high-confidence 1:1:1 orthologous genes (**Supplementary Table 13**). The *Ka*, *Ks* and SNVs (*Ka+Ks*) in gene body generally were higher from cultivar to wild ancestor than that between cultivars. We figured out the distribution of SNVs with KU50 vs. W14 and KU50 vs. AM560, showing a systemic difference from wild subspecies to cultivars (**Supplementary Figure 24**). But the general selective pressures (*Ka/Ks*) had no considerable difference among the three genomes that could result from **Supplementary Figure 25.** Therefore, we focused on those genes that have low selective pressure (*Ka+Ks*=0, *Ka/Ks* log2 < -5) between cultivars KU50 and AM560 (**Suppplementary Table 15**). We identified 4,982 (37.8%) genes with the criteria and used them to re-calculate the *Ks*, *Ka* and *Ka/Ks*. Consequently, we found that there were extreme differences in *Ka*, *Ks* and *Ka/Ks* between W14 and KU50, and between W14 and AM560, relative to that between KU50 and AM560. This result suggested that this set of genes have been severely selected from wild ancestor to cultivar, thus leading to a reduced degree of diversity (**Supplementary Table 15**).

Those genes with characteristics of positively (Ka/Ks>1) and negatively (Ka/Ks<1) selection between wild w14 to cultivated cassava were used for Bingo (biological networks gene ontology) analysis. The Bingo is an open-source Java tool to determine which Gene Ontology (GO) terms are significantly overrepresented in a set of genes. Bingo can be used either on a list of genes, pasted as text, or interactively on sub graphs of biological networks visualized in Cytoscape (version 2.6.2, http://www.cytoscape.org/). P-genes obtained after filtering based on fold change cut off (Wild-Cultivar Ka/Ks>1) in stage 1 were taken as the input list for pathway analysis. All the results were shown in **Supplementary Figure 27**.

Supplementary Note 17 Comparative transcriptomes from wild to cultivated cassava

RNA-seq data sets Twelve RNA libraries of developing leaves, stems and storage roots sampled from plants of W14, KU50 and Arg7 were sequenced by Illumina HiSeq 2000. From 9.7 M to 68.6 M reads with approximate 100 bp in length were acquired for each library, being equivalent to 1.3 to 9.2x coverage of the cassava whole genome (742 Mb).

Assembly and annotation After pre-processing, the mRNA sequence reads of the 12 samples were mapped to the AM560-2 reference genome sequences using Bowtie[47] v0.12.7, TopHat[48-49] v2.0.0, and Cufflinks v1.3.0[50]. From 44.7% to 91.2% of the qualified reads were mapped to the genome (**Supplementary Table 16**). For each predicted gene, their transcript isoform diversity (alternative splicing) and expression level (FPKM, fragments per kilobase of exon per million fragments mapped) were calculated using TopHat/Cufflinks[50]. The map-based assembly transcriptome sequences of each sample were called using gffread after expression profile analysis. We generated *de novo* transcripts by single-end and paired-end reads, which were respectively assembled by Velvet-Oases[51-52] and Trinity[34]. The transcripts were annotated, following the same pipeline as W14, and the transcripts that were not aligned to *ab into* predicted gene and Euphorbiaceous protein sequences were extracted for lncRNA anaylsis (**Supplementary Note 21**). A total of 38,965 - 51,300 transcripts were identified among the 12 RNA-seq samples, and 16,755 - 23,379 unique genes were annotated with an average length of 2004.1 to 2632.0 bp. This indicated that we got high-quality RNA-seq data and annotative information. The functional annotation of the transcriptomes was performed, based on the blast results with *Arabidopsis* TAIR 10 (http://www.arabidopsis.org).

Comparative transcriptomes Transcriptome analysis revealed that totally 31,396 genes expressed in 12 samples, a deputy of developing leaf, stem and storage root of W14, KU50 and Arg7. In leaf, 1,071 and 1,782 genes were significantly higher expressed in cultivars KU50 and Arg7, whereas 1,009 to 1,211 genes were higher expressed in wild W14 (**Supplementary Figs. 28a-b and 29)**, respectively. In

storage root with typical developing root at middle stage (MTR), 1,103 and 2,160 genes were higher expressed in cultivars KU50 and Arg7, whereas 2,017 and 2,052 genes were higher expressed in wild W14 (**Supplementary Figs. 28c-d and29**). Of these genes, 406 and 1,690 genes higher co-expressed in leaf and storage root of cultivars, whereas 343 and 1,042 genes higher expressed in wild W14.

Gene Ontology (GO) enrichment analysis of the four gene groups revealed the enhanced or dwindled pathways in evolution. In storage root, the subcategories of GO class of 'cellular component', 'cell part' to 'cytoplasmic part' and 'organelle', and 'response to stimulus' with subcategories of 'response to abscisic acid stimulus', 'response to oxidative stress' and 'response to temperature stimulus', were enriched in cultivated species; but GO class of 'metabolic process', subnets related to 'cell wall polysaccharide biosynthesis process', 'lipid metabolic process' to 'fatty acid metabolic process', 'secondary metabolic process' and 'response to stimulus' with subnets of 'response to chemical stimulus' to 'response to water stress' and 'response to jasmonic acid stimulus' were enriched in wild species (**Supplementary Figure 30A, C**). Meanwhile, in functional leaf, GO subcategories of 'cellular metabolic process', photosynthesis, 'cell part' to 'chloroplast part' and 'photosystem', and 'response to stimulus' with subnets of 'response to heat', 'response to light stimulus', 'response to oxidative stress' and 'response to bacterium and fungus' were expanded in cultivated species; but GO terms of 'transporter activity' to 'potassium ion symporter activity', 'sugar/hydrogen symporter activity' and 'calcium transporting ATPase activity', 'secondary metabolic process' and 'biological regulation' with subcategories of 'localization' to 'ion transport' and 'auxin transport', 'regulation of biosynthesis' and 'positive regulation of flower' were enriched in wild species (**Supplementary Figure 30B, D**). All genes enriched in special pathways are listed in **Supplementary Table17**.

We further calculated the average selection pressure index (*Ka/Ks*) of the genes specifically enriched in GO terms and compared them with each other. The genes with W14>KU50-Arg7 in subcategories had selection pressure indexes ranging from 0.25 to 0.36, and the genes with KU50-Arg7>W14 in subcategories, such as

‘photosynthesis’, ‘cell part’, ‘stimulus response’ and ‘terpenoid metabolic process’, had selection pressure indexes ranging from 0.12 to 0.55. These results indicated that a higher selection pressure took place in the genes significantly increased in expression in cultivars than those significantly increased in expression in wild W14 (**Supplementary Figure 32**).

Supplementary Note 18 Shifts of gene expression pattern in carbon flux

We specially investigated the expression pattern of the genes in photosynthesis (including light reaction and Calvin cycle), major carbon metabolism (including sucrose and starch biosynthesis), cell wall biosynthesis (precursors) and secondary metabolism. According to the Mapman annotation for cultivars compared to wild W14 in storage root and leaf, Mapman images (**Supplementary Figure 32-33**) were used to represent the expression patterns of interested genes in individual pathways. Significantly higher expression patterns of genes for photosynthesis, from photosystem to carbon dioxide fixation in leaf, and genes for sugar transportation and starch synthesis in storage root of cultivars than wild subspecies are shown in **Fig. 2b**. Meanwhile, significantly lower expression patterns of genes for secondary metabolism, cell wall synthesis in storage root of cultivars than wild ancestor were identified (**Supplementary Figure 32 and 33**).

Supplementary Note 19 Comparative analysis of cyanogen biosynthesis

We measured the cyanogenic glucoside contents of roots and leaves of the wild W14 and cultivated KU50 through LC-MS analysis. Five plants were analyzed for each of cultivar. A leaf disc was sampled from the first unfolded leaf of each plant by snap-closing the 2mL-eppendorf lid around one of the leaf fingers, and then another sample was taken in a similar manner from one of the other leaf fingers. The same five plants from which the leaves were sampled were used for tuber extraction. An approximately 0.5 cm-thick slice in the edge of the tuber was cut using a cork borer and transferred into individual 2 mL-Eppendorf tubes. The plant samples were immersed into 300 µl and 500 µl of pre-warmed 85% (v/v) methanol for leaf and tuber, respectively. After closing the tube and securing the lid with a cap lock, the samples were boiled in a water bath at 100 °C for 3 min (leaf) or 5 min (tuber). Then, the MeOH extract was transferred into a new tube, lyophilized to dryness, the dry matter re-suspended in water in a total volume of 200 µl and filtered through a 0.45-µm filter. Analytical LC-MS was carried out using an Agilent 1100 Series LC (Agilent Technologies, Texas, USA) coupled to a Bruker Esquire 3000+ ion trap mass spectrometer (Bruker Daltonics) fitted with an XTerra MS C18 column (Waters; 3.5µM, 2.1 x 100 mm, flow rate 0.2 mL min^{-1}). The mobile phases were as follows: A, 0.1% (v/v) HCOOH and 50 µM NaCl; and B, 0.1% (v/v) HCOOH and 80% (v/v) MeCN. The gradient program was as follows: 0 to 4 min, isocratic 2% (v/v) B; 4 to 10 min, linear gradient 2% to 8% B; 10 to 30 min, linear gradient 8% to 50% (v/v) B; 30 to 35 min, linear gradient 50% to 100% (v/v) B; and 35 to 40 min, isocratic 100% B. The mass spectrometer was run in positive ion mode. Traces of total ion current and extracted ion currents for specific $[M + Na]^+$ adduct ions were used to identify selected peaks. The retention time for linamarin and for lotaustralin was 5.5 and 15.8 min, respectively. The results are listed in **Supplementary Table 18**.

Supplementary Note 20 Validation of differnatial expressions of key genes by real-time qPCR

The expression patterns of a set of 12 genes for sucrose transport and starch synthesis were validated by real-time quantitative PCR between wild ancestor W14 and two cultivated varieties, KU50 and Arg7. Five samples, including early and functional leaves and tuber roots at three different developmental stages (60, 120 and 180-200 DAP, -Days After Planting), of each genotype were tested in this experiment. Total RNA was extracted using RNAplant reagent (Tiangen, Beijing, CHN) and purified using RNeasy Plant Mini Kit (Qiagen, Valencia, CA). First-strand cDNA was generated from ~5 μg of total RNA using the RevertAid H Minus First Strand cDNA Synthesis Kit (Fermentas) according to manufacturer's instructions. Real-time qPCR was performed using a standard SYBR Premix Ex TaqTM kit (TaKaRa DRR041) with Rotor-gene 6000. The target genes and the control β-Actin gene were amplified with three biological replications. The values of the threshold cycle (CT) were calculated using Rotor-Gene 6000 series software 1.7 (Corbett Robotics, Australia). The CT values were converted to relative expression by the ΔΔCT method with the following formula: The relative concentration=$2^{-\Delta\Delta CT}$, where $\Delta\Delta CT = (\Delta CT_{sample} - \Delta CT_{control})$, ΔCT= CT(target gene)-CT (Actin) in each sample.

The expression folds of 12 genes in tuber root of KU50 and Arg7 to W14 are shown in **Supplementary Figure 34**. The comparative fold changes of KU50/W14 and Arg7/W14 at all three developmental stages studied, 60 d, 150 d and 210 d, were detected for all 12 genes, including *SUSY*, *SSS*, *AGPase*, *SSS*, *ALDO*, *HXK*, *PGMP*, *FRU*, *PGI*, *PGMC*, *GBSS* and *CWI*, with a range from 0.16 to 957. These results coincided with the transcriptome expression patterns and provided further evidence for starch synthesis model in cassava.

Supplementary Note 21 Micro RNA and non-coding RNA annotation

We searched for novel miRNAs in the three cassava genomes, W14, KU50 and AM560 using the corresponding small RNA-seq datasets, respectively[53]. The method for Cassava miRNA identification has been documented previously[54]. Briefly, we first processed raw sequence reads by an in-house method that recursively searches for the longest substring of the adaptor appearing within a sequence read. Qualified reads, the ones carrying 3' sequencing adaptor and being longer than 17-nt, were then mapped to a genome using Bowtie (version 0.12.7). The loci with a sufficient number of reads mapped to were subject to miRNA identification with stringent criteria, including presence of a hairpin structure and a 21-nt RNA duplex with 3'-nt overhang. The newly identified miRNAs and known miRNAs in cassava were characterized in details in previous studies[53]. We further carried out a homology search by aligning the mature and hairpin sequences of the miRNAs to the three cassava genomes, respectively, using the local alignment tool BLAST. We set the *p*-value obtained from BLAST less than 1e-10 and manually examined the alignment to determine if a hit of BLAST was homologous to the input miRNA. We mapped the qualified reads from the corresponding genome datasets to the identified homologous sequences by Bowtie and counted the map-able reads. If no homologous sequences could be identified in a genome assembly, we then mapped the reads from the sequencing datasets of the same genome to the input miRNA sequence, with allowing two mismatches. We considered a miRNA not conserved in the genome assembly, if both criteria were met: 1) no homologous sequences identified and 2) no sufficient mappable reads (less than 10 normalized reads from the sample of the cultivar; reads were divided by the number of mappable reads in each sample to adjust for variation across samples. More details were presented in the previous work[53]. If there were reads mapped to the input miRNA sequences, we considered the miRNA conserved in the genome assembly, even if we were not able to identify the homologous sequences.

The other noncoding RNA genes were analyzed using existing tools. In particular,

tRNAs were analyzed using tRNASCAN-SE[55] (Version 1.23); rRNAs were identified by RepeatMasker (Version open 3.3.0) with cloned 18S, 5.8S, 26S and 5S rDNA sequences of full-length KU50 as the library; and the other types of RNAs were detected by INFERNAL[56] (version 1.1) with cm models downloaded from Rfam database (Version 11.0, URLs). In addition, putative long non-protein coding RNAs (lncRNAs)[57-62] were detected by transcriptome analysis via the assembled transcripts generated by RNA-seq over than 200 bp that were not aligned to the coding regions of *ab initio* predicted genes and Euphorbiaceous protein sequences. We then re-aligned these transcripts to the draft genomes, detected their potential exon structures by transcript global alignment information, merged them, if there are overlapped. In total, 128,782 lncRNAs with an average size of 417 bp were identified for W14 and 188,344 lncRNAs with an average size of 613 bp were identified for KU50 (**Supplementary Table 20**).

Supplementary Note 22 CIS element analysis of the *SUSY*, and *PPDK* gene promoters

The expression profile of the SUSY and PPDK genes The overall gene profiling showed that the *SUSY* and *PPDK* genes are the import nodes in starch metabolism network. However, the *SUSY* gene has many copies in most plant species. So, we first checked out the copy numbers of these two genes and found the entire candidate transcripts annotated into *SUSY* and *PPDK*. We could identify the copy numbers of the *SUSY* gene and the number of transcripts of every copy of it by checking GTF files generated by Cufflinks[50]. The expression profiles of the two genes are shown in **Supplementary Figure 37.**

Identification of the promoter regions of SUSY and PPDK The similarity sequences of *SUSY* and *PPDK* in Malpighiales and *A. thaliana* in GenBank (URLs) were gathered

by BLASTN and multiple aligned by ClustalW2. The maximum parsimony tree was constructed for the orthologs detected using MEGA 5.0. The CDS annotation information of *Arabidopsis* and other annotated sequences was used to detect the start codon position of cassava sequences. One thousand base pairs of the nucleotide sequence before start codon were extracted as the promoter region of the gene.

By searching the public database, we found that *PPDK* is a single-copy gene in the other sequenced plant genomes. *SUSY* is a multiple-copy gene and the copy number of it is different in the sequenced plant genomes. *SUSY* had six copies in the *Arabidopsis* genome, seven copies in the *Populus* genome and five copies in the *Ricinus* genome. We found six copies of the *SUSY* gene in the cassava genome. The relationships of the orthologs between different species are shown in **Supplementary Figure 38**.

Comparison of the promoter regions of SUSY and PPDK genes in three cassava cultivars The ortholog sequences of the two genes in the three cassava genomes, KU50, AM560 and W14, were multiple-aligned by ClustalW2. Then, the promoter regions were analyzed using MEME Suite for cis-motif prediction. The patterns within a multiple sequence alignment of motif were generated by WebLogo.

Identification of binding sites for several transcription factors mediated by specific miRNAs in the promoter region of *SUSY* Based on the assembled and cloned sequences, we obtained the upstream sequences of *SUSY* gene including its promotor in a length of 1,087 bp, 1,088 bp and 1,068 bp from the AM560, KU50 and W14 genomes, respectively. The binding motifs of *MYB*, *ARF* and *NF-YA3* were found in the upstream regions of *SUSY* (**Supplementary Figure 40**). These three transcription factors have been already verified to be regulated by miR159, miR166 and miR156, respectively (**Supplementary Data 9**).

Supplementary Note 23 Comparative analysis of predicted proteins between wild ancestor and cultivars

The coding DNA sequences (CDS) of 165 genes involved in photosynthesis, Calvin cycle, and sucrose and starch synthesis from the draft genomes of wild W14 and cultivars KU50 and AM560 were used to produce predicted proteins through an on-line program ORF Finder (http://www.ncbi.nlm.nih.gov/gorf). Referenced to the protein sequences predicted from AM560, comparative analysis of the predicted protein sequences between KU50 and W14 was carried out using software DNAMAN (version 6.0). The amino-acid differences between different varieties were calculated into percentages. The heat-maps were generated from the data of amino-acid differences using the R program (version 3.0). As with tree maps, the rectangular regions in a mosaic plot were hierarchically organized.

We found a higher diversity of protein sequences in the three metabolic pathways between wild W14 and cultivar AM560 than between cultivars KU50 and AM560 (**Supplementary Table 21; Supplementary Figs. 41, 42 and 43**). These results supported the increased starch accumulation model in cultivated cassava.

4) Supplementary References

1. Schaal BA, Olsen KM. Gene genealogies and population variation in plants, *Proc Natl Acad Sci USA* **97**, 7024-7029 (2000).
2. Colombo C, Second G,and A Charrier. Genetic relatedness between cassava (Manihot esculenta Crantz) and M. flabellifolia and M. peruviana based on both RAPD and AFLP markers, *Genetics and Molecular biology* **23**, 417-423(2000).
3. Kenneth M. Olsen and Barbara A. Schaal. Microsatellite variation in cassava (Manihot esculenta, Euphorbiaceae) and its wild relatives: further evidence for a southern Amazonian origin of domestication, *Am J Bot* **88**, 131-142 (2001).
4. Allem, A.C. The origin and taxonomy of cassava. In Cassava: Biology, Production and Utilization; Hillocks, R.J., Thresh, J.M., Bellotti, A.C., Eds. CAB International: Oxford, UK, 1-16 (2001).
5. Lathrap, D.W. The Upper Amazon. Thames and Hudson, London (1970).
6. Gibbons, A. New view of early Amazonia. *Science* **248**, 1488-1490 (1990).
7. Rogers, DJ and Appan, SG. Manihot and Manihotoides (Euphorbiaceae). A computer-assisted study. Flora Neotropica, Monograph No. 13. Hafner Press, New York (1973).
8. Tao, Q., Wang, A. & Zhang, H.-B. One large-insert plant transformation-competent BIBAC library and three BAC libraries of Japonica rice for genome research in rice and other grasses. *Theor Appl Genet* **105**, 1058-1066 (2002).
9. Luo, M. et al. High-throughput fingerprinting of bacterial artificial chromosomes using the SNaPshot labeling kit and sizing of restriction fragments by capillary electrophoresis. *Genomics* **82**, 378-389 (2003).
10. Nelson, W. M. et al. Efficacy of clone fingerprinting methodologies. *Genomics* **89**,160-165 (2007).
11. Gu, Y.Q. et al. Construction of physical map for Brachypodium distachyon and its comparative analysis with rice. *BMC Genomics* **10**,496 (2009).

12. You, F. et al. GenoProfiler: batch processing of high throughput capillary fingerprinting data. *Bioinformatics* **23**, 240-242 (2007).
13. Soderlund, C., Humphray, S., Dunham, I. & French, L. Contigs built with fingerprints, markers, and FPC V4.7. *Genome Res* **11**, 934-941 (2000).
14. Li, Z. et al. Comparison of the two major classes of assembly algorithms: overlap-layout- consensus and de-bruijn-graph. *Briefings in Functional Genomics* **11**, 25-37 (2012).
15. Luo, R. et al. SOAPdenovo2: an empirically improved memory-efficient short-read de novo assembler. *Giga Sci* **1**, 18 (2012).
16. Quinn, N. L. et al. Assessing the feasibility of GS FLX Pyrosequencing for sequencing the Atlantic salmon genome. *BMC Genomics* **9**, 404 (2008).
17. Mullikin, J. C. The Phusion Assembler. *Genome Res* **13**, 81-90 (2002).
18. Huang X. & Madan A. CAP3: A DNA Sequence Assembly Program. *Genome Res***9**, 868-877 (1999).
19. Li, H. & Durbin, R. Fast and accurate short read alignment with Burrows-Wheeler transform. *Bioinformatics* **25**, 1754-1760 (2009).
20. McKenna, A. et al. The Genome Analysis Toolkit: A MapReduce framework for analyzing next-generation DNA sequencing data. *Genome Res* **20**, 1297-1303 (2010).
21. DePristo, M. A. et al. A framework for variation discovery and genotyping using next-generation DNA sequencing data. *Nat Genet* **43**, 491-498 (2011).
22. Kent, W. J. BLAT- The BLAST-Like Alignment Tool. *Genome Res* **12**, 656–664 (2002).
23. van Oeveren, J. et al. Sequence-based physical mapping of complex genomes by whole genome profiling. *Genome Res* **21**, 618-625 (2011).
24. Soderlund, C., Humphray, S., Dunham, I. & French, L. Contigs built with fingerprints, markers, and FPC V4.7. *Genome Res* **11**, 934-941 (2000)
25. Nelson, W. & Soderlund, C. Integrating sequence with FPC fingerprint maps. *Nucleic Acids Res***37**, e36-e36 (2009).

26. Engler, F. W. Locating sequence on FPC maps and selecting a minimal tiling path. *Genome Res* **13**, 2152-2163 (2003).
27. Stanke, M. & Waack, S. Gene prediction with a hidden Markov model and a new intron submodel. *Bioinformatics* **19**, ii215-ii225 (2003).
28. Korf, I. Gene finding in novel genomes. *BMC Bioinformatics* **5**, 59 (2004).
29. Ter-Hovhannisyan, V., Lomsadze, A., Chernoff, Y. O. & Borodovsky, M. Gene prediction in novel fungal genomes using an ab initio algorithm with unsupervised training. *Genome Res* **18**, 1979-1990 (2008).
30. Burge, C. & Karlin, S. Prediction of complete gene structures in human genomic DNA. *J Mol Biol* **268**, 78-94 (1997).
31. Gremme, G., Brendel, V., Sparks, M. E. & Kurtz, S. Engineering a software tool for gene structure prediction in higher organisms. *Information and Software Technology* **47**, 965-978 (2005).
32. Haas, B. J. Improving the Arabidopsis genome annotation using maximal transcript alignment assemblies. *Nucleic Acids Res* **31**, 5654-5666 (2003).
33. Wu, T. D. & Watanabe, C. K. GMAP: a genomic mapping and alignment program for mRNA and EST sequences. *Bioinformatics* **21**, 1859-1875 (2005).
34. Grabherr, M. G. et al. Full-length transcriptome assembly from RNA-Seq data without a reference genome. *Nat Biotechnol* **29**, 644-652 (2011).
35. Haas, B. J. et al. Automated eukaryotic gene structure annotation using EVidenceModeler and the Program to Assemble Spliced Alignments. *Genome Biol* **9**, R7 (2008).
36. Altschul, S. F. et al. Gapped BLAST and PSI-BLAST: a new generation of protein database search programs. *Nucleic Acids Res* **25**, 3389-3402 (1997).
37. Kanehisa, M., Goto, S., Sato, Y., Furumichi, M. & Tanabe, M. KEGG for integration and interpretation of large-scale molecular data sets. *Nucleic Acids Res* **40**, D109-D114 (2011).
38. Kanehisa, M. & Goto, S. KEGG: Kyoto Encyclopedia of Genes and Genomes. *Nucleic Acids Res* **28**, 27-39 (2000).

39. The UniProt Consortium. Ongoing and future developments at the Universal Protein Resource. *Nucleic Acids Res* **39**, D214-D219 (2010).
40. Tatusov, R. L. et al. The COG database: an updated version ncludes eukaryotes. *BMC Bioinformatics* **4**, 41 (2003).
41. Punta, M. et al. The Pfam protein families database. *Nucleic Acids Res* **40**, D290-D301 (2011).
42. Zdobnov, E. M. & Apweiler, R. InterProScan - an integration platform for the signature- recognition methods in InterPro. *Bioinformatics* **17**, 847–848 (2001).
43. Ye, J. et al. WEGO: a web tool for plotting GO annotations. *Nucleic Acids Res* **34**, W293-W297 (2006).
44. Albers, C. A. et al. Dindel: Accurate indel calls from short-read data. *Genome Res* **21**, 961-973 (2011).
45. Drummond, A. J. & Rambaut, A. BEAST: Bayesian evolutionary analysis by sampling trees. *BMC Evol Biol* **7**, 214 (2007).
46. Bell, C. D., Soltis, D. E. & Soltis, P. S. The age and diversification of the angiosperms re-revisited. *Am J Bot* **97**, 1296-1303 (2010).
47. Langmead, B., Trapnell, C., Pop, M. & Salzberg, S. L. Ultrafast and memory-efficient alignment of short DNA sequences to the human genome. *Genome Biol* **10**, R25 (2009).
48. Trapnell, C., Pachter, L. & Salzberg, S. L. TopHat: discovering splice junctions with RNA-Seq. *Bioinformatics* **25**, 1105-1111 (2009).
49. Kim, D. et al. TopHat2: accurate alignment of transcriptomes in the presence of insertions, deletions and gene fusions. *Genome Biol* **14**, R36 (2013).
50. Roberts, A. et al. Differential gene and transcript expression analysis of RNA-seq experiments with TopHat and Cufflinks. *Nat Protoc* **7**, 562–578 (2012).
51. Zerbino, D. R. & Birney, E. Velvet: Algorithms for de novo short read assembly using de Bruijn graphs. *Genome Res* **18**, 821-829 (2008).

52. Schulz, M. H., Zerbino, D. R., Vingron, M. & Birney, E. Oases: robust de novo RNA-seq assembly across the dynamic range of expression levels. *Bioinformatics* **28**, 1086-1092 (2012).
53. Zeng, C. et al. Conservation and divergence of microRNAs and their functions in Euphorbiaceous plants. *Nucleic Acids Res* **38**, 981-995 (2010).
54. Zhang W, et al. Multiple distinct small RNAs originate from the same microRNA precursors, *Genome Biology* **11**, R81 (2010).
55. Lowe, T. M. & Eddy, S. R. tRNAscan-SE: a program for improved detection oftransfer RNA genes in genomic sequence. *Nucleic Acids Res* **25**, 955-964 (1997).
56. Nawrocki, E. P., Kolbe, D. L. & Eddy, S. R. Infernal 1.0: inference of RNA alignments. *Bioinformatics* **25**, 1335-1337 (2009).
57. The FANTOM Consortium. The Transcriptional Landscape of the Mammalian Genome. *Science* **309**, 1559-1563 (2005).
58. Birney, E. et al. Identification and analysis of functional elements in 1% of the human genome by the ENCODE pilot project. *Nature* **447**, 799-816 (2007).
59. Guttman, M. et al. Chromatin signature reveals over a thousand highly conserved large non-coding RNAs in mammals. *Nature* **457**, 223-227 (2009).
60. Guttman, M. et al. Ab initio reconstruction of cell type–specific transcriptomes in mouse reveals the conserved multi-exonic structure of lincRNAs. *Nat Biotechnol* **28**, 503-510 (2010).
61. Guttman, M. et al. lincRNAs act in the circuitry controlling pluripotency and differentiation. *Nature* **477**, 295-300 (2012).
62. Trapnell, C. et al. Transcript assembly and quantification by RNA-Seq reveals unannotated transcripts and isoform switching during cell differentiation. *Nat Biotechnol* **28**, 516-520 (2010).
